# Hydrogenation of Secondary Amides using Phosphane Oxide and Frustrated Lewis Pair Catalysis

**DOI:** 10.1002/chem.202100041

**Published:** 2021-09-02

**Authors:** Laura Köring, Nikolai A. Sitte, Markus Bursch, Stefan Grimme, Jan Paradies

**Affiliations:** ^1^ Chemistry Department Paderborn University Warburger Strasse 100 D-33098 Paderborn Germany; ^2^ Mulliken Center for Theoretical Chemistry Institute of Physical and Theoretical Chemistry Rheinische Friedrich-Wilhelms-Universität Bonn Beringstr. 4 D-53115 Bonn Germany

**Keywords:** carboxylic amide, frustrated Lewis pair, hydrogenation, hydrogen, phosphane oxide

## Abstract

The metal‐free catalytic hydrogenation of secondary carboxylic acid amides is developed. The reduction is realized by two new catalytic reactions. First, the amide is converted into the imidoyl chloride by triphosgene (CO(OCCl_3_)_2_) using novel phosphorus(V) catalysts. Second, the in situ generated imidoyl chlorides are hydrogenated in high yields by an FLP‐catalyst. Mechanistic and quantum mechanical calculations support an autoinduced catalytic cycle for the hydrogenation with chloride acting as unusual Lewis base for FLP‐mediated H_2_‐activation.

## Graphical abstract

**The hydrogenation of** secondary carboxylic acid amides by combined phosphane oxide and frustrated Lewis pair catalysis is described. The reaction proceeds under mild conditions and displays excellent functional group tolerance. Kinetic and quantum‐chemical experiments support the autoinduced catalytic cycle with chloride as active Lewis base for the hydrogen activation.

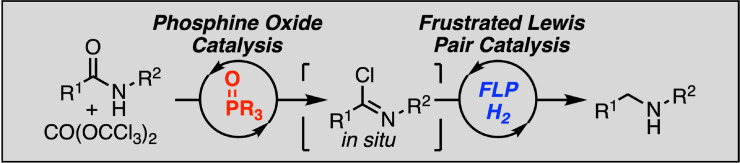

The acylation of primary and secondary amines serves as a highly efficient method for the construction of simple secondary and tertiary amides, complex molecules,^[1]^ natural products^[2]^ and in late stage macrocyclizations.^[3]^ Such readily available key intermediates are valuable starting points for the selective synthesis of secondary and tertiary amines. Therefore, the reduction of carboxamides is a key transformation for the selective synthesis of symmetrical and unsymmetrical amines.^[4]^ The reduction can be realized using stoichiometric amounts of strongly reducing metal hydrides but these reagents suffer from major drawbacks such as limited functional group tolerance and require a rigorous protection group strategy. Metal catalyzed hydrogenations of amides are by far the most atom economic reductions^[5]^ but require drastic conditions.^[6]^ Recently, milder reaction conditions were realized (Scheme 1),[^7^, ^8^, ^9^, ^10^, ^11^] however selectivity issues due to C−N bond cleavage of hemiacetals^[9],[12]^ or exhaustive reduction of functional groups such as alkenes and alkynes are still prevailing problems. The hydrosilylation of amides facilitates the reduction by the removal of the oxygen atom as siloxane, but comes at the cost of atom efficiency (Figure 1A and B). Nonetheless, high tolerance towards hydrogenation susceptible functionalities was achieved with inorganic[^13^, ^14^, ^15^, ^16^, ^17^, ^18^, ^19^] and organic[^20^, ^21^, ^22^, ^23^, ^24^] catalysts. Recently, we reported the highly effective hydrogenation of tertiary amides using an FLP‐catalyst[^25^, ^26^, ^27^] and oxalyl chloride as low molecular weight deoxygenation reagent.^[28]^ However, the hydrogenation of secondary amides remained a major challenge. Thus, a new strategy for the hydrogenation of secondary amides is required.

**Scheme 1:**
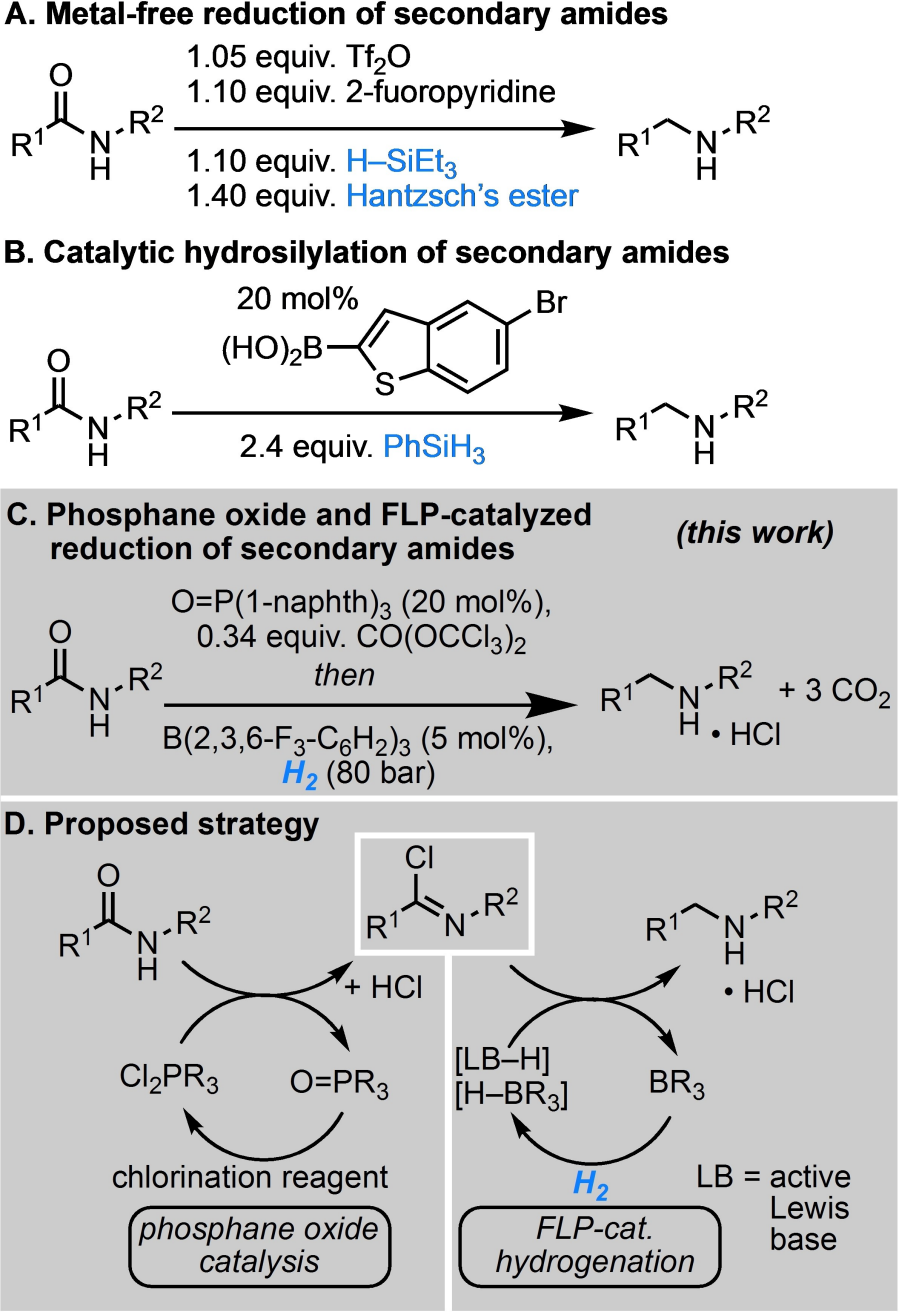
Metal‐free reductions of secondary carboxamides.

**Figure 1:**
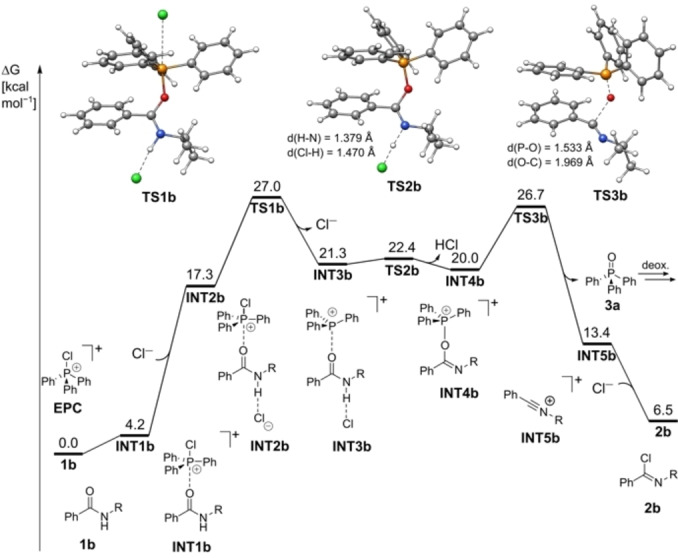
Free energy diagram of the conversion of carboxamides to imidoyl chlorides at the PW6B95‐D4/def2‐QZVP+COSMO‐RS(CHCl_3_)//PBEh‐3c(COSMO(CHCl_3_)) level of theory. All free energies in kcal/mol.

We envisioned a new phosphane oxide catalyzed conversion of the sec. amide to the imidoyl chloride, which is subsequently reduced with H_2_ in the presence of an FLP catalyst (Figure 1D). This approach required the development of two new catalytic reactions with particular consideration of compatible conditions.

First, we set out to investigate the efficient formation of the imidoyl chloride which will later serve as in situ formed substrate for the FLP‐catalyzed hydrogenation. We observed that SOCl_2_ cleanly converts the amide **1 a** into the imidoyl chloride **2 a** in excellent yields under mild conditions (Table 1, entry 1).[^29^, ^30^] However, SOCl_2_ was incompatible with the conditions of the FLP‐catalyzed hydrogenation. The addition of (COCl)_2_ to the amide **1 a** furnished mostly acylation products (entry 2). Alternatively, in situ generated dichlorotriphenyl phosphorane[^31^, ^32^, ^33^] is a known dehydrating reagent.[^34^, ^35^] We investigated the conversion of **1 a** into **2 a** in the presence of 1.0 equiv. O=PPh_3_ and indeed, **2 a** was obtained in 93 % yield (entry 3). The catalytic version with (COCl)_2_ could not be elaborated because the acylation could not be outcompeted by the phosphane oxide reaction (entry 4).

**Table 1:** Phosphine oxide catalyzed imidoyl chloride formation.

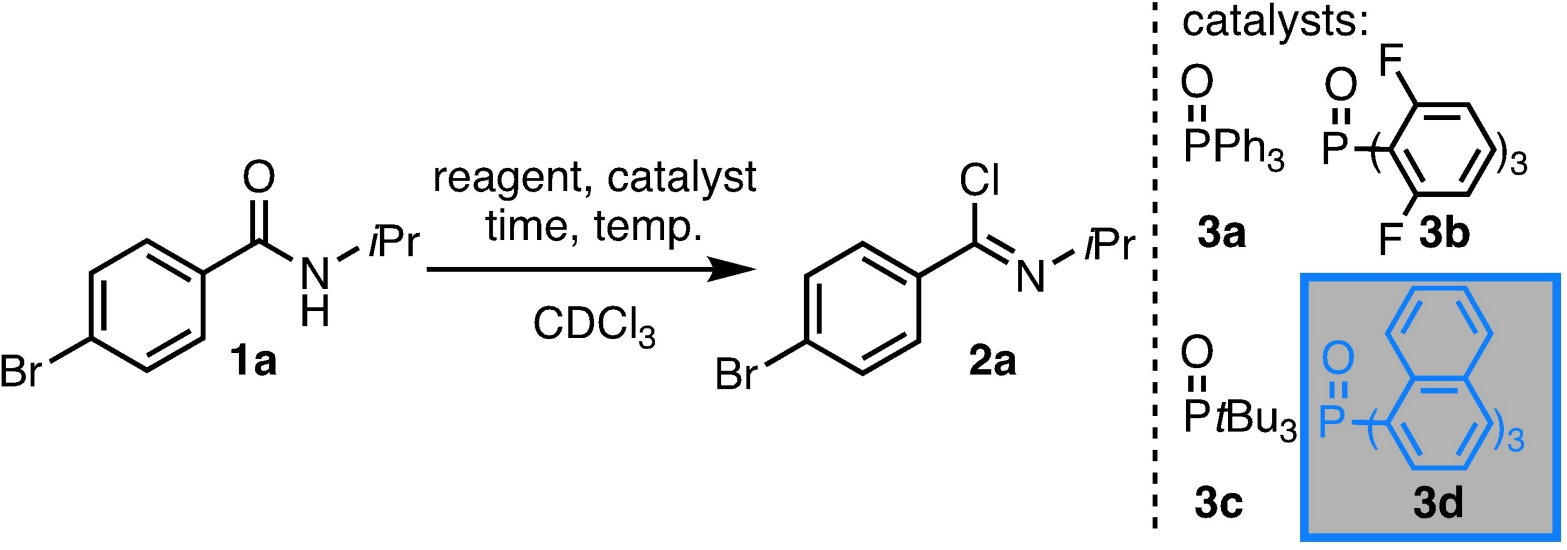					
entry	reagent (equiv.)	cat. (mol %)	temp. [°C]	time [h]	yield^[a]^ [%]
1	SOCl_2_ (10)	–	70	3	>98
2	(COCl)_2_ (1.1)	–	70	18	0^[b]^
3	“	**3 a** (100)	70	110	93
4	“	**3 a** (20)	70	18	20^[c]^
5	CO(OCCl_3_)_2_ (0.4)	–	70	18	<20
6	“	**3 a** (20)	70	34	>98 (>98)^[d][e]^
7	“	**3 b** (20)	70	18	80 (90)^[d]^
8	“	**3 c** (20)	70	18	<30 (55)^[d]^
9	“	**3 d** (20)	70	42	85
10	“	**3 d** (20)	90	18	>98

[a] determined by ^1^H NMR spectroscopy; [b] contaminated with >98 % of acylation product; [c] contaminated with 5 % **1 a** and 75 % of acylation product; [d] performed at 90 °C; [e] 18 h.

We found with triphosgene (CO(CCl_3_)_2_) a selective alternative for the conversion of O=PPh_3_ to PPh_3_Cl_2_ in shorter reaction times without noticeable background reaction (Table 1, entry 6). The catalytic conversion of **1 a** by CO(CCl_3_)_2_ is achieved in the presence of all four investigated phosphane oxides (O=PPh_3_ (**3 a**), O=P(2,6‐F_2_‐C_6_H_3_)_3_ (**3 b**), O=P*t*Bu_3_ (**3 c**) and O=P(1‐Naphth)_3_ (**3 d**), entries 6–9). Up to >98 % yield within 18 h was obtained at 90 °C. Among the investigated phosphine oxides, **3 d** preformed best (entry 10) and displayed in contrast to **3 a** and **3 b** no interference with the borane **4 e** in the FLP‐catalyzed hydrogenation (see below).^[36]^

Whereas the mechanism of the phosphane oxide‐dichlorophosphorane interconversion is known,^[33]^ the subsequent reaction with sec. amides to the imidoyl chloride is so far unreported. Quantum‐mechanical calculations at the PW6B95‐D4/def2‐QZVP+COSMO‐RS(CHCl_3_)//PBEh‐3c(COSMO(CHCl_3_))[^37^, ^38^, ^39^, ^40^, ^41^, ^42^, ^43^, ^44^, ^45^, ^46^] level of theory level (for details see the Supporting Information) identify the S_N_2‐type addition of the amide oxygen (**TS1 b**) to the chloro phosphonium ion as rate determining step (Figure 1), with a reaction barrier of 27.0 kcal/mol under chloride loss and formation of **INT3 b**. Deprotonation of **INT3 b** by a chloride anion yields **INT4 b** via **TS2 b** (22.4 kcal/mol). Phosphane oxide **3 a** is reformed via **TS3 b** (26.7 kcal/mol), yielding **2 b** after chloride addition. The overall free reaction energy amounts to 6.5 kcal/mol, indicating that the subsequent deoxygenation of the formed phosphane oxide (not shown in Figure 1), which is known to be strongly exergonic, is needed as driving force.^[33]^ The generally high reaction barriers qualitatively agree with the necessity of heating the reaction mixture and relatively slow reactions. This observation is supported by the increased reactivity of the fluorinated aryl phosphane **3 b** compared to **3 a** or **3 c**. The steric restrictions also play a significant role due to the back‐side attack of the amide to the phosphorane in order to form the P−O bond.

The second challenge of the amide reduction is the FLP‐catalyzed hydrogenation of the imidoyl chloride intermediate. We investigated the catalytic hydrogenation of **2 b** with a series of the five boranes **4 a**–**e**, comprising Lewis acidities between 100 % and 82 %. Treatment of **2 b** (0.16 M) in chloroform with H_2_ (4 bar) at 70 °C in the presence of 20 mol % of the two strongest Lewis acids **4 a** and **4 b** in our study resulted in essentially no conversion as a consequence of catalyst inhibition. However, the application of the weaker Lewis acid **4 c** provided the product **5 b** as hydrochloride salt in 53 % yield (Table 2, entries 1–3). Further decrease of the Lewis acidity to 82 % (**4 d**, entry 4) resulted in inefficient H_2_‐activation by the imidoyl chloride/borane Lewis pair and caused the drastic decrease of the catalytic productivity.^[28]^ The Lewis acid **4 e** with an acidity between 87 % and 98 % provided an even more efficient catalyst, which produced the product in quantitative yield (entry 5). Moreover, the catalyst loading was reduced to 5 mol % employing 80 bar H_2_ without yield depletion in 20 h (entry 6).

**Table 2:** FLP‐catalyzed hydrogenation of imidoyl chloride 2b.

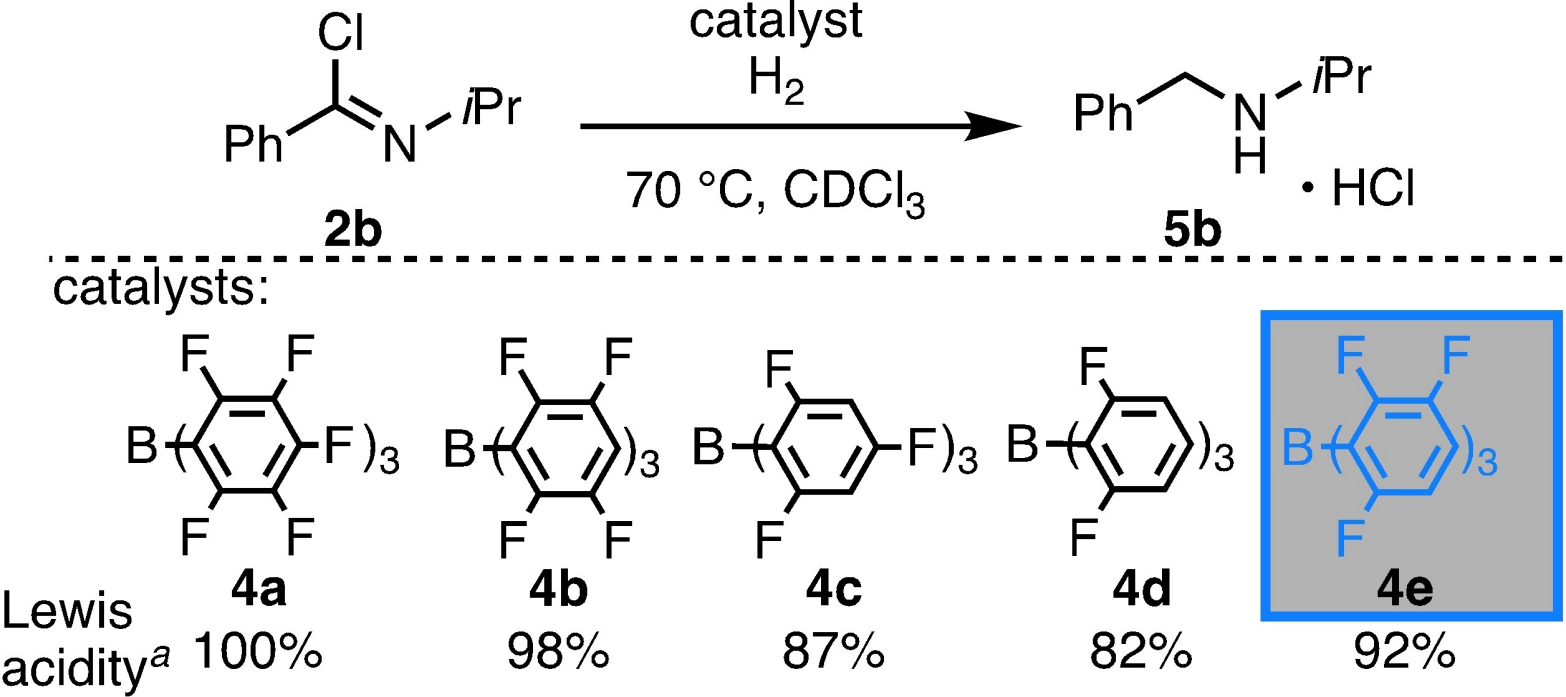				
entry	cat. (mol %)	H_2_ [bar]	time [h]	yield [%]^*b*^
1	**4 a** (20)	4	90	traces
2	**4 b** (20)	4	“	0
3	**4 c** (20)	4	“	53
4	**4 d** (20)	4	“	12
5	**4 e** (20)	4	“	99
6	**4 e** (5)	80	20	99

[a] according to Gutmann‐Beckett with B(C_6_F_5_)_3_ referenced to 100 % Lewis acidity;[^47^, ^48^, ^49^, ^50^, ^51^] [b] determined by ^1^H NMR spectroscopy.

Finally, we successfully merged both catalytic processes in a one‐pot hydrogenation of sec. amides. The carboxylic amide was reacted with 0.34 equiv. CO(COCl_3_)_2_ in the presence of 20 mol % **3 d** at 90 °C for 0.5–15 h. Then, 5 mol % **4 e** dissolved in chloroform was added and the reaction was pressurized with H_2_ (80 bar) for 18 h at 70 °C.^[52]^ A series of benzamides bearing aliphatic, benzylic and aromatic *N‐*substituents were hydrogenated in good to excellent yields (Table 3, 58–95 %).

**Table 3:**
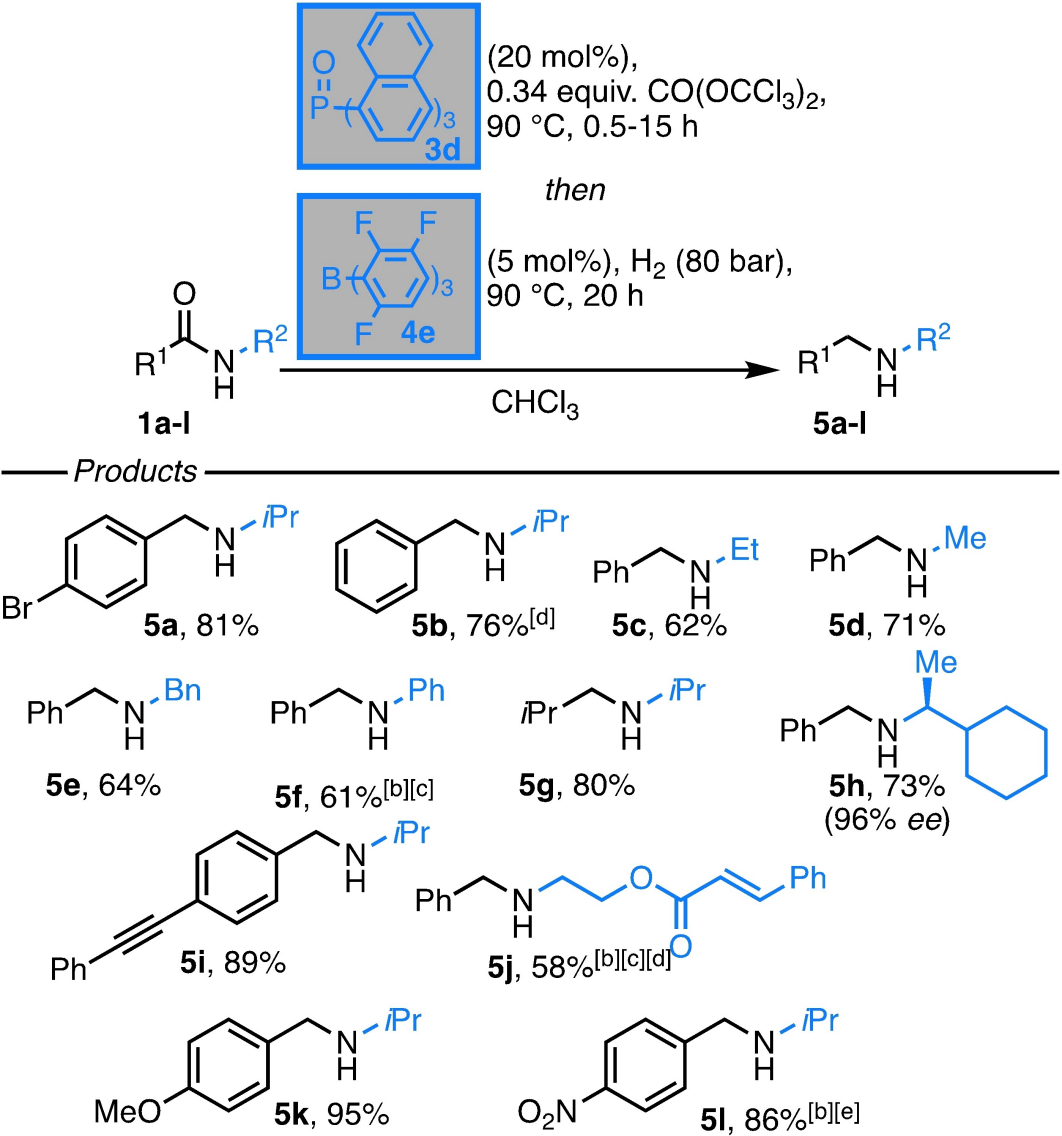
Catalytic hydrogenation of secondary amides.^[a]^

[a] Reactions were typically performed with 1 equiv. of **1** in CDCl_3_ (0.83 M), 0.34 equiv. CO(OCCl_3_)_2_, 20 mol % **3 d** at 90 C °C for 0.5‐15 h, followed by the addition of 5 mol % **4 e** in CHCl_3_ giving a 0.17 M solution, H_2_ (80 bar), 90 °C for 20 h; [b] 10 mol % **4 e**; [c] 1.0 equiv. 2,6‐lutidine added, [d] performed at 70 °C; [e] 40 h.

The reaction displayed excellent functional group tolerance, resulting in the high yielding hydrogenation of bromide **5 a**, ester **5 j**, ether **5 k** and even the nitro compound **5 l**. Notably, α,β‐unsaturated esters and alkynes remained fully intact. The chiral amide **1 h** was reduced in 73 % yield with full conservation of the stereochemical purity. *N*‐phenyl substitution of the benzoic amide (**1 f**) required the application of 2,6‐lutidine as auxiliary Lewis base and the product **5 f** was obtained in 61 % yield. Furthermore, amines with small *N*‐substituent (**5 c**, **5 d**) were accessible in high yields, which is so far unprecedented in FLP‐catalyzed imine hydrogenation.[^53^, ^54^, ^55^, ^56^]

Finally, we investigated the hydrogenation mechanism by kinetic and quantum mechanical experiments. The reaction profile of the catalytic hydrogenation of **2 b** with 10 mol % **4 e** in CDCl_3_ (0.16 M) and 4 bar H_2_ displayed an induction period (Figure S1). This increase of the reaction rate over the first three hours suggests the slow activation of H_2_ by the FLP consisting of **4 e** and **2 b** (compare Scheme 2, simple catalytic cycle). The imidoyl chloride salt [**2 b**−H][H−**4 e**] collapses to the corresponding iminium salt [**6 b**−H][Cl−**4 e**], which is hydrogenated by the Cl^−^/**4 e** FLP to yield **5 b** ⋅ HCl salt as uncompetitive Lewis base for H_2_‐activation. These last two steps can be considered as fast, since neither **6 b** nor the intermediates [**2 b**−H][H−**4 e**] and [**6 b**−H][Cl−**4 e**] were detected by NMR spectroscopy. The preferred splitting of H_2_ by Cl^−^/**4 e** in contrast to **2 b**/**4 e** is supported by the difference of the p*K*_a_ of the conjugate acids (p*K*_a_ ([H−**2 b**]^+^)=5, pK_a_ (HCl)=10.3)^[28]^ as a measure for the activation potency.[^57^, ^58^, ^59^] A second, autoinduced, catalytic cycle is opened up with a lower barrier for the H_2_ activation (see below) as a result of the slow increase of Cl^−^. The autocatalysis is supported by the drastic rate increase when 20 mol % of the product **2 b** ⋅ HCl is added at the beginning of the reaction (Figure S1). In contrast to the FLP‐catalyzed imine hydrogenation,^[58]^ the imidoyl chloride hydrogenation displays saturation kinetic and follows zeroth‐rate order after equal amounts of the product compared to the borane are generated (Figure S2). This indicates that the chloride anion is reversibly bound to the borane, which is in concert with the observed broadening in the ^11^B and ^19^F NMR spectra (see Supporting Information). Free energy calculations reveal that the heterolytic splitting of H_2_ by the FLP **2 b**/**4 e** to the chloro iminium hydroborate [**2 b**−H][H−**4 e**] is endergonic by 9.7 kcal/mol over a barrier of 25.5 kcal/mol (**TS4 b**, simple catalytic cycle, Scheme 2 and Figure 2 left).

**Scheme 2:**
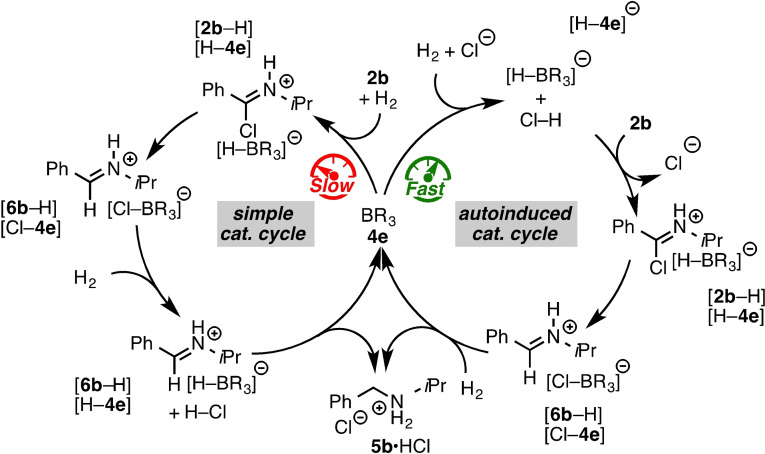
Proposed catalytic cycle of the FLP‐catalyzed hydrogenation of imidoyl chlorides.

**Figure 2:**
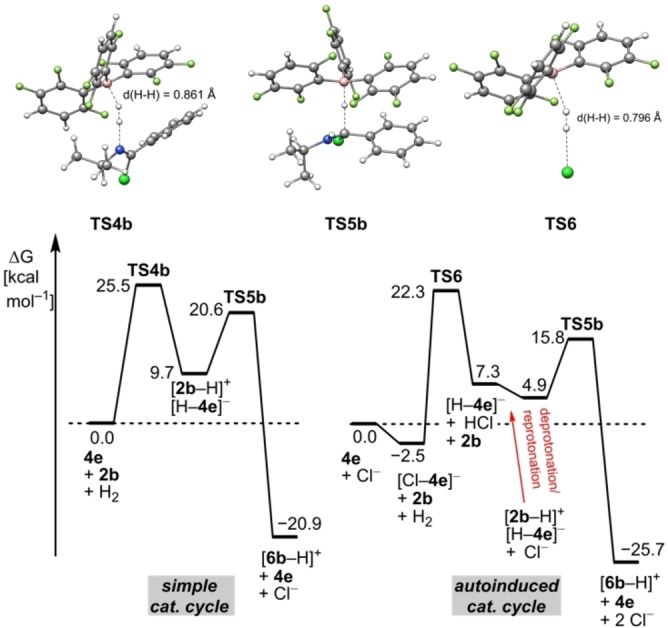
Simplified free energy diagrams of the H_2_‐activation by the FLPs **2 b**/**4 e** and Cl^−^/**4 e** at the PW6B95‐D4/def2‐QZVP+COSMO‐RS(CHCl_3_)//PBEh‐3c(COSMO(CHCl_3_)) level of theory. All free energies in kcal/mol.

In contrast, the splitting of H_2_ by Cl^−^/**4 e** (**TS6**) requires 0.7 kcal/mol less energy than **TS4 b** rendering this step kinetically more favorable. The comparison of the H−H bond lengths in **TS4 b** and **TS6** indicates an early transition state for the H_2_‐activation for the Cl^−^/**4 e** FLP. In case of the simple catalytic cycle the formed Chloride ions also increasingly form Cl^−^/**4 e** (−2.5 kcal/mol) thus further disadvantaging the H_2_‐splitting via **TS4**.

In summary, we developed the FLP‐catalyzed hydrogenation of amides to yield secondary amines. The in situ formed imidoyl chloride intermediate was generated by the new developed phosphane oxide catalysis and triphosgene as oxygen scavenger. The final hydrogenation of the intermediate proceeds through two catalytic cycles as revealed by DFT‐calculations, incorporating H_2_‐activation by the imidoyl chloride and by chloride as active Lewis base. The reaction displays high functional group tolerance towards Lewis basic sites and hydrogenation susceptible groups and provides the secondary amines in high yields under mild conditions.

## Conflict of interest

The authors declare no conflict of interest.

## Supporting information

As a service to our authors and readers, this journal provides supporting information supplied by the authors. Such materials are peer reviewed and may be re‐organized for online delivery, but are not copy‐edited or typeset. Technical support issues arising from supporting information (other than missing files) should be addressed to the authors.

Supporting Information
