# Supplementary material for: Hydrogenation of Secondary Amides using Phosphane Oxide and Frustrated Lewis Pair Catalysis
Source: Chemistry. 2021 Sep 2;27(57):14179–83. doi: 10.1002/chem.202100041 (PMC8596796; doi:10.1002/chem.202100041)
Supplement: Supplementary file 1 — Supporting Information [file CHEM-27-14179-s001.pdf]

Chemistry–A European Journal

Supporting Information

Hydrogenation of Secondary Amides using Phosphane Oxide and Frustrated Lewis Pair Catalysis

Laura Köring, Nikolai A. Sitte, Markus Bursch,* Stefan Grimme, and Jan Paradies*

Table of Contents

1 General Information 4

1.1 Synthesis and Techniques 4

1.2 Reagents and Materials 4

1.3 Characterization 4

2 Borane Synthesis: Synthesis of B(2,3,6-F_3-C_6H_2)$_3$ (**4e**)[SI1b] 5

3 Phosphine Oxide Synthesis[SI3] 6

4 Substrate Synthesis 6

4.1 General Method for Synthesis of *N*-Substituted Carboxamides[SI4] 7

4.2 Synthesis of *N*-Isopropyl-4-(phenylethynyl)benzamide (**1i**)[SI5] 7

4.3 Synthesis of *N*-Benzoyl-2-aminoethyl cinnamate (**1j**)[SI6] 8

4.4 Synthesis of (S)-*N*-(1-Cyclohexyl)ethylbenzamide (**1h**) 9

5 Synthesis of Imidoyl Chloride from Tertiary *N*-Methylbenzamide 11

6 Optimization of Imidoyl Chloride Formation 12

7 FLP-Catalyzed Hydrogenations 13

7.1 Catalyst Screening for FLP-Catalyzed Hydrogenation of Imidoyl Chloride 13

7.2 Effect of Additives on Yield of the FLP-Catalyzed Hydrogenation of Imidoyl Chloride 15

7.3 General Procedure for Amide Reduction 16

7.4 Table Run Experiments 16

7.4.1 *N*-Isopropyl-(4-bromobenzyl)amine (**5a**) 16

7.4.2 *N*-Isopropylbenzylamine (**5b**) 16

7.4.3 *N*-Ethylbenzylamine (**5c**) 17

7.4.4 *N*-Methylbenzylamine (**5d**) 17

7.4.5 Dibenzylamine (**5e**) 17

7.4.6 *N*-Benzylaniline (**5f**) 18

7.4.7 *N*-Isopropyl-*N*-isobutylamine hydrochloride (**5g**) 18

7.4.8 (*S*)-*N*-Benzyl-(1-cyclohexylethyl)amine (**5h**) 18

7.4.9 *N*-Isopropyl-(4-(phenylethynyl)benzyl)amine (**5i**) 21

7.4.10 *N*-Benzyl-2-(cinnamoyloxy)ethanamine hydrochloride (**5j**) 21

7.4.11 *N*-Isopropyl-(4-methoxybenzyl)amine (**5k**) 21

7.4.12 *N*-Isopropyl-(4-nitrobenzyl)amine (**5l**) 22

7.5 NMR spectra 23

8 NMR Spectroscopic Investigation of Borane Adducts 36

8.1 $B(2,3,6\text{-}F_3\text{-}C_6H_2)_3$ (**4e**) and Amide **1a** 36

8.2 $B(2,3,6\text{-}F_3\text{-}C_6H_2)_3$ (**4e**) and Phosphine Oxides **3a-d** 37

8.3 $B(2,3,6\text{-}F_3\text{-}C_6H_2)_3$ (**4e**) and Chloride 39

9 Kinetic Investigation 40

9.1 Hydrogenation of Imidoyl Chloride **2b** With Added Ammonium Hydrochloride 40

9.2 Hydrogenation of Imidoyl Chloride **2b** Without Added Ammonium Hydrochloride 41

10 References 42

11 Computational Details 43

11.1 General remarks, geometry optimizations, energy calculations and vibrational frequency calculations 43

11.2 Solvation corrections and Gibbs free energies 44

11.3 Energy contributions 47

11.4 References for Computational Details 48

1 General Information

1.1 Synthesis and Techniques

Preparation of amides was carried out in fresh CHROMAGLOBE crimp seal glass vials. Catalytic reactions were prepared in a GLOVEBOX SYSTEMS inert atmosphere glovebox. For NMR scale experiments Teflon cap sealed J. YOUNG NMR tubes were used. For high pressure NMR scale experiments a MAN ON THE MOON Millireactor was used. Table run experiments were carried out in a stainless-steel high-pressure reactor. Deuterated solvents were degassed by 3 freeze-pump-thaw cycles and stored over 3 Å molecular sieves. Chloroform, pentane, heptane, toluene and tetrahydrofuran were bought as HPLC-Grade (≥ 99% purity) from CARL ROTH, dispensed into STRAUS flasks equipped with YOUNG-type Teflon valve stop-cocks and stored over 3 Å resp. 4 Å molecular sieves. Molecular sieves were activated at 280 °C under vacuum and stored under inert atmosphere. The following abbreviations for solvents were used: ethyl acetate (EA), cyclohexane (CH), dichloromethane (DCM), tetrahydrofuran (THF).

1.2 Reagents and Materials

All commercially available reagents were purchased from SIGMA ALDRICH, ABCR or TCI CHEMICALS and were used as received without further purification unless stated otherwise. $B(C_6F_5)_3$ (**4a**) was purchased from BOULDER SCIENTIFIC COMPANY and used as received. $B(2,3,5,6\text{-}F_4\text{-}C_6H)_3$ (**4b**), $B(2,4,6\text{-}F_3\text{-}C_6H_2)_3$ (**4c**), $B(2,6\text{-}F_2\text{-}C_6H_3)_3$ (**4d**) and $B(2,3,6\text{-}F_3\text{-}C_6H_2)_3$ (**4e**) were prepared as described earlier[SI1] or using literature methods.[SI2] Hydrogen 6.0 was provided by AIR LIQUIDE and used without further purification (for high pressure experiments) or purified through JOHNSON MATTHEY Model HIG 35XL gas purifier (for NMR scale experiments).

1.3 Characterization

^{1}H, ^{13}C, ^{31}P, ^{19}F and ^{11}B NMR spectra were recorded on a BRUKER AV 300 (300 MHz), a BRUKER AV 500 (500 MHz) or a BRUKER Ascend 700 (700 MHz) spectrometer as solutions. Chemical shifts are expressed in parts per million (ppm, δ) downfield from tetramethylsilane (TMS) and are referenced to the residual solvent signal of $CDCl_3$ (7.26 ppm for ^{1}H NMR, 77.16 ppm for ^{13}C NMR) or CD_2Cl_2 (5.32 ppm for ^{1}H NMR, 53.84 ppm for ^{13}C NMR). ^{11}B NMR and ^{19}F NMR spectra are referenced to $BF_3{\cdot}OEt_2$ and $CFCl_3$, respectively. All coupling constants (*J*) are absolute values and are expressed in Hertz (Hz). The spectra were analyzed according to first order and the descriptions of the signals include: s = singlet, d = doublet, t = triplet, q = quartet, m = multiplet, dd = doublet of doublets, etc.

Exact assignment of signals was done under consideration of $^{1}H,^{1}H$-COSY, $^{1}H,^{13}C$-HSQC-, $^{1}H,^{13}C$-HMBC-, and DEPT135-spectra. The following abbreviations were used: CH_3 = primary (RCH_3), CH_2 = secondary (R_2CH_2), CH = tertiary (R_3CH), Cq = quaternary (R_4C), H_{Ar} = aromatic hydrogen. All mass spectra were recorded on a WATERS Synapt 2G spectrometer (electrospray ionization, ESI). For analytical HPLC at chiral stationary phase for the determination of enantiomeric excess (*ee*) a VARIAN 920-LC was used with the following capillary column: CHIRALCEL IA.

2 Borane Synthesis: Synthesis of $B(2,3,6\text{-}F_3\text{-}C_6H_2)_3$ (4e)[SI1b]

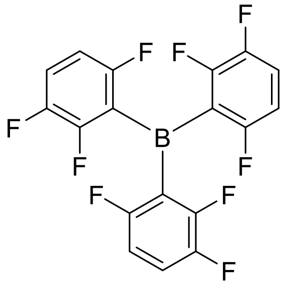

In a SCHLENK flask under argon atmosphere 2-Bromo-1,3,4-trifluorobenzene (2.11 g, 9.99 mmol, 3.00 equiv.) was dissolved in abs. THF (10 mL). The solution was cooled to -20 °C, and a solution of *i*PrMgCl in THF (2 M, 9.99 mmol, 3.00 equiv.) was added slowly. The mixture was stirred at -20 °C for 1 h and then at room temperature for additional 3 h. The Grignard solution was added slowly via cannula to a solution of $BF_3\cdot OEt_2$ (473 mg, 3.33 mmol, 1.00 equiv.) in abs. toluene (15 mL) at -20 °C. The mixture was left in the cooling bath to slowly reach room temperature and was stirred overnight. The solvent was evaporated, the residue was dried under vacuum and was extracted with abs. toluene. After the solvent was evaporated, the borane-THF adduct was suspended in abs. *n*-pentane (5 mL), chlorodimethylsilane (1 mL) was added, and the suspension was stirred overnight. All volatiles were removed under vacuum, and the residue was washed with small amounts of abs. *n*-pentane. After drying, the product was obtained as a white powder. Further purification was achieved by extraction with hot abs. heptane. Yield: 40% (540 mg, 1.34 mmol).

^{1}H-NMR (500 MHz, 303 K, $CDCl_3$) δ = 7.32 (qd, *J* = 9.2 Hz, *J* = 5.3 Hz, 3H), 6.86 – 6.82 (m, 3H); **^{11}B-NMR** (160 MHz, 303 K, $CDCl_3$) δ = 61.5; **^{19}F-NMR** (282 MHz, 298 K, $CDCl_3$) δ = -104.2 – -104.3 (m), -123.4 – -123.5 (m), -142.7 – -142.9 (m).

The NMR data is in good agreement with the reported chemical shifts and signal pattern.[SI1b]

3 Phosphine Oxide Synthesis[SI3]

The phosphine (0.3 mmol, 1.0 equiv.) was dissolved in THF and a hydrogen peroxide solution in H_2O (30%, 1.5 mmol, 5.0 equiv.) was added dropwise. The reaction mixture was stirred at room temperature for 2 h. The solvent was removed under reduced pressure, the residue was dissolved in ethyl acetate and washed twice with water. After drying over magnesium sulfate and removing the solvent, the phosphine oxide was dried under reduced pressure.

O=P(1-Naphth)$_3$ (3d)

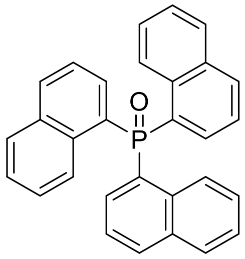

The product was obtained as a white solid. Yield: 90% (116 mg, 0.27 mmol).

^{1}H-NMR (500 MHz, 303 K, CD_2Cl_2) δ = 8.90 (m, 3H, H_{Ar}), 8.07 (m, 3H, H_{Ar}), 7.98 (m, 3H, H_{Ar}), 7.57 – 7.54 (m, 3H, H_{Ar}), 7.49 – 7.45 (m, 3H, H_{Ar}), 7.32 – 7.29 (m, 3H, H_{Ar}), 7.25 – 7.21 (m, 3H, H_{Ar}); **^{13}C-NMR** (126 MHz, 303 K, CD_2Cl_2) δ = 134.7 (d, J_{CP} = 7.3 Hz, Cq), 134.6 (d, J_{CP} = 8.2 Hz, Cq), 134.1 (d, J_{CP} = 11.9 Hz, CH), 133.7 (d, J_{CP} = 2.8 Hz, CH), 129.5 (d, $^1J_{CP}$ = 101.3 Hz, Cq), 129.3 (CH), 128.4 (d, J_{CP} = 4.6 Hz, CH), 127.6 (CH), 126.9 (CH), 124.9 (d, J_{CP} = 14.7 Hz, CH); **^{31}P{^{1}H}-NMR** (202 MHz, 303 K, CD_2Cl_2) δ = 39.6; **HRMS** ($C_{30}H_{22}OP^+$): calc *m/z* 429.1408, found 429.1406.

The phosphine oxides O=PPh_3 (**3a**), O=P(2,6-F_2-C_6H_3)$_3$ (**3b**) and O=P*t*Bu_3 (**3c**) were prepared in our working group in a similar manner.

4 Substrate Synthesis

N-Methylbenzamide (**1d**) and *N*-Phenylbenzamide (**1f**) were commercially available and used in further reactions without purification. *N*-Isopropyl-4-bromobenzamide (**1a**), *N*-Isopropylbenzamide (**1b**), *N*-Ethylbenzamide (**1c**), *N*-Benzylbenzamide (**1e**), *N*-Isopropylisobutyramide (**1g**), *N*-(1-Cyclohexyl)ethylbenzamide (**1h**), *N*-Isopropyl-4-methoxybenzamide (**1k**) and *N*-Isopropyl-4-nitrobenzamide (**1l**) were prepared by the general method. NMR and mass spectral data were in good agreement with the literature.

4.1 General Method for Synthesis of *N*-Substituted Carboxamides[SI4]

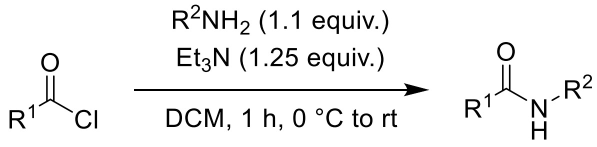

The primary amine (11.0 mmol, 1.10 equiv.) and triethylamine (12.5 mmol, 1.25 equiv.) were dissolved in DCM (12 mL, 0.83 M) and placed in a 20 mL crimp seal glass vial. The reaction mixture was cooled in an ice/water bath and the acyl chloride (10.0 mmol, 1.00 equiv.) was added dropwise via syringe under vigorous stirring. During the addition, a white precipitate was formed. After addition was completed, the reaction mixture was warmed to room temperature and stirred for 30 minutes. The reaction mixture was then diluted with DCM, until the precipitate was dissolved, and washed with aqueous HCl (1 M). The aqueous phase was extracted one more time with a small amount of DCM, and the combined organic phases were dried over $MgSO_4$. The volatiles were removed under reduced pressure, yielding the crude product. Purification was accomplished by column chromatography (silica, mixtures of CH and EA).

4.2 Synthesis of *N*-Isopropyl-4-(phenylethynyl)benzamide (1i)[SI5]

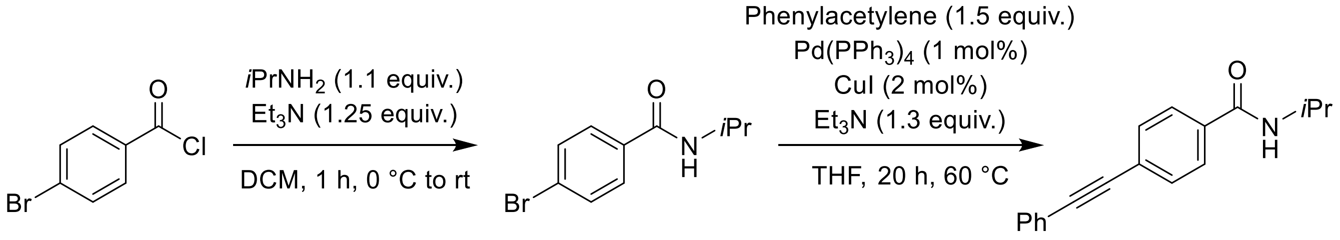

N-Isopropyl-4-bromobenzamide (**1a**) was prepared according to the general procedure and was used for the next step without further purification. Tetrakis(triphenylphosphine)palladium (116 mg, 100 µmol, 1.00 mol%) and copper(I) iodide (38.0 mg, 200 µmol, 2.00 mol%) were placed in a SCHLENK flask under argon atmosphere. The addition of abs. THF (10 mL) resulted in a yellow suspension. Triethylamine (1.80 mL, 13.0 mmol, 1.30 equiv.), phenylacetylene (1.65 mL, 15.0 mmol, 1.50 equiv.) and finally a solution of *N*-Isopropyl-4-bromobenzamide (2.42 g, 10.0 mmol, 1.00 equiv.) in abs. THF (10 mL) were added. The dark suspension was stirred at 60 °C for 20 h. The reaction mixture was filtered through a pad of CELITE® and rinsed with DCM. The brown clear solution was poured on sat. aqueous NH_4Cl and extracted twice with DCM. The combined organic phases were washed with sat. aqueous NaCl and then dried over $MgSO_4$. After removing the volatiles under reduced pressure, the crude product was purified by column chromatography (silica, CH/EA 5/1). Further purification was

achieved by recrystallization from ethyl acetate. The product was obtained as a white solid. Yield: 29% (770 mg, 2.92 mmol).

^{1}H-NMR (700 MHz, 298 K, $CDCl_3$) δ = 7.74 (d, $^3J_{HH}$ = 8.3 Hz, 2H, H_{Ar}), 7.57 (d, $^3J_{HH}$ = 8.3 Hz, 2H, H_{Ar}), 7.55 – 7.53 (m, 2H, H_{Ar}), 7.37 – 7.35 (m, 3H, H_{Ar}), 5.95 (br, 1H, NH), 4.29 (dsept, $^3J_{HH}$ = 7.5 Hz, $^3J_{HH}$ = 6.6 Hz, 1H, NC*H*(CH_3)$_2$), 1.27 (d, $^3J_{HH}$ = 6.6 Hz, 6H, NCH(C*H*$_3$)$_2$); **^{13}C-NMR** (176 MHz, 298 K, $CDCl_3$) δ = 166.1 (Cq), 134.4 (Cq), 131.8 (CH), 131.8 (CH), 128.8 (CH), 128.6 (CH), 127.0 (CH), 126.5 (Cq), 122.9 (Cq), 91.7 (Cq), 88.7 (Cq), 42.2 (CH), 23.0 (CH_3); **HRMS** ($C_{18}H_{18}NO^+$): calc *m/z* 264.1388, found 264.1388.

4.3 Synthesis of *N*-Benzoyl-2-aminoethyl cinnamate (1j)[SI6]

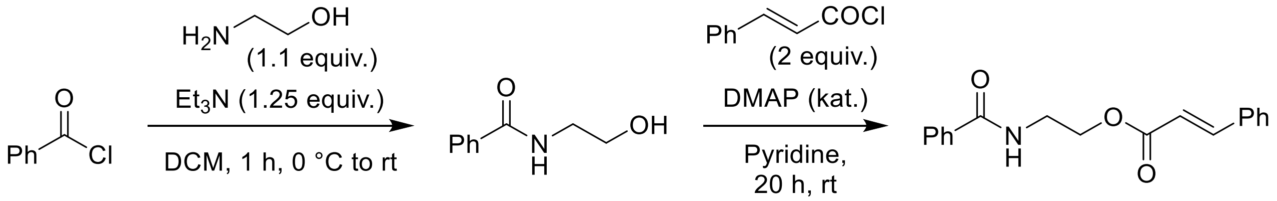

N-(2-Hydroxyethyl)benzamide was prepared according to the general procedure starting from 2-aminoethanol and benzoyl chloride and was used for the next step without further purification. *N*-(2-Hydroxyethyl)benzamide (1.08 g, 6.54 mmol, 1.00 equiv.) was dissolved in pyridine (8.17 mL, 0.8 M). A small amount of 4-dimethylaminopyridine (DMAP) was added, followed by cinnamic acid chloride (2.18 g, 13.1 mmol, 2.00 equiv.). The resulting suspension was stirred at room temperature. After full conversion (TCL monitoring, 20 h) aqueous HCl (1 M) was added. The reaction mixture was extracted three times with ethyl acetate, and the combined organic phases were washed with aqueous HCl (1 M), sat. aqueous Na_2CO_3 and water. After drying over $MgSO_4$ the volatiles were removed under reduced pressure. The crude product was purified by column chromatography (silica, CH/EA 2/1), which yielded the product as a light brown solid. Yield: 53% (1.56 g, 5.29 mmol).

^{1}H-NMR (500 MHz, 303 K, $CDCl_3$) δ = 7.81 – 7.79 (m, 2H, H_{Ar}), 7.73 (d, $^3J_{HH}$ = 16.0 Hz, 1H, PhC*H*=CH), 7.54 – 7.49 (m, 3H, H_{Ar}), 7.46 – 7.42 (m, 2H, H_{Ar}), 7.41 – 7.38 (m, 3H, H_{Ar}), 6.66 (br, 1H, NH), 6.46 (d, $^3J_{HH}$ = 16.0 Hz, 1H, PhCH=C*H*), 4.45 (t, $^3J_{HH}$ = 5.2 Hz, 2H, OCH_2), 3.81 (dt, $^3J_{HH}$ = 5.4 Hz, $^3J_{HH}$ = 5.3 Hz, 2H, OCH_2C*H*$_2$); **^{13}C-NMR** (126 MHz, 303 K, $CDCl_3$) δ = 167.7 (Cq), 167.5 (Cq), 145.9 (CH), 134.4 (Cq), 134.3 (Cq), 131.7 (CH), 130.7 (CH), 129.1 (CH), 128.8 (CH), 128.3 (CH), 127.1 (CH), 117.5 (CH), 63.6 (CH_2), 40.0 (CH_2) ; **HRMS** ($C_{18}H_{17}NO_3Na^+$): calc *m/z* 318.1106, found 318.1093.

4.4 Synthesis of (S)-*N*-(1-Cyclohexyl)ethylbenzamide (1h)

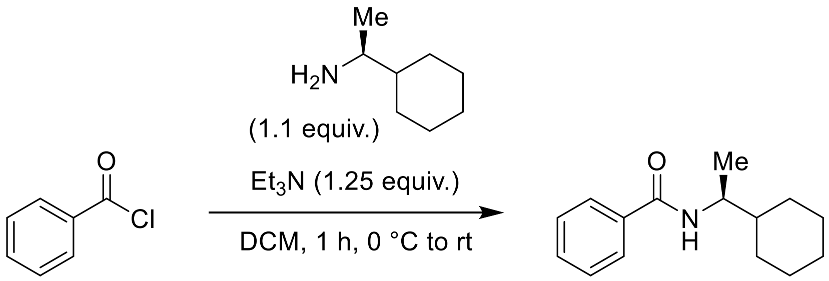

The secondary carboxamide was synthesized according to the general procedure starting from (*S*)-1-cyclohexylethylamine and benzoyl chloride. Purification was accomplished by column chromatography (DCM/EA 100/1). Yield: 95%. (2.20 g, 9.51 mmol). *ee* = 96%.

HPLC (IA, 15 °C, heptane/*i*PrOH 85/15, flow rate: 0.7 mL/min, 260 nm): t_R = 8.9 min (*R*), t_R = 10.2 min (*S*).

^{1}H-NMR (700 MHz, 298 K, $CDCl_3$) δ = 7.76 – 7.74 (m, 2H, H_{Ar}), 7.49 – 7.47 (m, 1H, H_{Ar}), 7.43 – 7.41 (m, 2H, H_{Ar}), 5.97 (br, 1H, NH), 4.08 (ddq, $^3J_{HH}$ = 8.9 Hz, $^3J_{HH}$ = 6.7 Hz, $^3J_{HH}$ = 6.7 Hz, 1H, NCH), 1.83 – 1.79 (m, 1H, CH_2), 1.78 – 1.74 (m, 3H, CH_2), 1.68 – 1.65 (m, 1H, CH_2), 1.46 – 1.41 (m, 1H, NCHC*H*), 1.27 – 1.20 (m, 2H, CH_2), 1.18 (d, $^3J_{HH}$ = 6.8 Hz, 3H, NCHCH_3), 1.15 – 1.00 (m, 3H, CH_2); **^{13}C-NMR** (176 MHz, 298 K, $CDCl_3$) δ = 166.9 (Cq), 135.3 (Cq), 131.4 (CH), 128.7 (CH), 126.9 (CH), 50.0 (CH), 43.4 (CH), 29.3 (CH_2), 29.3 (CH_2), 26.5 (CH_2), 26.3 (CH_2), 26.3 (CH_2), 18.1 (CH_3) ; **HRMS** ($C_{15}H_{21}NONa^+$): calc *m*/*z* 254.1521, found 254.1504.

HPLC results:

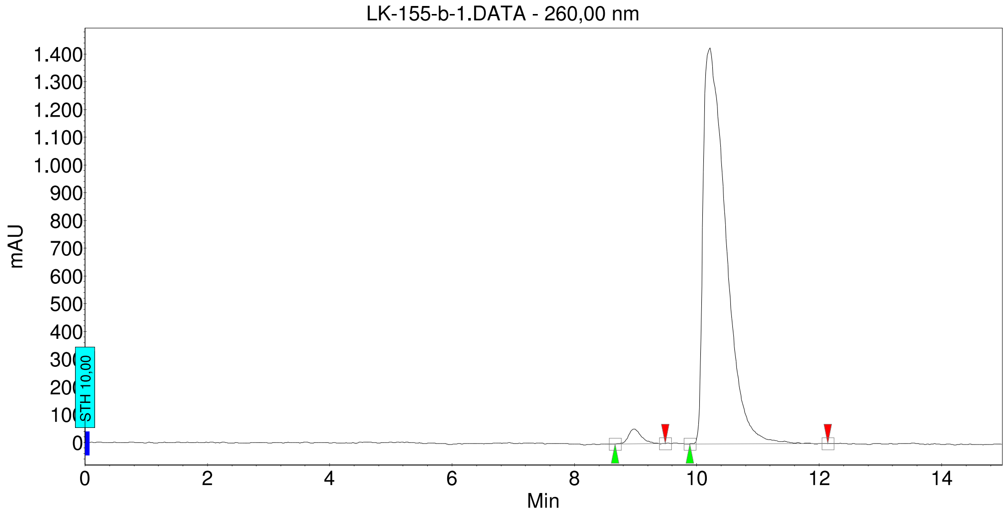

Peak results :

Index	Name	Time [Min]	Quantity [% Area]	Height [mAU]	Area [mAU.Min]	Area % [%]
1	UNKNOWN	8,97	2,09	53,2	13,0	2,086
2	UNKNOWN	10,21	97,91	1426,4	610,2	97,914
Total			100,00	1479,6	623,2	100,000

racemic sample:

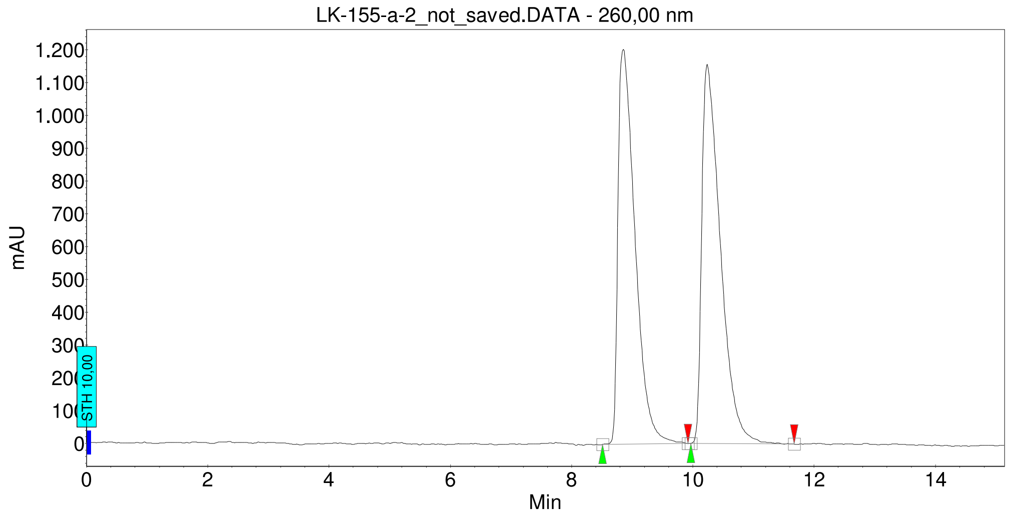

Peak results :

Index	Name	Time [Min]	Quantity [% Area]	Height [mAU]	Area [mAU.Min]	Area % [%]
1	UNKNOWN	8,85	48,64	1203,5	391,3	48,644
2	UNKNOWN	10,24	51,36	1155,7	413,2	51,356
Total			100,00	2359,3	804,5	100,000

5 Synthesis of Imidoyl Chloride from Tertiary *N*-Methylbenzamide

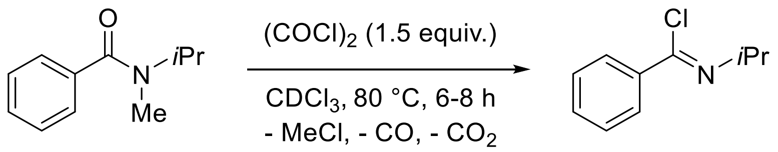

N-Isopropylbenzimidoyl chloride (**2b**) was synthesized by heating a solution of *N*-Isopropyl-*N*-methylbenzamide (17.7 mg, 100 µmol, 1.00 equiv.) with oxalyl chloride (19.0 mg, 150 µmol, 1.50 equiv.) in $CDCl_3$ (0.6 mL, 0.17 M) to 80 °C for several hours until full conversion was achieved. The process was monitored by ^{1}H-NMR spectroscopy. The volatiles were removed afterwards, and the product was used without further purification for the catalyst screening (see section 7.1).

^{1}H-NMR (300 MHz, 303 K, $CDCl_3$) δ = 8.04 – 7.95 (m, 2H, H_{Ar}), 7.50 – 7.35 (m, 3H, H_{Ar}), 4.17 (sept, $^3J_{HH}$ = 6.3 Hz, 1H, NCH), 1.29 (d, $^3J_{HH}$ = 6.3 Hz, 6H, NCH(CH_3)$_2$).

6 Optimization of Imidoyl Chloride Formation

In a glovebox, *N*-Isopropyl-4-bromobenzamide (**1a**) (12.1 mg, 50.0 µmol, 1.00 equiv.), the reagent (20 µmol – 500 µmol, 0.4 equiv. – 10 equiv.) and, if necessary, the catalyst (10 µmol, 20 mol%) were dissolved in 0.6 mL $CDCl_3$ and transferred to a J. YOUNG NMR tube. The sample was heated on a shaking plate to 70 °C or 90 °C, and the process was monitored by ^{1}H-NMR spectroscopy.

Table S1: Optimization of Imidoyl Chloride Formation.

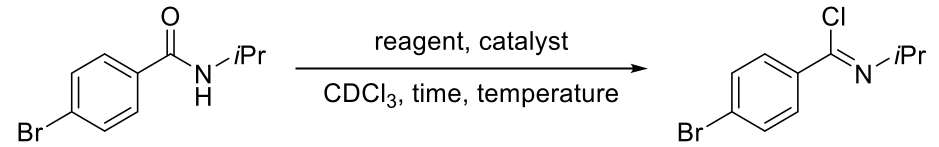

entry	reagent (equiv.)	catalyst (mol%)	temperature [°C]	time [h]	yield[a] [%]
1	$SOCl_2$ (1.5)	-	70	18	30
2	$SOCl_2$ (10)	-	70	3	>98
3	$(COCl)_2$ (1.1)	-	70	18	0[b]
4	$(COCl)_2$ (1.0)	$O{=}PPh_3$ (**3a**) (100)	70	110	93
5	$(COCl)_2$ (1.1)	$O{=}PPh_3$ (**3a**) (20)	70	18	20[c]
6	$CO(OCCl_3)_2$ (0.4)	-	70	18	<20
7	$CO(OCCl_3)_2$ (0.4)	$O{=}PPh_3$ (**3a**) (20)	70	18	90
8	$CO(OCCl_3)_2$ (0.4)	$O{=}PPh_3$ (**3a**) (20)	70	34	>98
9	$CO(OCCl_3)_2$ (0.4)	$O{=}PPh_3$ (**3a**) (20)	90	18	>98
10	$CO(OCCl_3)_2$ (0.4)	$O{=}P(2,6\text{-}F_2\text{-}C_6H_3)_3$ (**3b**) (20)	70	18	80
11	$CO(OCCl_3)_2$ (0.4)	$O{=}P(2,6\text{-}F_2\text{-}C_6H_3)_3$ (**3b**) (20)	90	18	90
12	$CO(OCCl_3)_2$ (0.4)	$O{=}P\textit{t}Bu_3$ (**3c**) (20)	70	18	<30
13	$CO(OCCl_3)_2$ (0.4)	$O{=}P\textit{t}Bu_3$ (**3c**) (20)	90	18	55
14	$CO(OCCl_3)_2$ (0.4)	$O{=}P(1\text{-Naphth})_3$ (**3d**) (20)	70	18	60
15	$CO(OCCl_3)_2$ (0.4)	$O{=}P(1\text{-Naphth})_3$ (**3d**) (20)	70	42	85
16	$CO(OCCl_3)_2$ (0.4)	$O{=}P(1\text{-Naphth})_3$ (**3d**) (20)	90	18	>98

a) determined by ^{1}H-NMR spectroscopy; for yield calculation, signals of all species were integrated and compared, b) contaminated with >98% of acylation product, c) contaminated with 5% amide and 75% of acylation product.

7 FLP-Catalyzed Hydrogenations

7.1 Catalyst Screening for FLP-Catalyzed Hydrogenation of Imidoyl Chloride

4 bar H_2:

In a glovebox, a borane catalyst (20 µmol, 20 mol%) and *N*-Isopropylbenzimidoyl chloride (**2b**) (100 µmol, 1.00 equiv., synthesized according to section 5) were dissolved in 0.6 mL $CDCl_3$ and transferred to a J. Young NMR tube with Teflon tap. The sample was then frozen in liquid nitrogen, the headspace was evacuated, and the sample was charged with hydrogen at -196 °C. After sealing and thawing, the hydrogen pressure inside the sample reached approximately 4 bar. The sample was then heated on a shaking plate to ensure hydrogen exchange. After the given time (see Table S2) the NMR tube was cooled to room temperature, and the crude reaction mixture was analyzed by ^{1}H-NMR spectroscopy.

80 bar H_2:

In a glovebox, $B(2,3,6\text{-}F_3\text{-}C_6H_2)_3$ (2.0 µmol, 2.0 mol%) and *N*-Isopropylbenzimidoyl chloride (**2b**) (100 µmol, 1.00 equiv.) were dissolved in 0.6 mL $CDCl_3$. The sample was transferred to a Millireactor, charged with 80 bar hydrogen, and heated in an oil bath. After the given time (see Table S2), the crude reaction mixture was transferred to an NMR tube and analyzed by ^{1}H-NMR spectroscopy.

Table S2: FLP-catalyzed hydrogenation of imidoyl chloride.

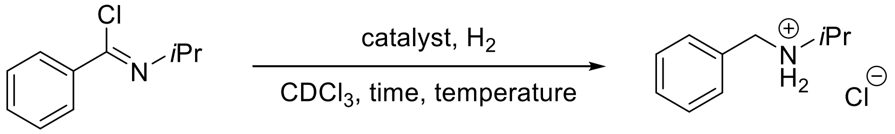

entry	catalyst	loading [mol%]	LA[a] [%]	H_2 [bar]	temperature [°C]	time [h]	yield[b] [%]
1	$B(C_6F_5)_3$ (**4a**)	20	100	4	70	90	3
2	$B(2,3,5,6\text{-}F_4\text{-}C_6H)_3$ (**4b**)	20	98	4	70	90	0
3	$B(2,4,6\text{-}F_3\text{-}C_6H_2)_3$ (**4c**)	20	87	4	70	90	53
4	$B(2,6\text{-}F_2\text{-}C_6H_3)_3$ (**4d**)	20	82	4	70	90	12
5	$B(2,3,6\text{-}F_3\text{-}C_6H_2)_3$ (**4e**)	20	92	4	70	90	99
6	$B(2,3,6\text{-}F_3\text{-}C_6H_2)_3$ (**4e**)	5	92	80	70	20	99

a) according to Gutmann-Beckett with $B(C_6F_5)_3$ referenced to 100% Lewis acidity, b) determined by ^{1}H-NMR spectroscopy; for yield calculation, signals of starting material before the reaction and of the product after the reaction were integrated and normalized against the silicone grease signal as an internal standard (example given below).

calculation example (Table S2, entry 3):

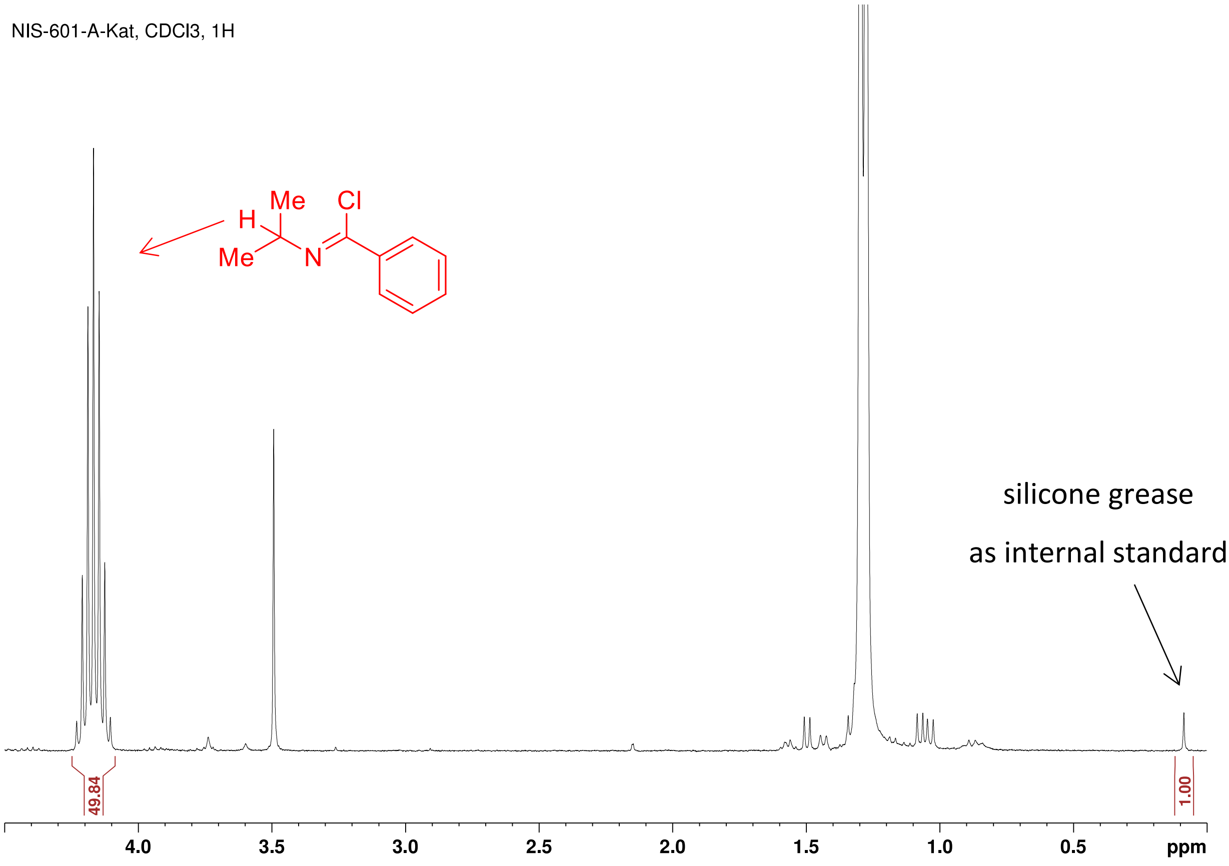

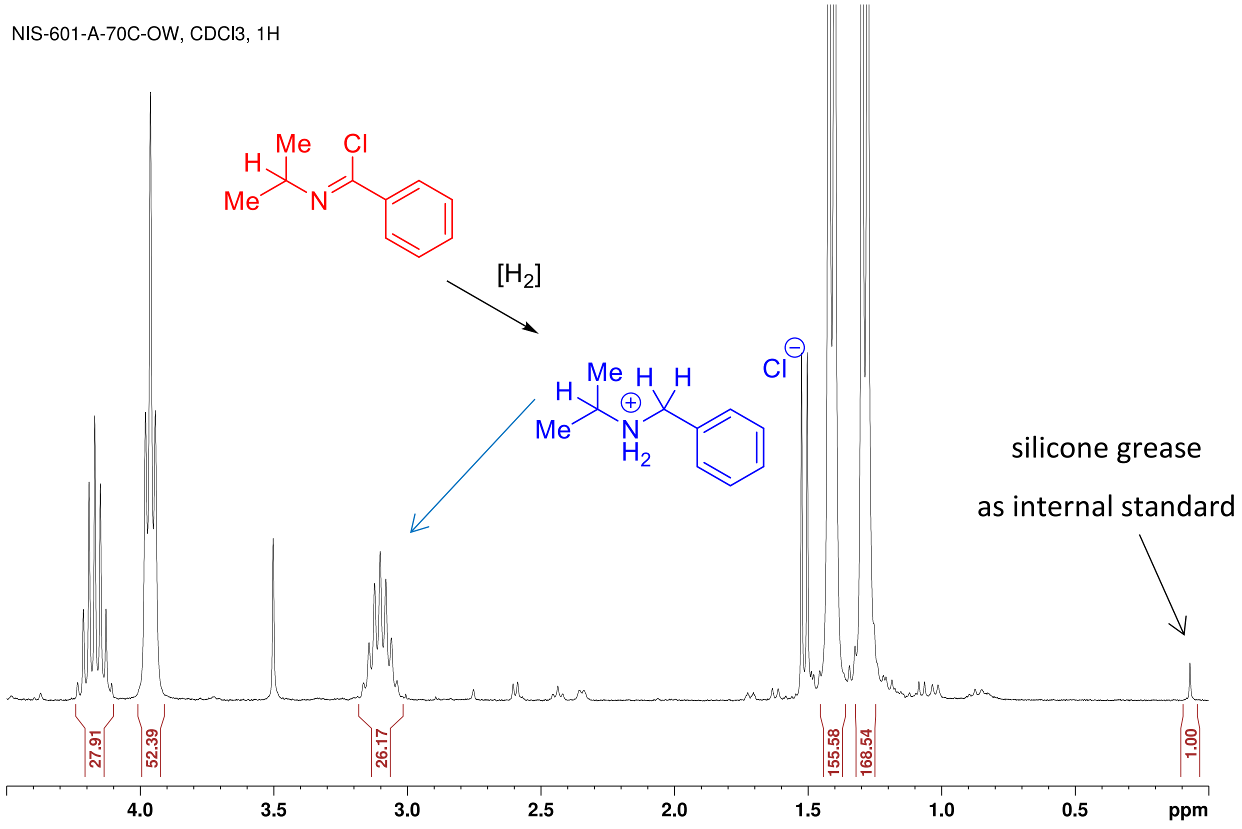

Yield = Proton integral product at end / Proton integral starting material at start

= 26.17 / 49.84 = 52.5% ≈ 53%

7.2 Effect of Additives on Yield of the FLP-Catalyzed Hydrogenation of Imidoyl Chloride

In a glovebox, the imidoyl chloride **2a** (13.0 mg, 50.0 µmol, 1.00 equiv.), $B(2,3,6\text{-}F_3\text{-}C_6H_2)_3$ (4.0 mg, 10 µmol, 20 mol%) and an additive (10 µmol, 0.2 equiv.) were dissolved in 0.6 mL $CDCl_3$ and transferred to a J. YOUNG NMR tube with Teflon tap. The sample was then frozen in liquid nitrogen, the headspace was evacuated, and the sample was charged with hydrogen at -196 °C. After sealing and thawing, the hydrogen pressure inside the sample reached approximately 4 bar. The sample was then heated on a shaking plate to ensure hydrogen exchange. After 20 h, the crude reaction mixture was analyzed by ^{1}H-NMR spectroscopy. The effect of phosphine oxides, activation agents or amide on the FLP-catalyzed hydrogenation was shown. The phosphine oxides **3c** and **3d** had no interference with the borane in the FLP-catalyzed hydrogenation (see Table S3), but the conversion of amide to imidoyl chloride is more efficient with the phosphine oxide **3d**.

Table S3: Effect of phosphine oxide, activation agent or amide on FLP-catalyzed hydrogenation of imidoyl chloride.

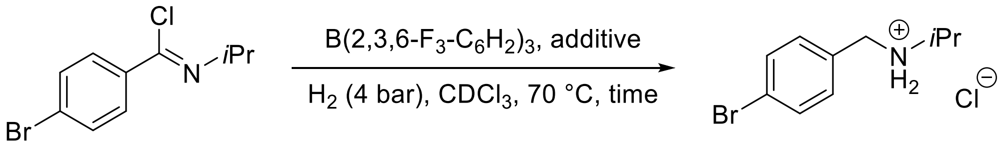

entry	catalyst loading [mol%]	additive	time [h]	yield[a] [%]
1	20	-	20	>98
2	20	$O{=}PPh_3$ (**3a**) (0.2 equiv.)	20	0
3	20	$O{=}P(2,6\text{-}F_2\text{-}C_6H_3)_3$ (**3b**) (0.2 equiv.)	20	10
4	20	$O{=}P\textit{t}Bu_3$ (**3c**) (0.2 equiv.)	20	>98
5	20	$O{=}P(1\text{-Naphth})_3$ (**3d**) (0.2 equiv.)	20	>98
6	20	$SOCl_2$ (0.2 equiv.)	20	50
7	20	$CO(OCCl_3)_2$ (0.2 equiv.)	20	80
8	20	amide **1a** (0.2 equiv.)	20	35

a) determined by ^{1}H-NMR spectroscopy; for yield calculation, signals of all species were integrated and compared.

7.3 General Procedure for Amide Reduction

In a glovebox, a J. YOUNG NMR tube was charged with the *N*-substituted carboxamide substrate **1** (500 µmol, 1.00 equiv.), O=P(1-Naphth)$_3$ (**3d**) (42.8 mg, 100 µmol, 20.0 mol%) and triphosgene (50.4 mg, 170 µmol, 0.34 equiv.). The mixture was dissolved in 0.6 mL $CDCl_3$ and heated to 70 °C or 90 °C on a shaking plate. The reaction was monitored by ^{1}H-NMR spectroscopy. After full conversion to the imidoyl chloride, the reaction mixture was transferred to a vial with B(2,3,6-F$_3$-C$_6$H$_2$)$_3$ (**4e**) (10.1 mg, 25.0 µmol, 5.00 mol% or 20.2 mg, 50.0 µmol, 10.0 mol%) and was diluted with 2.4 mL abs. $CHCl_3$. The sample was charged with hydrogen (80 bar), and the stainless-steel high-pressure reactor was then heated to 70 °C or 90 °C in an oil bath. After 20 h or 40 h, the reactor was cooled to room temperature and depressurized. The product mixture was diluted with DCM and washed with aq. ammonia and sat. aq. Na_2CO_3. The phases were separated, and the aqueous phase was extracted three times with DCM. The combined organic phases were loaded with a small portion of silica and evaporated to dryness. After column chromatography, the amine was analyzed by NMR spectroscopy and mass spectrometry.

7.4 Table Run Experiments

7.4.1 *N*-Isopropyl-(4-bromobenzyl)amine (**5a**)

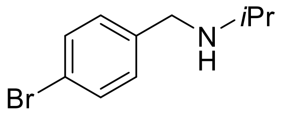

According to the general procedure 7.3, the activation was performed at 90 °C for 5 h, and the reduction was performed with 5 mol% B(2,3,6-F$_3$-C$_6$H$_2$)$_3$ at 90 °C for 20 h; CH/EA 1/1; colorless oil, 81% (92.0 mg, 403 µmol).

^{1}H-NMR (500 MHz, 303 K, $CDCl_3$) δ = 7.44 – 7.41 (m, 2H, H$_{Ar}$), 7.22 – 7.19 (m, 2H, H$_{Ar}$), 3.73 (s, 2H, NCH$_2$), 2.83 (sept, $^3J_{HH}$ = 6.2 Hz, 1H, NCH), 1.46 (br, 1H, NH), 1.09 (d, $^3J_{HH}$ = 6.2 Hz, 6H, NCH(C*H*$_3$)$_2$); **^{13}C-NMR** (126 MHz, 303 K, $CDCl_3$) δ = 139.9 (Cq), 131.6 (CH), 130.0 (CH), 120.7 (Cq), 51.0 (CH$_2$), 48.3 (NCH), 23.0 (CH$_3$); **HRMS** ($C_{10}H_{15}NBr^+$): calc. *m/z* 228.0388, found 228.0386.

7.4.2 *N*-Isopropylbenzylamine (**5b**)

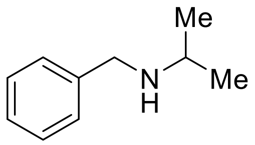

According to the general procedure 7.3, the activation was performed at 70 °C for 5 h, and the reduction was performed with 5 mol% B(2,3,6-F$_3$-C$_6$H$_2$)$_3$ at 70 °C for 20 h; DCM/EA 20/1 then EA; yellow oil; 76% (56.9 mg, 381 µmol).

^{1}H-NMR (500 MHz, 303 K, $CDCl_3$) δ = 7.32 – 7.31 (m, 4H, H_{Ar}), 7.26 – 7.22 (m, 1H, H_{Ar}), 3.79 (s, 2H, $NC\mathit{H}_2Ph$), 2.86 (sept, $^{3}J_{HH}$ = 6.2 Hz, 1H, NCH), 1.37 (br, 1H, NH), 1.10 (d, $^{3}J_{HH}$ = 6.2 Hz, 6H, $NCH(C\mathit{H}_3)_2$); **^{13}C-NMR** (126 MHz, 303 K, $CDCl_3$) δ = 140.9 (Cq), 128.6 (CH), 128.3 (CH), 127.0 (CH), 51.8 (CH_2), 48.3 (NCH), 23.1 (CH_3); **HRMS** ($C_{10}H_{16}N^+$): calc. *m/z* 150.1283, found 150.1271.

7.4.3 *N*-Ethylbenzylamine (**5c**)

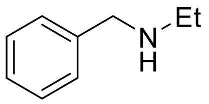

According to the general procedure 7.3, the activation was performed at 90 °C for 3 h, and the reduction was performed with 5 mol% B(2,3,6-F_3-C_6H_2)$_3$ at 90 °C for 20 h; DCM/EA 20/1 then EA; yellow oil; 62% (42.2 mg, 312 µmol).

^{1}H-NMR (500 MHz, 303 K, $CDCl_3$) δ = 7.33 – 7.31 (m, 4H, H_{Ar}), 7.25 – 7.23 (m, 1H, H_{Ar}), 3.80 (s, 2H, NCH_2Ph), 2.69 (q, $^{3}J_{HH}$ = 7.1 Hz, 2H, $NC\mathit{H}_2CH_3$), 1.42 (br, 1H, NH), 1.14 (t, $^{3}J_{HH}$ = 7.1 Hz, 3H, $NCH_2C\mathit{H}_3$); **^{13}C-NMR** (126 MHz, 303 K, $CDCl_3$) δ = 140.7 (Cq), 128.5 (CH), 128.3 (CH), 127.0 (CH), 54.1 ($PhCH_2$), 43.8 ($N\mathit{C}H_2CH_3$), 15.4 (CH_3); **HRMS** ($C_9H_{14}N^+$): calc. *m/z* 136.1126, found 136.1116.

7.4.4 *N*-Methylbenzylamine (**5d**)

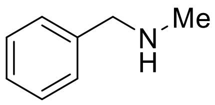

According to the general procedure 7.3, the activation was performed at 90 °C for 3 h, and the reduction was performed with 5 mol% B(2,3,6-F_3-C_6H_2)$_3$ at 90 °C for 20 h; DCM/EA 20/1 then EA; yellow oil; 71% (42.8 mg, 353 µmol).

^{1}H-NMR (700 MHz, 298 K, $CDCl_3$) δ = 7.35 – 7.30 (m, 4H, H_{Ar}), 7.27 – 7.24 (m, 1H, H_{Ar}), 3.75 (s, 2H, NCH_2Ph), 2.46 (s, 3H, NCH_3), 1.78 (br, 1H, NH); **^{13}C-NMR** (176 MHz, 298 K, $CDCl_3$) δ = 140.1 (Cq), 128.5 (CH), 128.4 (CH), 127.2 (CH), 56.2 ($PhCH_2$), 36.1 (NCH_3); **HRMS** ($C_8H_{12}N^+$): calc. *m/z* 122.0970, found 122.0970.

7.4.5 Dibenzylamine (**5e**)

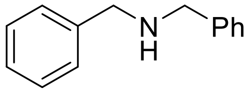

According to the general procedure 7.3, the activation was performed at 90 °C for 5 h, and the reduction was performed with 5 mol% B(2,3,6-F_3-C_6H_2)$_3$ at 90 °C for 20 h; DCM/EA 50/1 then DCM/EA 5/1; yellow oil; 64% (63.1 mg, 320 µmol).

^{1}H-NMR (700 MHz, 298 K, $CDCl_3$) δ = 7.35 – 7.32 (m, 8H, H_{Ar}), 7.27 – 7.26 (m, 1H, H_{Ar}), 7.26 – 7.25 (m, 1H, H_{Ar}), 3.82 (s, 4H, NCH_2Ph), 1.77 (br, 1H, NH); **^{13}C-NMR** (176 MHz, 298 K, $CDCl_3$) δ = 140.3 (Cq),

128.6 (CH), 128.3 (CH), 127.1 (CH), 53.3 ($PhCH_2$); **HRMS** ($C_{14}H_{16}N^+$): calc. *m/z* 198.1283, found 198.1267.

7.4.6 *N*-Benzylaniline (**5f**)

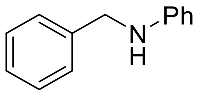

According to the general procedure 7.3, the activation was performed at 90 °C for 15 h with the addition of 2,6-Lutidine (53.6 mg, 500 µmol, 1.00 equiv.), and the reduction was performed with 10 mol% B(2,3,6-F_3-C_6H_2)$_3$ at 90 °C for 20 h; CH/EA 100/1; light brown solid; 61% (55.9 mg, 305 µmol).

^{1}H-NMR (700 MHz, 298 K, $CDCl_3$) δ = 7.39 – 7.38 (m, 2H, H_{Ar}), 7.36 – 7.34 (m, 2H, H_{Ar}), 7.30 – 7.27 (m, 1H, H_{Ar}), 7.20 – 7.17 (m, 2H, H_{Ar}), 6.74 – 6.72 (m, 1H, H_{Ar}), 6.66 – 6.64 (m, 2H, H_{Ar}), 4.34 (s, 2H, NCH_2Ph), 4.03 (br, 1H, NH); **^{13}C-NMR** (176 MHz, 298 K, $CDCl_3$) δ = 148.3 (C_q), 139.6 (C_q), 129.4 (CH), 128.8 (CH), 127.7 (CH), 127.4 (CH), 117.7 (CH), 113.0 (CH), 48.7 ($PhCH_2$); **HRMS** ($C_{13}H_{14}N^+$): calc. *m/z* 184.1126, found 184.1115.

7.4.7 *N*-Isopropyl-*N*-isobutylamine hydrochloride (**5g**)

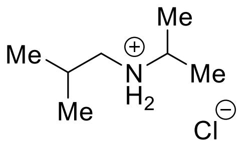

According to the general procedure 7.3, the activation was performed at 90 °C for 30 min, and the reduction was performed with 5 mol% B(2,3,6-F_3-C_6H_2)$_3$ at 90 °C for 20 h; DCM/EA 20/1 then EA, the collected fractions were treated with HCl in Et_2O (0.75 mL, 0.75 mmol, 1.50 equiv.) before evaporation of the solvents; colorless solid; 80% (60.8 mg, 401 µmol).

^{1}H-NMR (700 MHz, 298 K, $CDCl_3$) δ = 9.25 (br, 2H, NH_2), 3.41 (sept, $^3J_{HH}$ = 6.6 Hz, 1H, $NCH(CH_3)_2$), 2.73 (d, $^3J_{HH}$ = 6.8 Hz, 2H, NCH_2), 2.27 (tsept, $^3J_{HH}$ = 6.8 Hz, $^3J_{HH}$ = 6.8 Hz, 1H, $NCH_2CH(CH_3)_2$), 1.48 (d, $^3J_{HH}$ = 6.6 Hz, 6H, $NCH(CH_3)_2$), 1.11 (d, $^3J_{HH}$ = 6.8 Hz, 6H, $NCH_2CH(CH_3)_2$); **^{13}C-NMR** (176 MHz, 298 K, $CDCl_3$) δ = 51.4 (NCH_2), 50.8 (NCH), 25.9 (NCH_2CH), 21.1 ($NCH_2CH(CH_3)_2$), 18.9 ($NCH(CH_3)_2$); **HRMS** ($C_7H_{18}N^+$): calc. *m/z* 116.1439, found 116.1441.

7.4.8 (*S*)-*N*-Benzyl-(1-cyclohexylethyl)amine (**5h**)

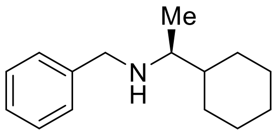

According to the general procedure 7.3, the activation was performed at 90 °C for 1 h, and the reduction was performed with 5 mol%

$B(2,3,6-F_3-C_6H_2)_3$ at 90 °C for 20 h; DCM/EA 20/1 then DCM/EA 5/1; colorless oil, 73% (79.0 mg, 363 µmol); *ee* = 96% (measured as benzamide derivative, see below).

^{1}H-NMR (500 MHz, 303 K, $CDCl_3$) δ = 7.35 – 7.30 (m, 4H, H_{Ar}), 7.26 – 7.22 (m, 1H, H_{Ar}), 3.86 (d, $^2J_{HH}$ = 13.1 Hz, 1H, NCH_2), 3.73 (d, $^2J_{HH}$ = 13.1 Hz, 1H, NCH_2Ph), 2.54 – 2.49 (m, 1H, NCH), 1.86 (br overlapped, 1H, NH), 1.77 – 1.65 (m, 5H, CH_2), 1.42 – 1.36 (m, 1H, NCHC*H*), 1.28 – 1.19 (m, 2H, CH_2), 1.17 – 1.11 (m, 1H, CH_2), 1.04 (d, $^3J_{HH}$ = 6.5 Hz, 3H, NCHC*H*$_3$) 1.03 – 0.97 (m, 2H, CH_2); **^{13}C-NMR** (176 MHz, 298 K, $CDCl_3$) δ = 141.3 (Cq), 128.5 (CH), 128.2 (CH), 126.9 (CH), 57.2 (N*C*HCH), 51.7 (NCH_2Ph), 43.1 (NCH*C*H), 30.0 (CH_2), 28.2 (CH_2), 26.9 (CH_2), 26.8 (CH_2), 26.7 (CH_2), 16.9 (CH_3); **HRMS** ($C_{15}H_{24}N^+$): calc *m/z* 218.1909, found 218.1898.

Derivatization for the determination of enantiomeric excess:

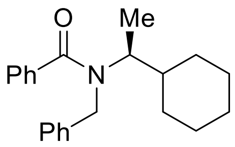

(*S*)-*N*-Benzyl-(1-cyclohexylethyl)amine (**5h**) (69.6 mg, 320 µmol, 1.00 equiv.) and triethylamine (0.06 mL, 400 µmol, 1.25 equiv.) were dissolved in DCM (0.39 mL, 0.83 M). The solution was cooled in an ice bath and benzoyl chloride (0.04 mL, 320 µmol, 1.00 equiv.) was added. The resulting mixture was stirred at room temperature for 30 minutes. After diluting with DCM, the mixture was washed with aqueous HCl (1 M). The aqueous phase was extracted twice with DCM, and the combined organic phases were dried over $MgSO_4$. The solvent was evaporated under reduced pressure, and the crude product was purified by column chromatography (silica, CH/EA 10/1). The colorless oil was characterized by NMR spectroscopy, mass spectroscopy and HPLC. The racemic amide was prepared in the same way starting from racemic amine.

HPLC (IA, 15 °C, heptane/*i*PrOH 80/20, flow: 0.7 mL/min, 239 nm): t_R = 12.3 min (*R*), t_R = 16.8 min (*S*).

^{1}H-NMR (500 MHz, 303 K, $CDCl_3$) δ = 7.44 – 7.39 (m, 5H, H_{Ar}), 7.33 – 7.30 (m, 3H, H_{Ar}), 7.24 – 7.22 (m, 2H, H_{Ar}), 5.06 (d, $^2J_{HH}$ = 15.2 Hz, 1H, NCH_2), 4.22 (d, $^2J_{HH}$ = 15.2 Hz, 1H, NCH_2), 3.57 (dq, $^3J_{HH}$ = 9.5 Hz, $^3J_{HH}$ = 6.8 Hz, 1H, NCH), 1.72 – 1.58 (m, 5H, CH_2), 1.49 – 1.44 (m, 1H, NCHC*H*), 1.14 (d, $^3J_{HH}$ = 6.7 Hz, 3H, NCHC*H*$_3$), 1.09 – 0.96 (m, 3H, CH_2), 0.67 – 0.59 (m, 2H, CH_2); **^{13}C-NMR** (126 MHz, 303 K, $CDCl_3$) δ = 173.3 (Cq), 139.4 (Cq), 137.6 (Cq), 129.4 (CH), 128.6 (CH), 128.5 (CH), 128.4 (CH), 127.7 (CH), 126.9 (CH), 60.8 (N*C*HCH), 44.5 (NCH_2Ph), 41.9 (NCH*C*H), 30.5 (CH_2), 30.4 (CH_2), 26.3 (CH_2), 26.1 (CH_2), 26.0 (CH_2), 18.1 (CH_3); **HRMS** ($C_{22}H_{28}NO^+$): calc *m/z* 322.2171, found 322.2153.

HPLC results:

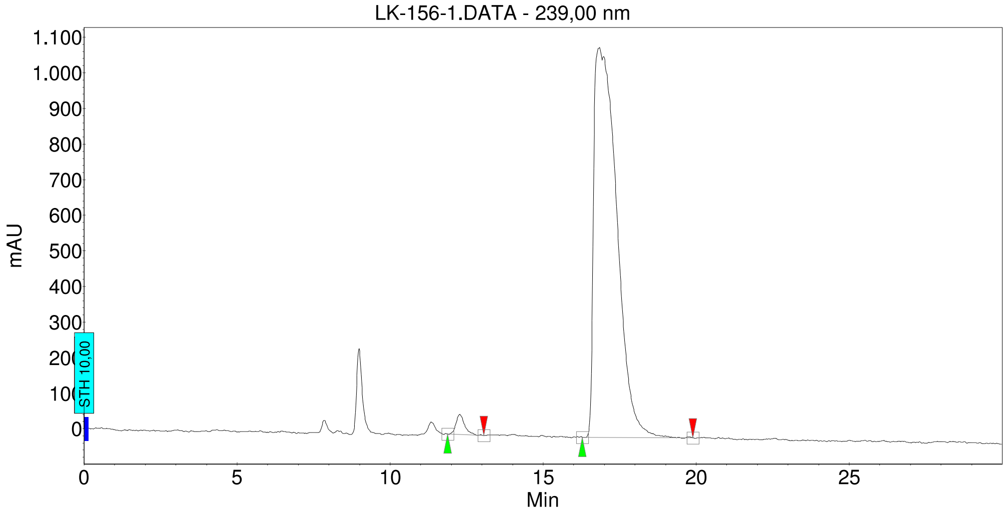

Peak results :

Index	Name	Time [Min]	Quantity [% Area]	Height [mAU]	Area [mAU.Min]	Area % [%]
1	UNKNOWN	12,28	1,86	55,9	18,0	1,860
2	UNKNOWN	16,84	98,14	1095,5	952,0	98,140
Total			100,00	1151,5	970,0	100,000

racemic sample:

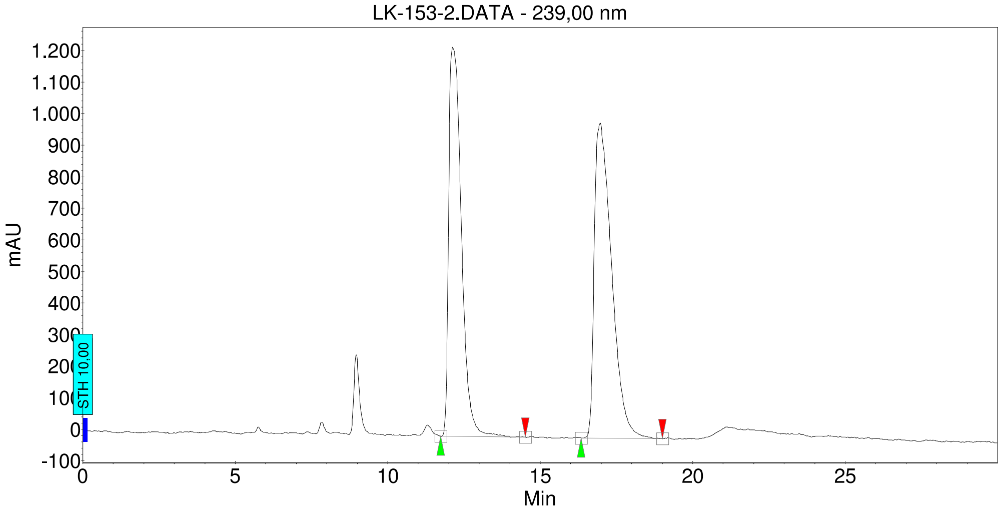

Peak results :

Index	Name	Time [Min]	Quantity [% Area]	Height [mAU]	Area [mAU.Min]	Area % [%]
1	UNKNOWN	12,12	47,63	1233,9	597,0	47,630
2	UNKNOWN	16,97	52,37	998,0	656,4	52,370
Total			100,00	2231,9	1253,4	100,000

7.4.9 *N*-Isopropyl-(4-(phenylethynyl)benzyl)amine (**5i**)

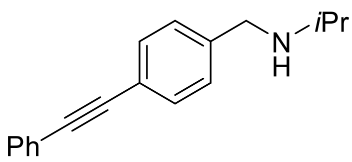

According to the general procedure 7.3, the activation was performed at 90 °C for 4 h, and the reduction was performed with 5 mol% B(2,3,6-F_3-C_6H_2)$_3$ at 90 °C for 20 h; DCM/EA 20/1 then EA; light yellow oil, 89% (111 mg, 445 µmol).

^{1}H-NMR (700 MHz, 298 K, $CDCl_3$) δ = 7.53 – 7.52 (m, 2H, H_{Ar}), 7.50 – 7.49 (m, 2H, H_{Ar}), 7.36 – 7.30 (m, 5H, H_{Ar}), 3.80 (s, 2H, NC*H*$_2$), 2.85 (sept, $^3J_{HH}$ = 6.2 Hz, 1H, NC*H*(CH_3)$_2$), 1.32 (br, 1H, NH), 1.10 (d, $^3J_{HH}$ = 6.2 Hz, 6H, NCH(C*H*$_3$)$_2$); **^{13}C-NMR** (176 MHz, 298 K, $CDCl_3$) δ = 141.4 (Cq), 131.8 (CH), 131.7 (CH), 128.5 (CH), 128.3 (CH), 128.2 (CH), 123.5 (Cq), 121.8 (Cq), 89.5 (Cq), 89.2 (Cq), 51.5 (NCH_2), 48.3 (NCH), 23.1 (CH_3); **HRMS** ($C_{18}H_{20}N^+$): calc *m/z* 250.1596, found 250.1572.

7.4.10 *N*-Benzyl-2-(cinnamoyloxy)ethanamine hydrochloride (**5j**)

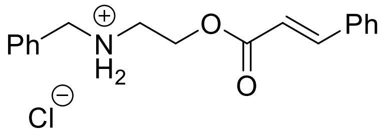

According to the general procedure 7.3, the activation was performed at 70 °C for 7 h with the addition of 2,6-Lutidine (53.6 mg, 500 µmol, 1.00 equiv.), and the reduction was performed with 10 mol% B(2,3,6-F_3-C_6H_2)$_3$ at 70 °C for 20 h; DCM/EA 10/1.

For further purification, the yellow oil was dissolved in Et_2O and HCl in Et_2O (0.75 mL, 0.75 mmol, 1.50 equiv.) was added. The resulting suspension was centrifuged (3000 rpm for 10 min) and the supernatant solution was removed. The ammonia salt was washed twice with Et_2O. White solid, 58% (92.1 mg, 290 µmol).

^{1}H-NMR (700 MHz, 298 K, $(CD_3)_2SO$) δ = 9.72 (br, 2H, NH_2), 7.83 (d, $^3J_{HH}$ = 16.1 Hz, 1H, PhC*H*=CH), 7.73 – 7.72 (m, 2H, H_{Ar}), 7.63 – 7.61 (m, 2H, H_{Ar}), 7.45 – 7.40 (m, 6H, H_{Ar}), 6.63 (d, $^3J_{HH}$ = 16.1 Hz, 1H, PhCH=C*H*), 4.46 (t, $^3J_{HH}$ = 5.4 Hz, 2H, OCH_2), 4.22 (br, 2H, NCH_2Ph), 3.26 (br, 2H, OCH_2C*H*$_2$); **^{13}C-NMR** (176 MHz, 298 K, $(CD_3)_2SO$) δ = 165.8 (Cq), 145.2 (CH), 134.0 (Cq), 131.9 (Cq), 130.6 (CH), 130.2 (CH), 129.0 (CH), 128.9 (CH), 128.6 (CH), 128.4 (CH), 117.5 (CH), 59.6 (CH_2), 50.1 (CH_2), 45.0 (CH_2); **HRMS** ($C_{18}H_{20}NO_2^+$): calc *m/z* 282.1494, found 282.1490.

7.4.11 *N*-Isopropyl-(4-methoxybenzyl)amine (**5k**)

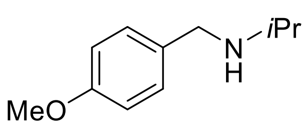

According to the general procedure 7.3, the activation was performed at 90 °C for 1 h, and the reduction was performed with 5 mol%

$B(2,3,6\text{-}F_3\text{-}C_6H_2)_3$ at 90 °C for 20 h; DCM/EA 50/1 then CH/EA 1/1; yellow oil, 95% (85.1 mg,475 µmol).

^{1}H-NMR (700 MHz, 298 K, $CDCl_3$) δ = 7.25 – 7.23 (m, 2H, H_{Ar}), 6.87 – 6.85 (m, 2H, H_{Ar}), 3.79 (s, 3H, OCH_3), 3.72 (s, 2H, NCH_2), 2.85 (sept, $^3J_{HH}$ = 6.3 Hz, 1H, $NCH(CH_3)_2$), 1.97 (br, 1H, NH), 1.10 (d, $^3J_{HH}$ = 6.3 Hz, 6H, $NCH(CH_3)_2$); **^{13}C-NMR** (176 MHz, 298 K, $CDCl_3$) δ = 158.8 (Cq), 132.5 (Cq), 129.5 (CH), 114.0 (CH), 55.4 (OCH_3), 51.0 (CH_2), 48.1 (NCH), 22.9 ($CHCH_3$); **HRMS** ($C_{11}H_{18}NO^+$): calc *m/z* 180.1388, found 180.1384.

7.4.12 *N*-Isopropyl-(4-nitrobenzyl)amine (**5l**)

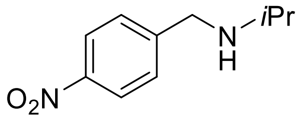

According to the general procedure 7.3, the activation was performed at 90 °C for 10 h, and the reduction was performed with 10 mol% $B(2,3,6\text{-}F_3\text{-}C_6H_2)_3$ at 90 °C for 40 h; DCM/EA 50/1 then EA; yellow oil; 86% (83.9 mg, 432 µmol).

^{1}H-NMR (700 MHz, 298 K, $CDCl_3$) δ = 8.18 – 8.17 (m, 2H, H_{Ar}), 7.52 – 7.50 (m, 2H, H_{Ar}), 3.89 (s, 2H, NCH_2), 2.85 (sept, $^3J_{HH}$ = 6.2 Hz, 1H, $NCH(CH_3)_2$), 1.40 (br, 1H, NH), 1.10 (d, $^3J_{HH}$ = 6.2 Hz, 6H, $NCH(CH_3)_2$); **^{13}C-NMR** (176 MHz, 298 K, $CDCl_3$) δ = 148.9 (Cq), 147.1 (Cq), 128.7 (CH), 123.7 (CH), 51.0 (CH_2), 48.6 (NCH), 23.1 (CH_3); **HRMS** ($C_{10}H_{15}N_2O_2^+$): calc *m/z* 195.1134, found 195.1134.

7.5 NMR spectra

^{1}H-NMR (500 MHz, 303 K, $CDCl_3$)

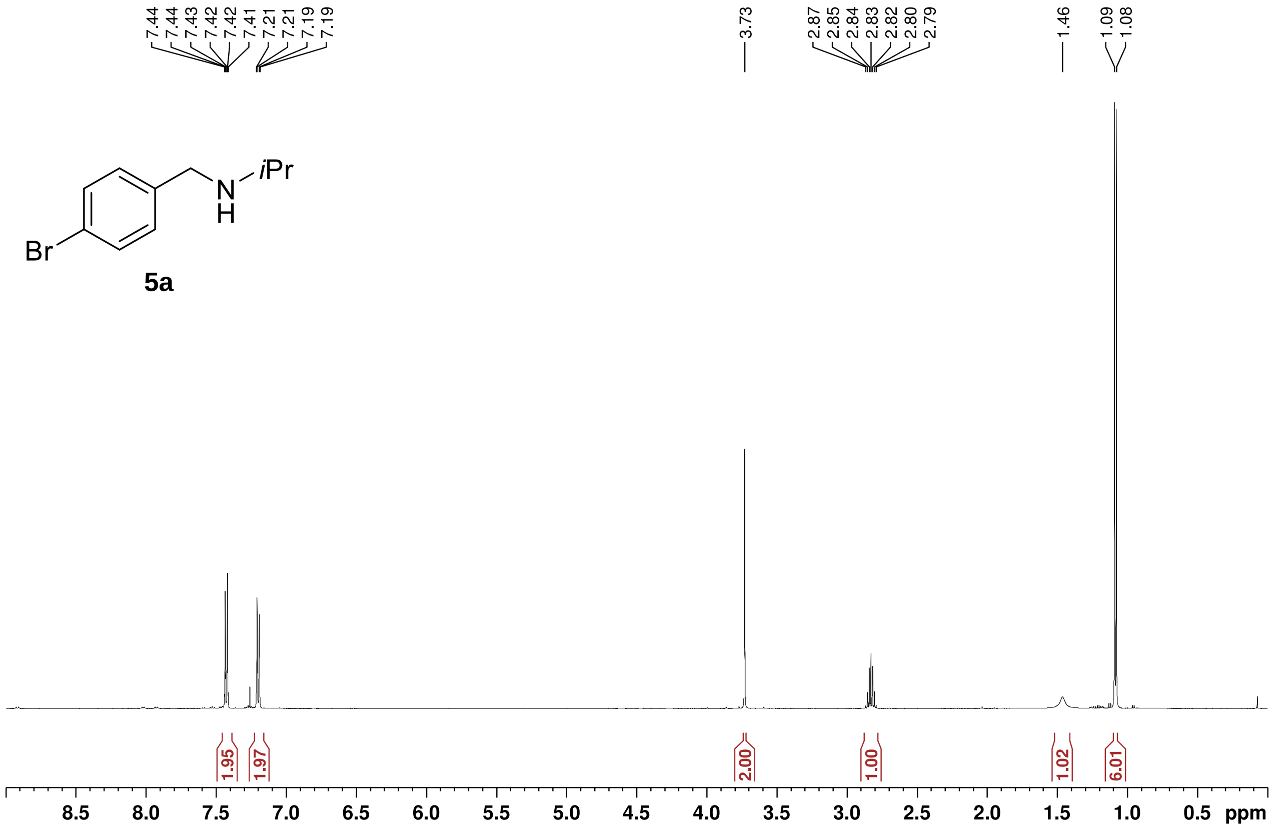

^{13}C-NMR (126 MHz, 303 K, $CDCl_3$)

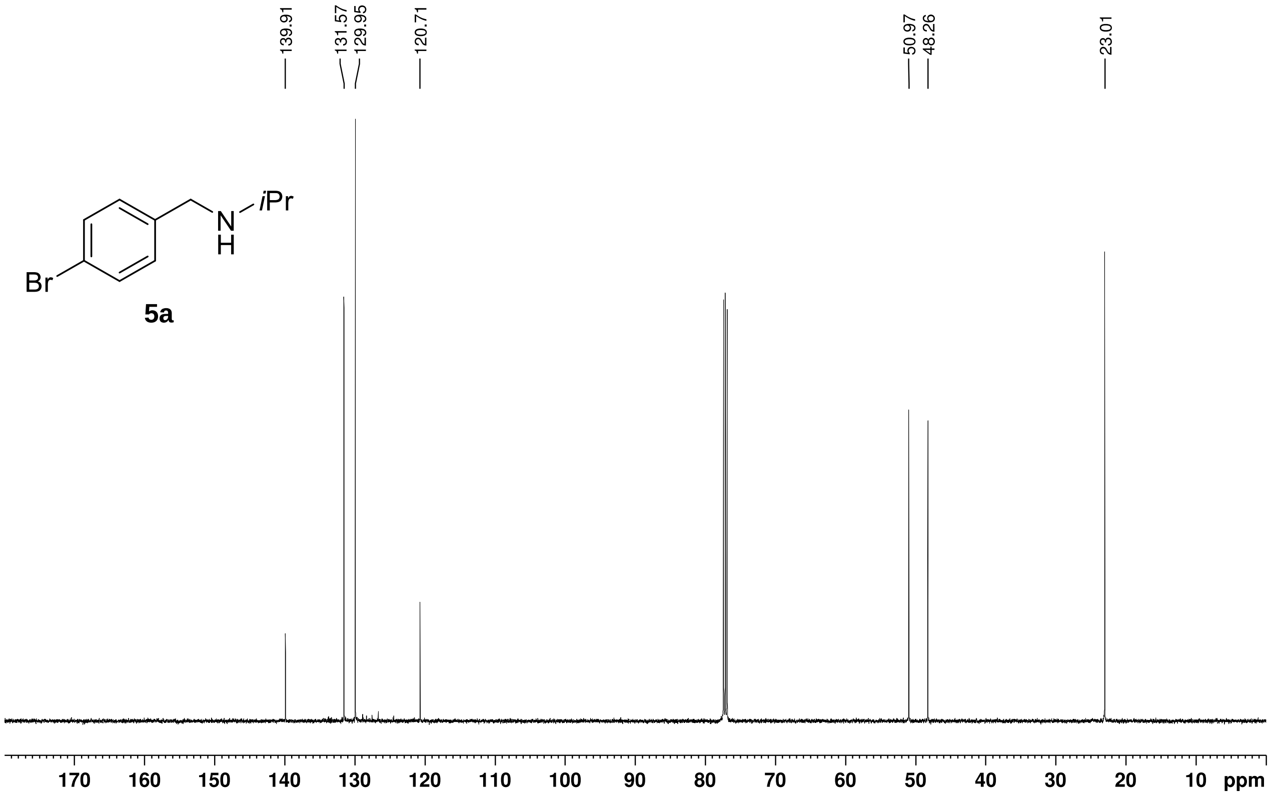

¹H-NMR (500 MHz, 303 K, $CDCl_3$)

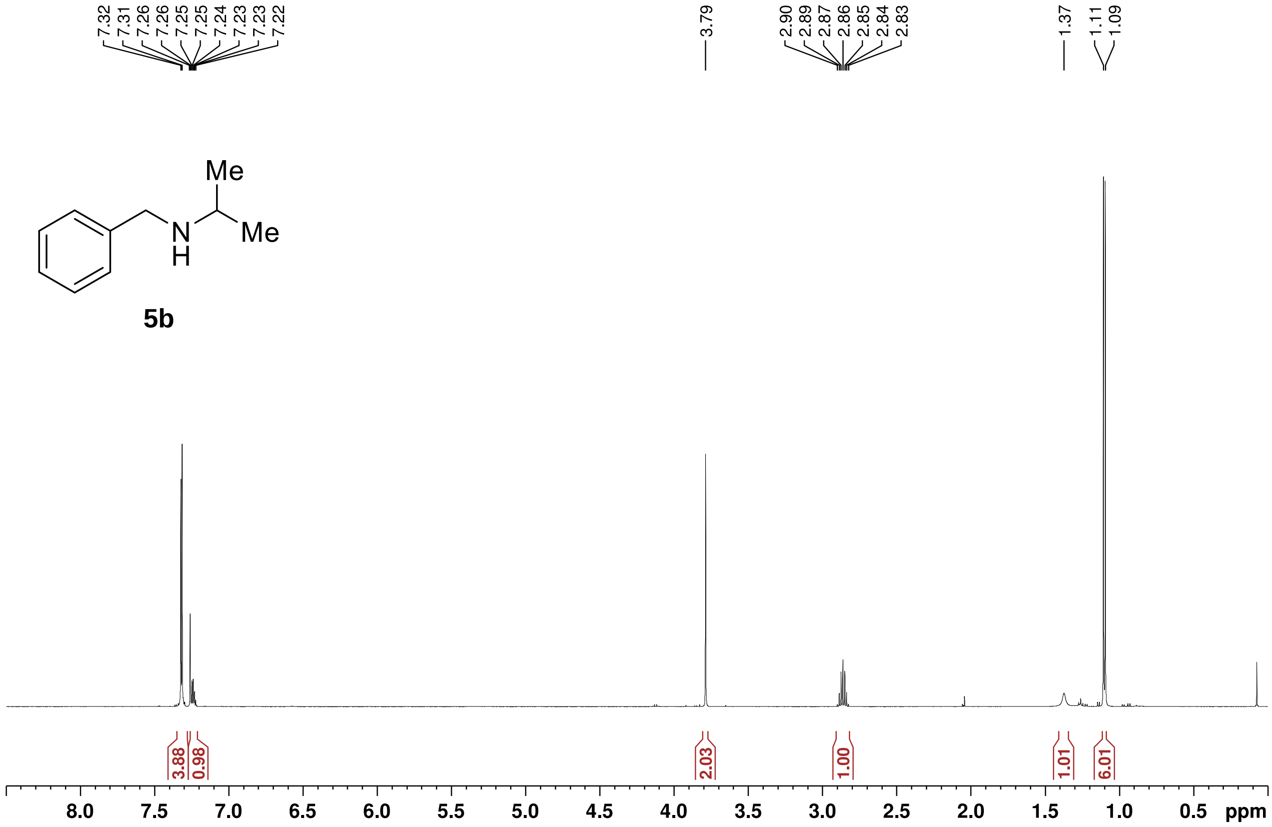

¹³C-NMR (126 MHz, 303 K, $CDCl_3$)

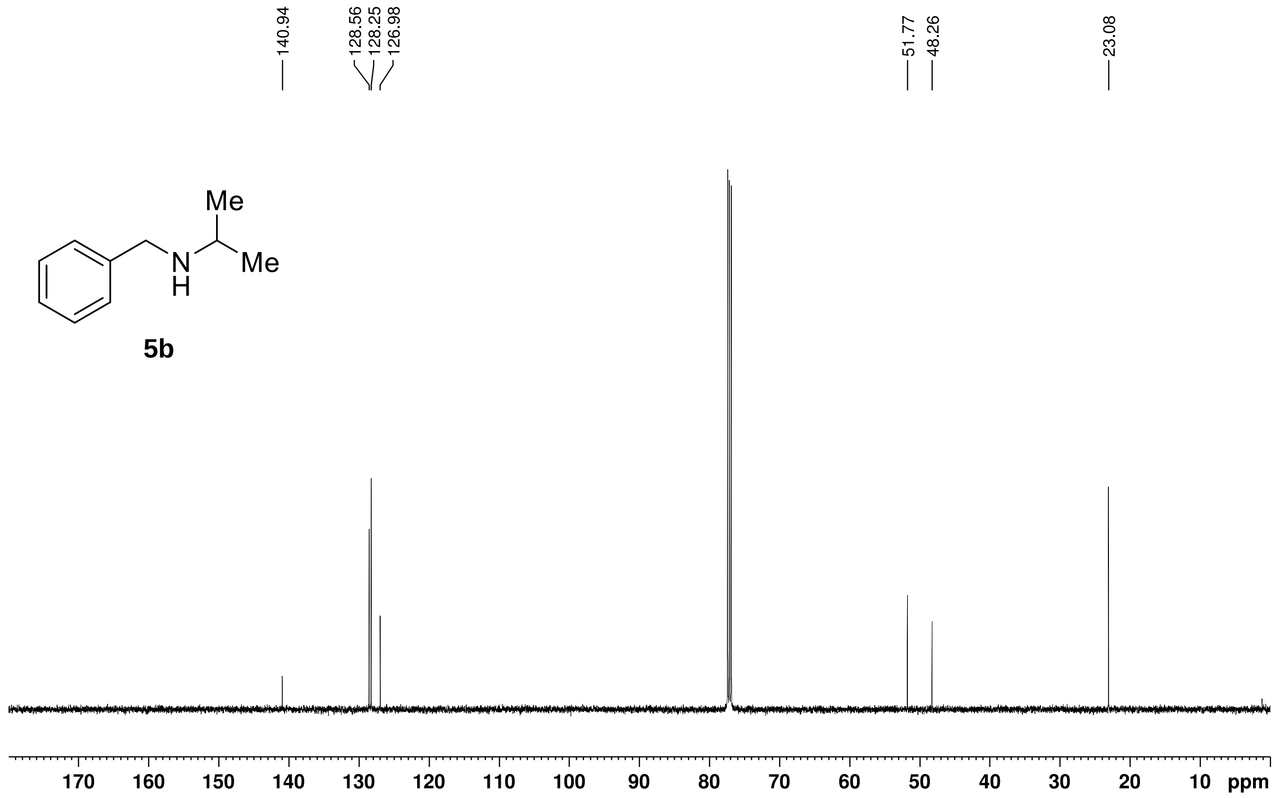

^{1}**H-NMR** (500 MHz, 303 K, $CDCl_3$)

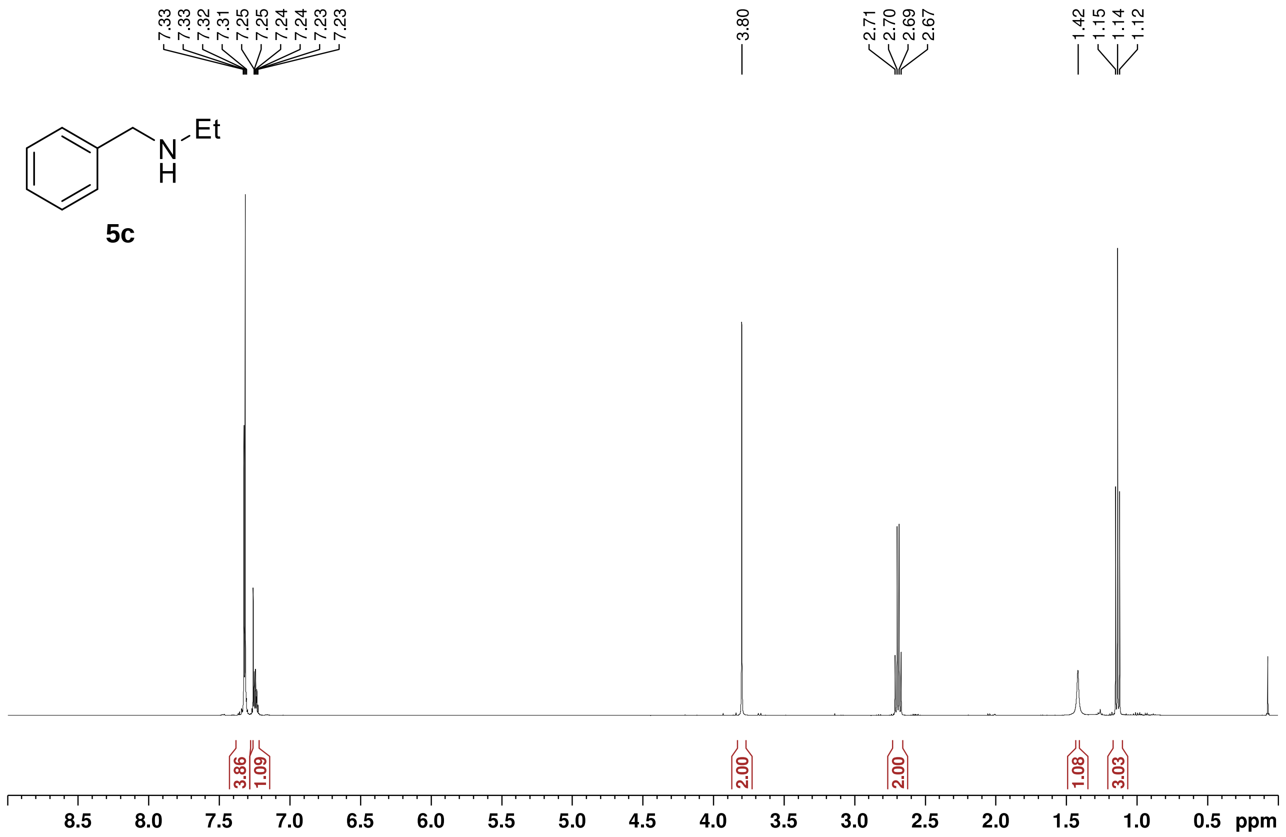

^{13}**C-NMR** (126 MHz, 303 K, $CDCl_3$)

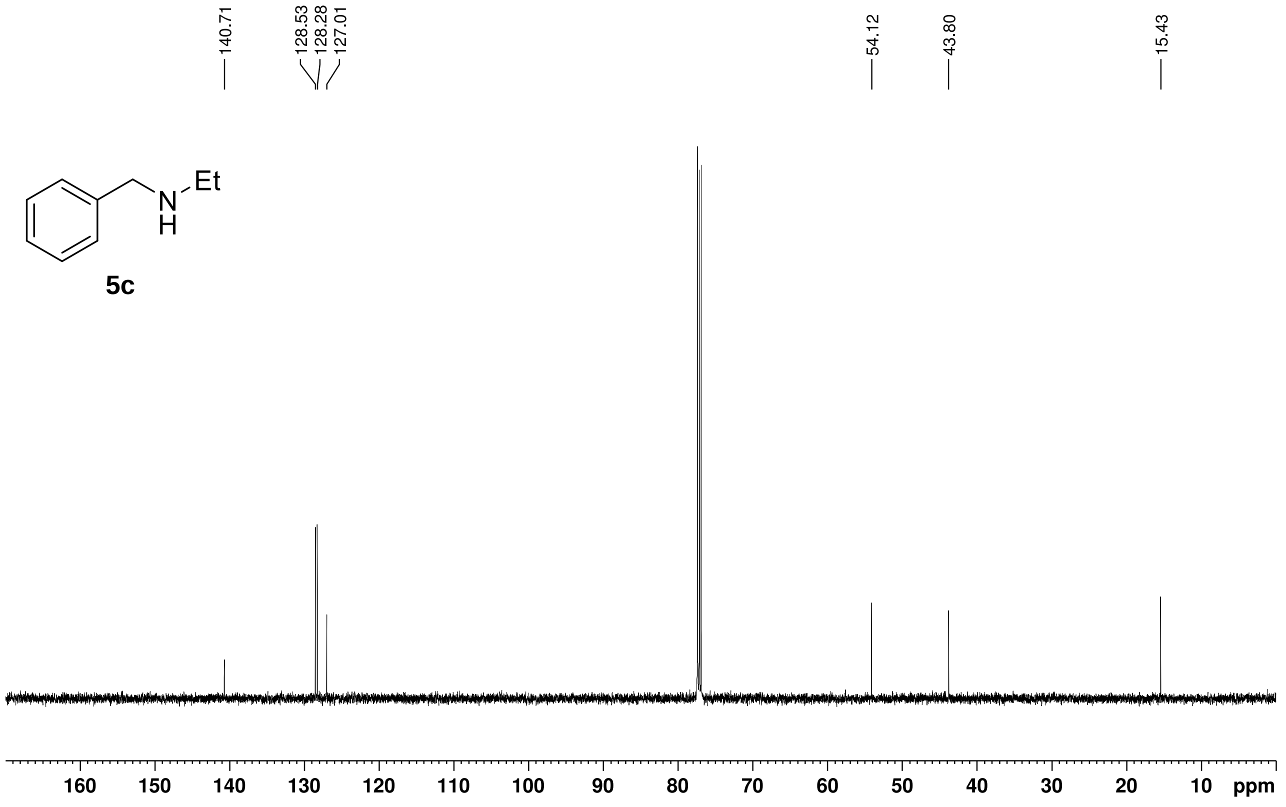

^{1}H-NMR (700 MHz, 298 K, $CDCl_3$)

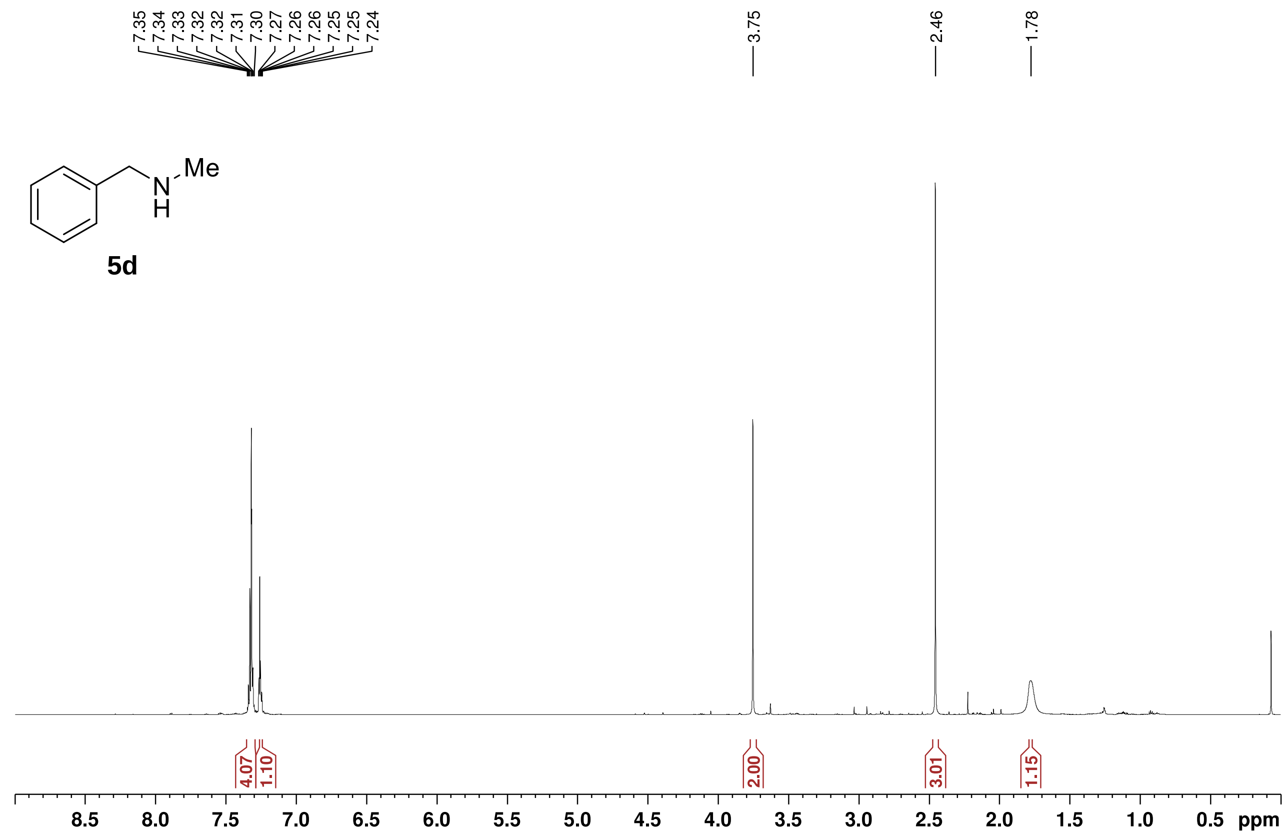

^{13}C-NMR (176 MHz, 298 K, $CDCl_3$)

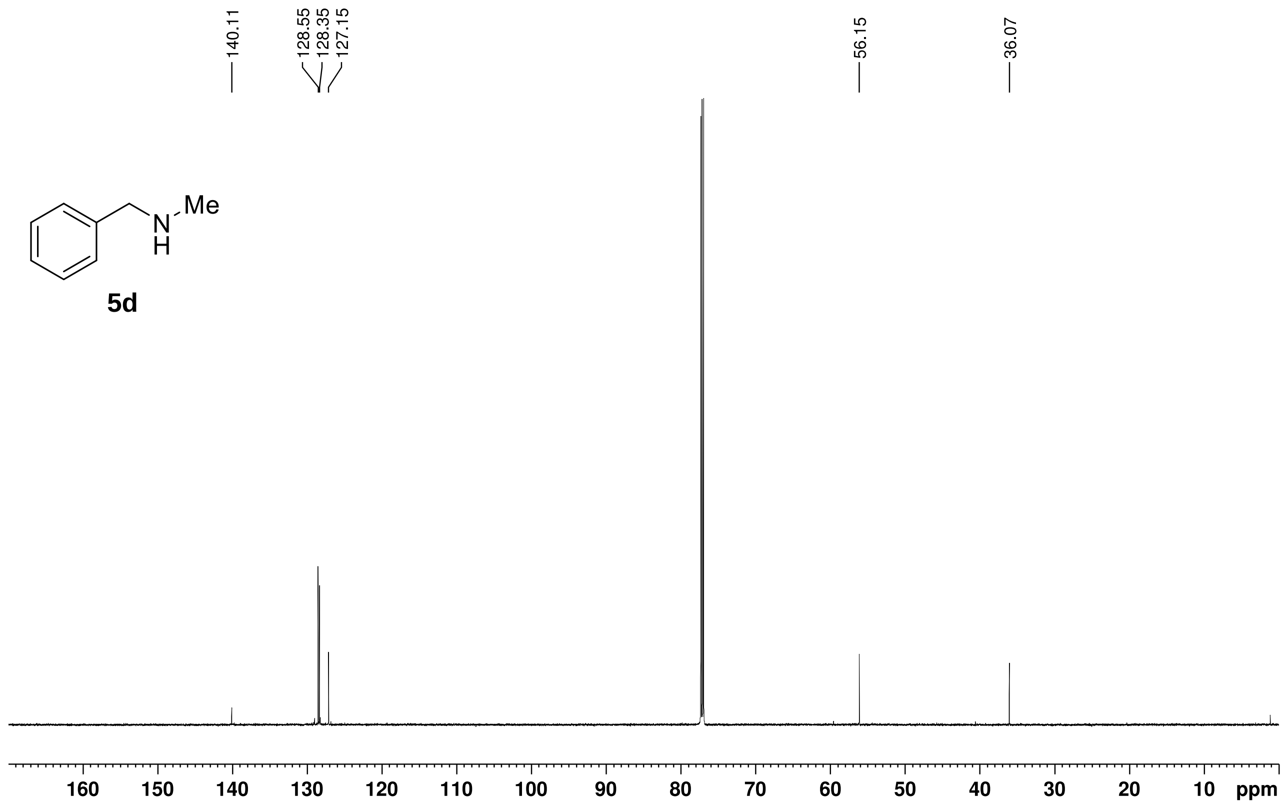

^{1}H-NMR (700 MHz, 298 K, $CDCl_3$)

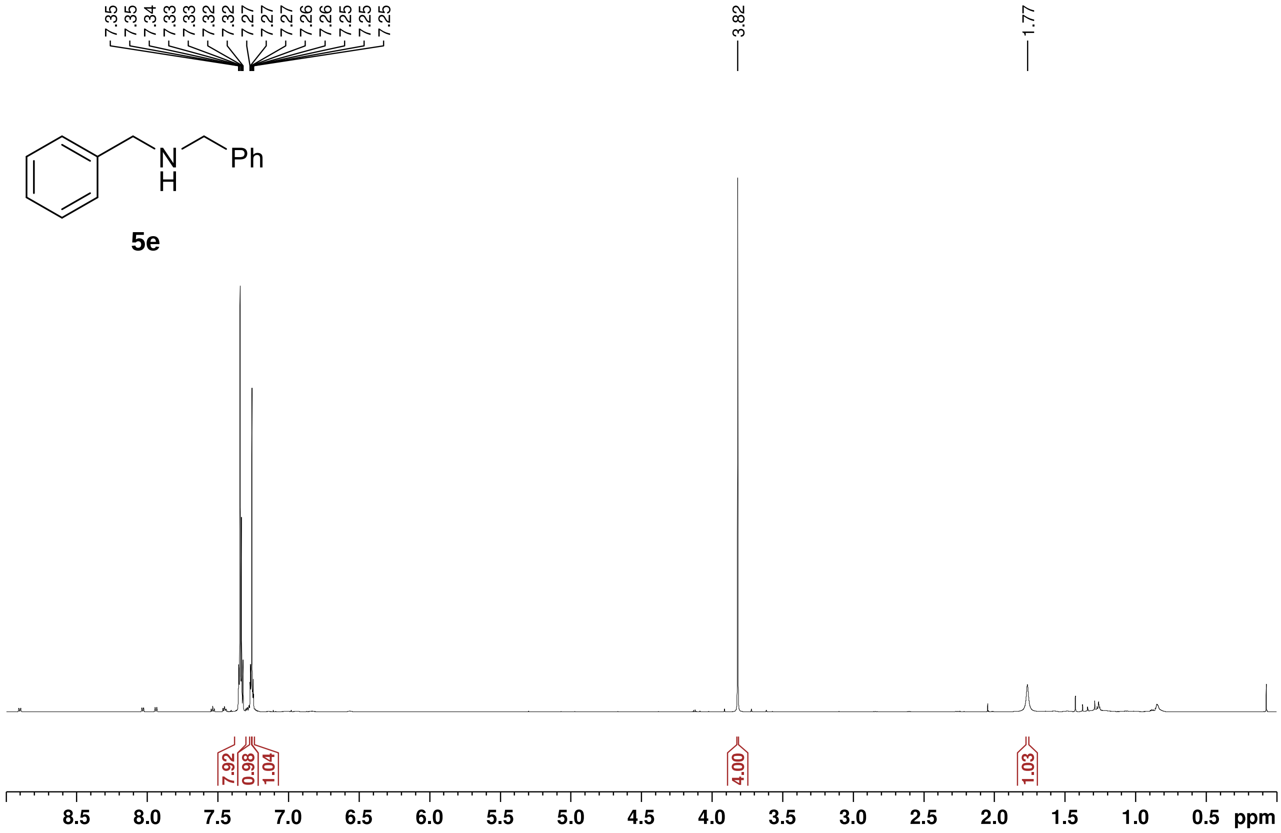

^{13}C-NMR (176 MHz, 298 K, $CDCl_3$)

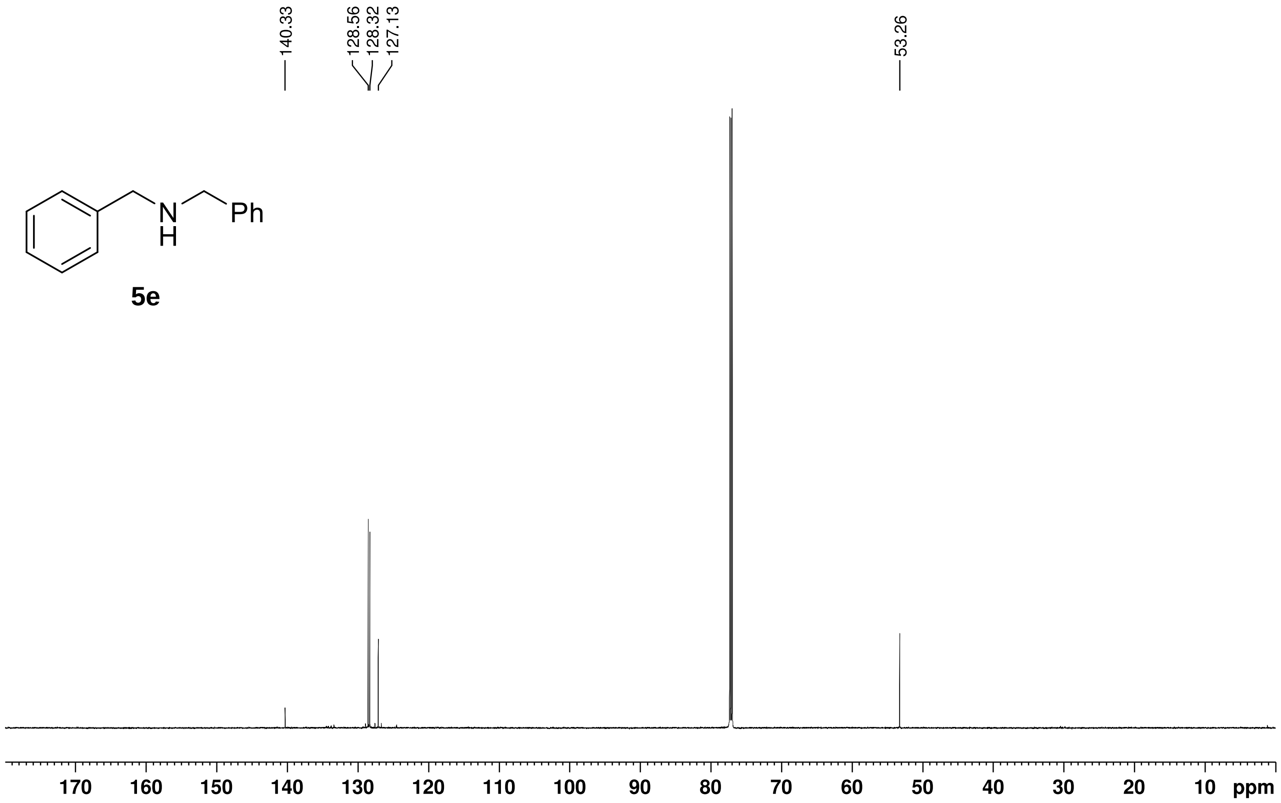

^{1}H-NMR (700 MHz, 298 K, $CDCl_3$)

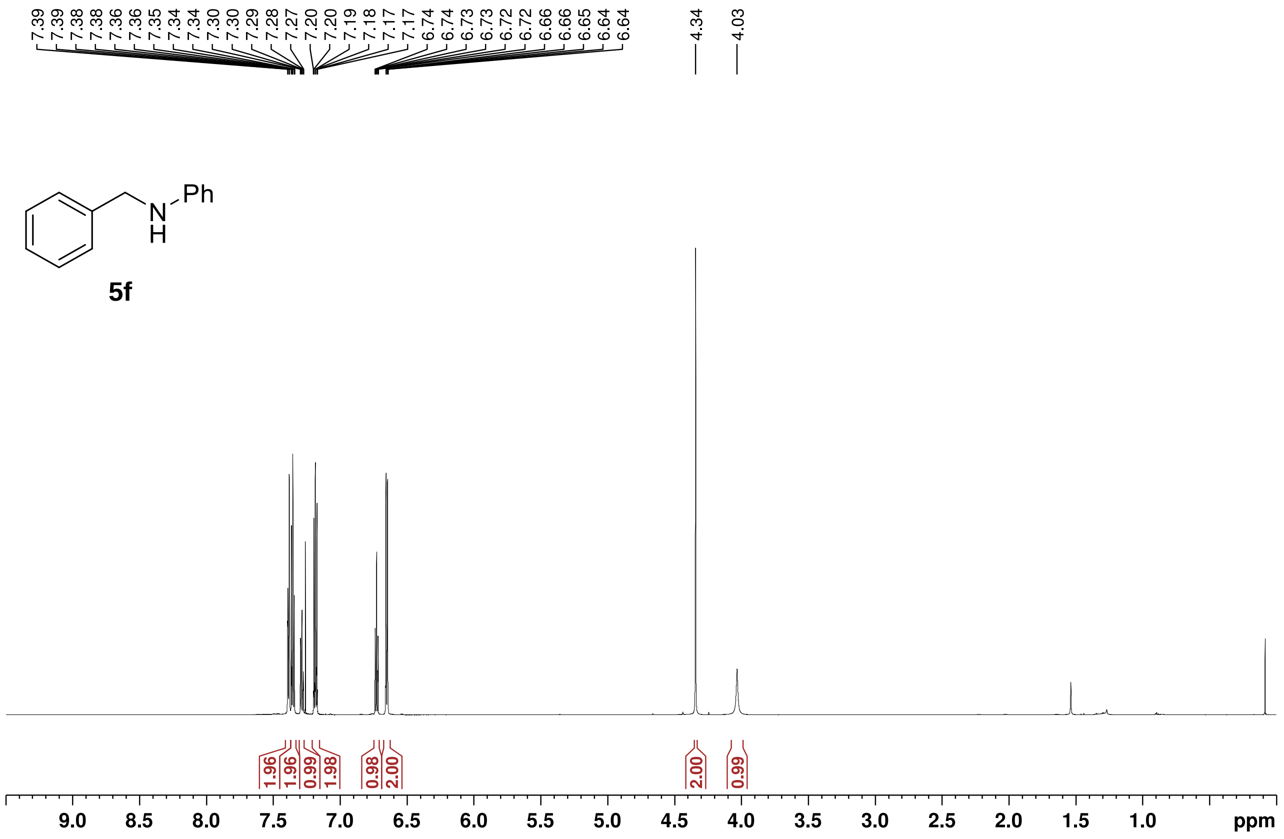

^{13}C-NMR (176 MHz, 298 K, $CDCl_3$)

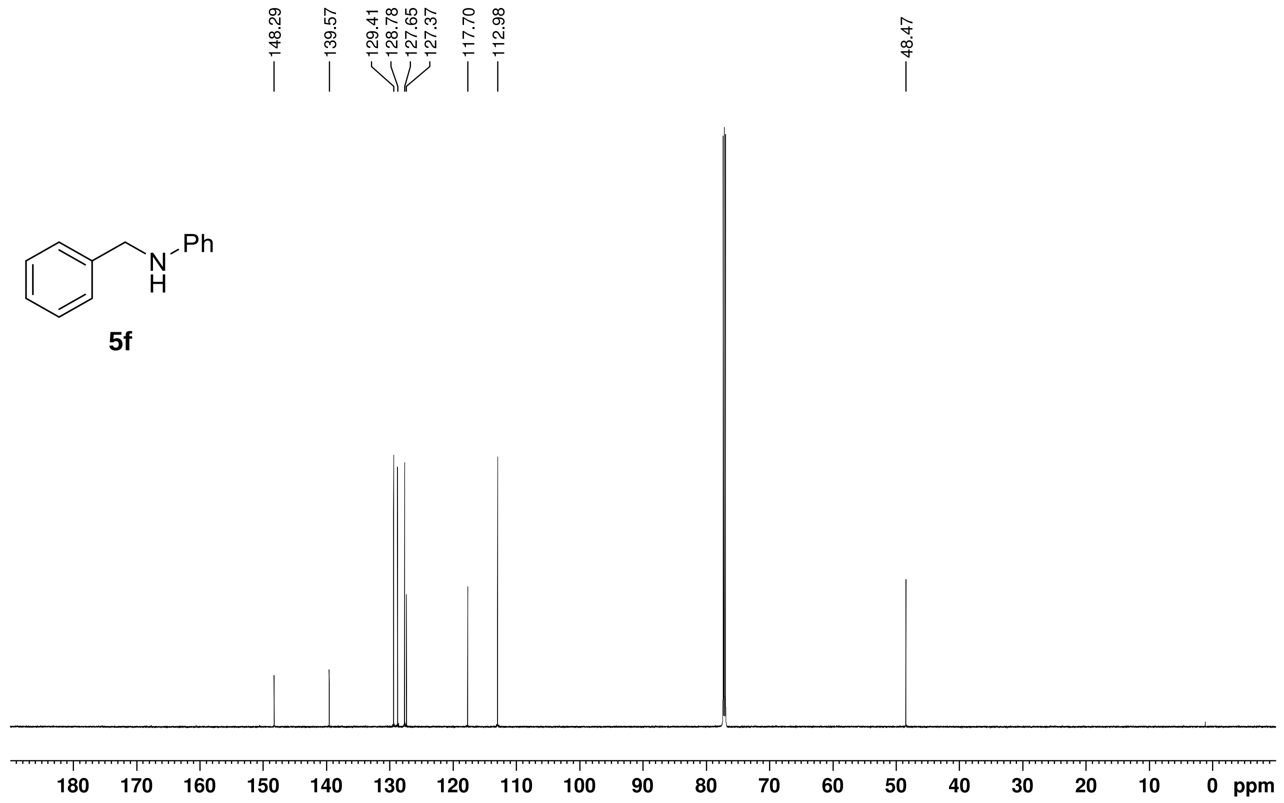

^{1}H-NMR (700 MHz, 298 K, $CDCl_3$)

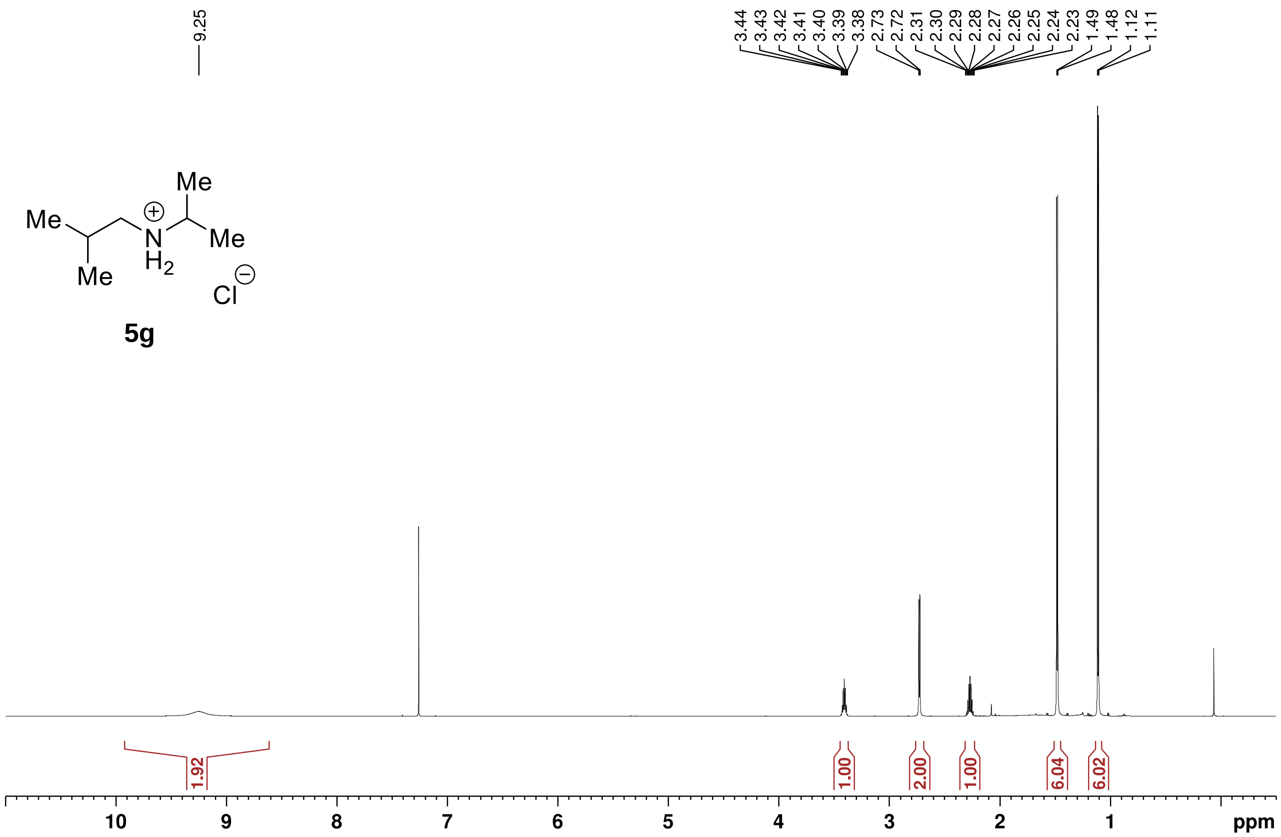

^{13}C-NMR (176 MHz, 298 K, $CDCl_3$)

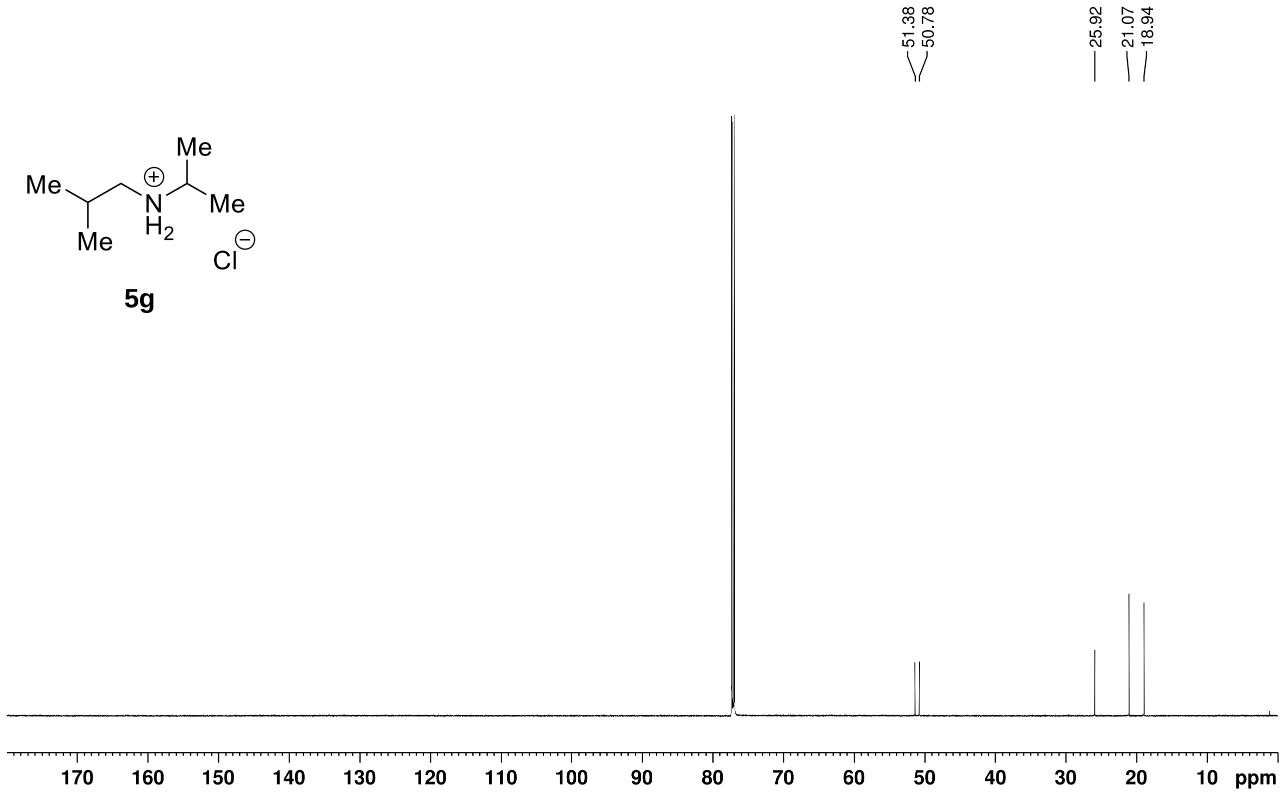

^{1}H-NMR (500 MHz, 303 K, $CDCl_3$)

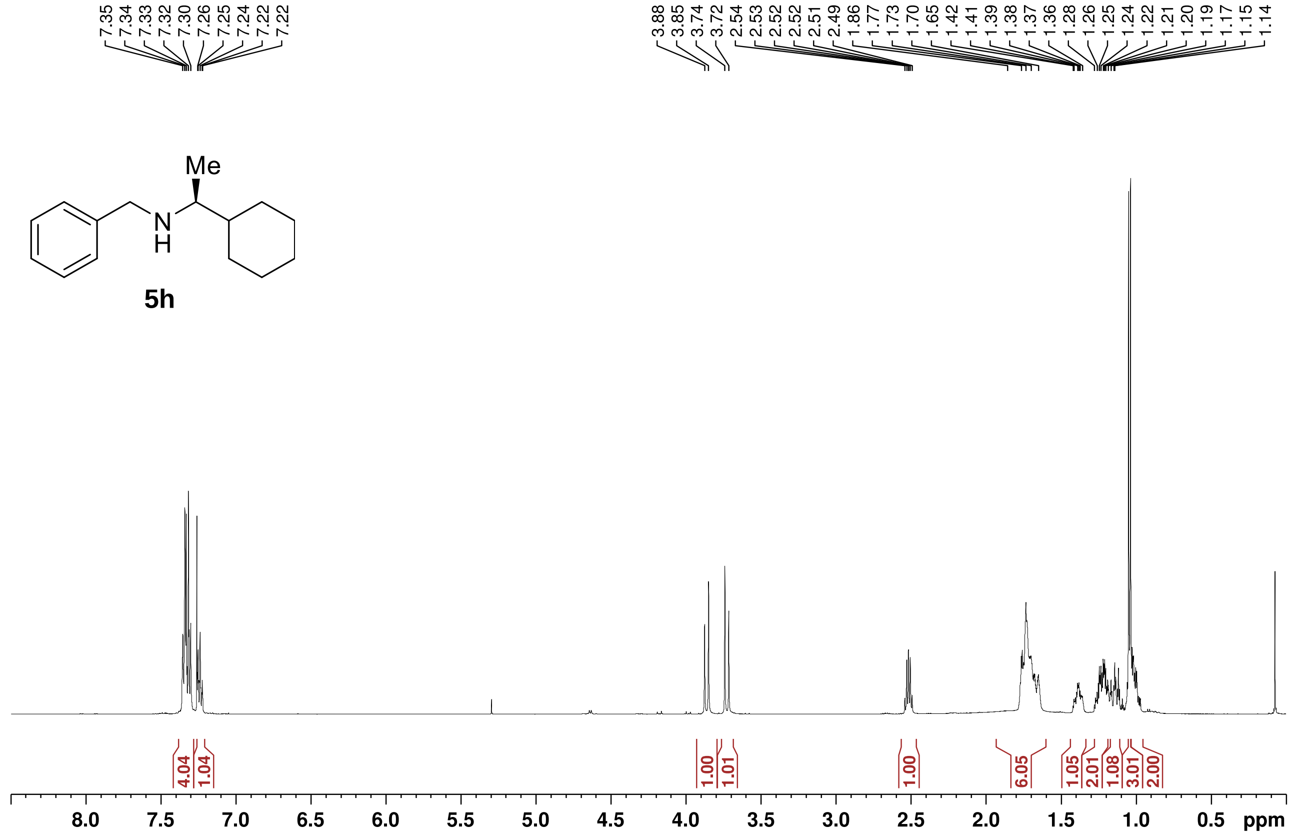

^{13}C-NMR (126 MHz, 303 K, $CDCl_3$)

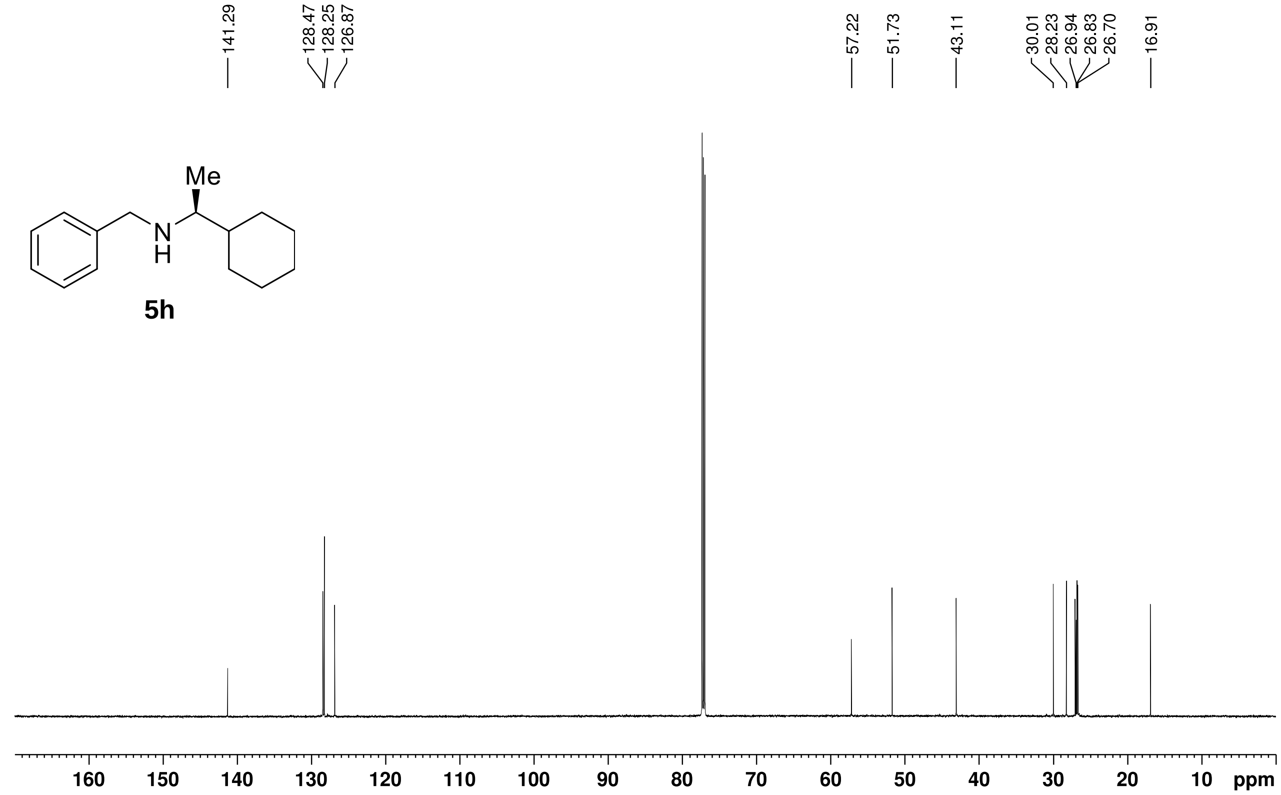

^{1}H-NMR (500 MHz, 303 K, $CDCl_3$)

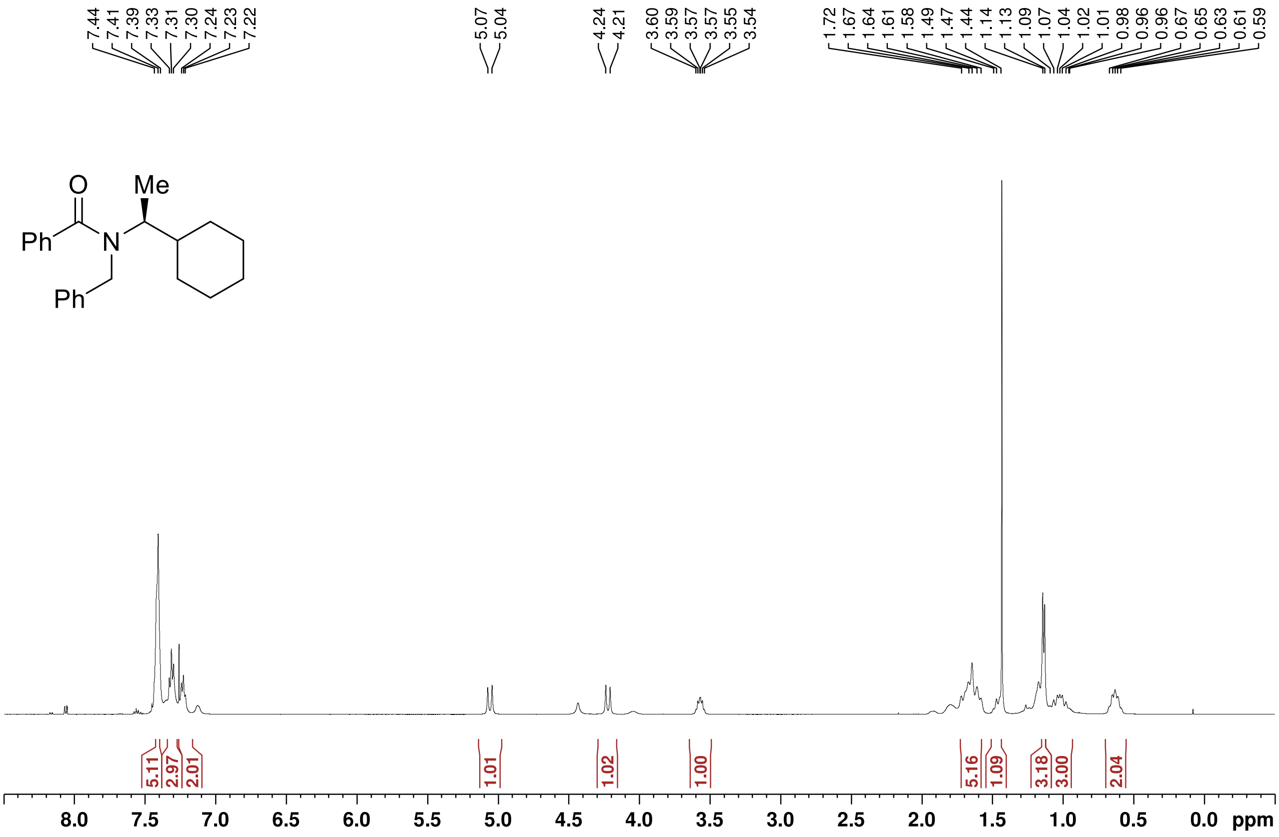

^{13}C-NMR (126 MHz, 303 K, $CDCl_3$)

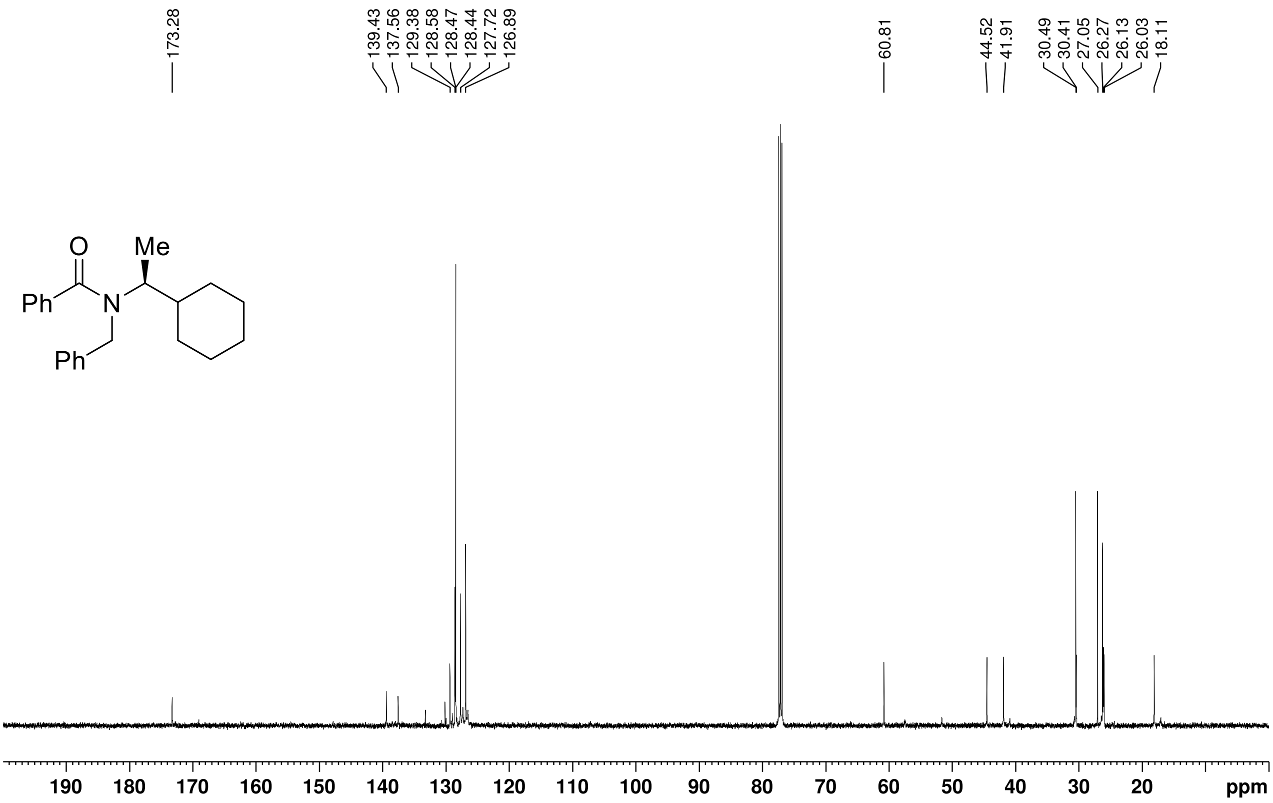

^{1}H-NMR (700 MHz, 298 K, $CDCl_3$)

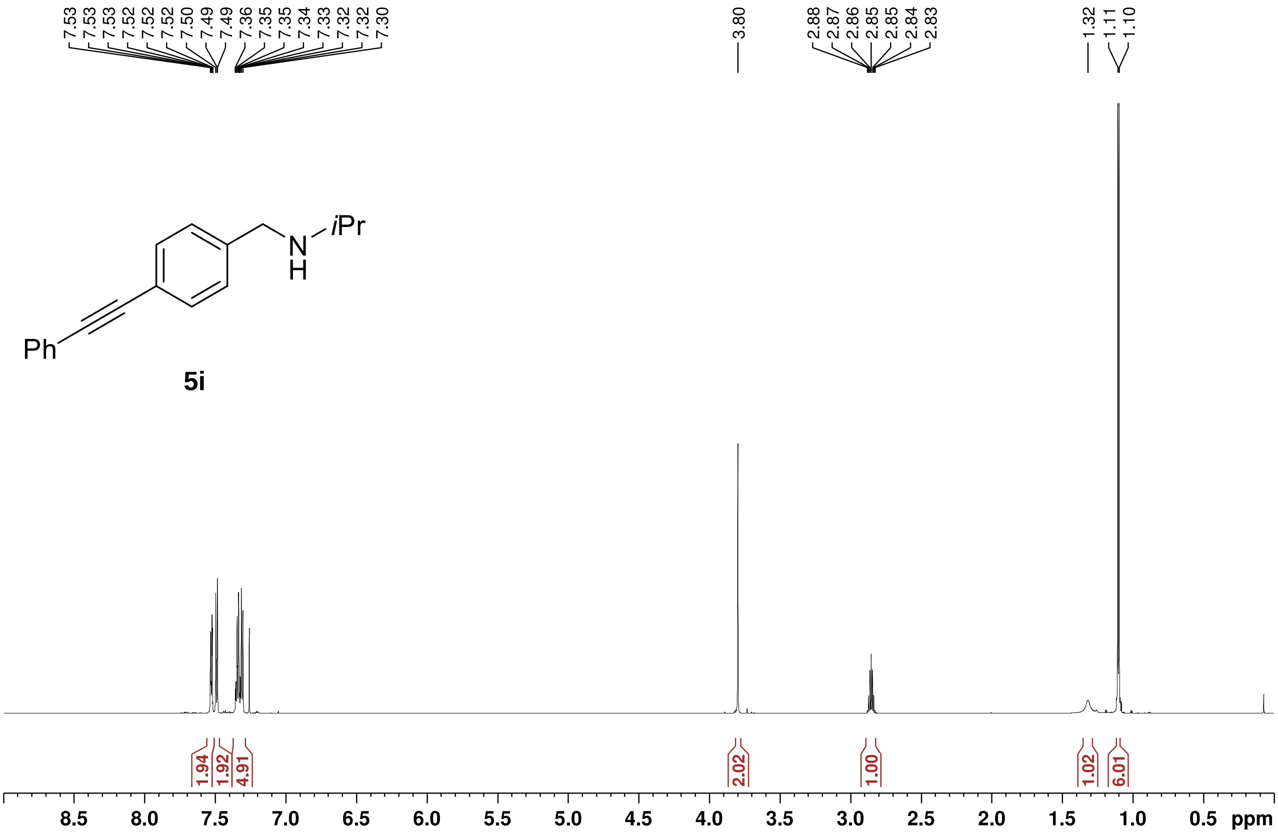

^{13}C-NMR (176 MHz, 298 K, $CDCl_3$)

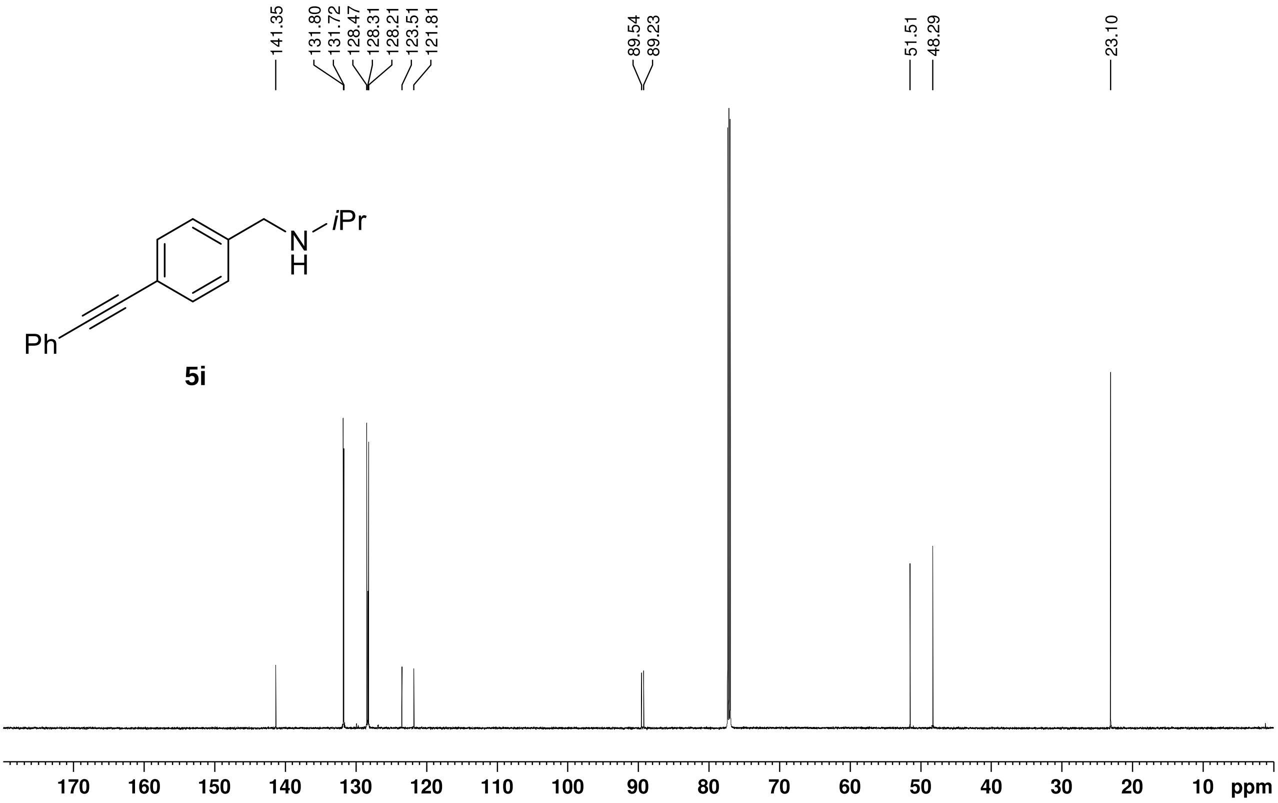

^{1}H-NMR (700 MHz, 298 K, $(CD_3)_2SO$)

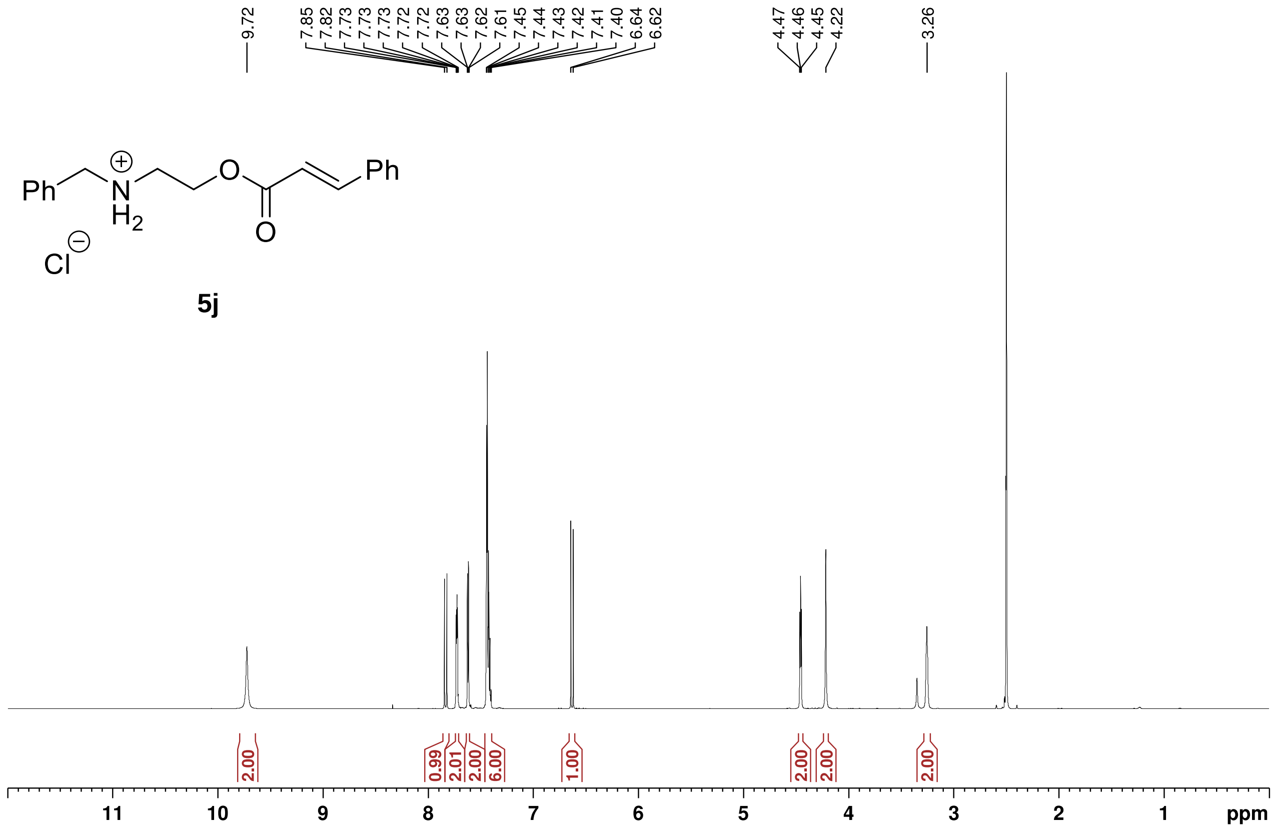

^{13}C-NMR (176 MHz, 298 K, $(CD_3)_2SO$)

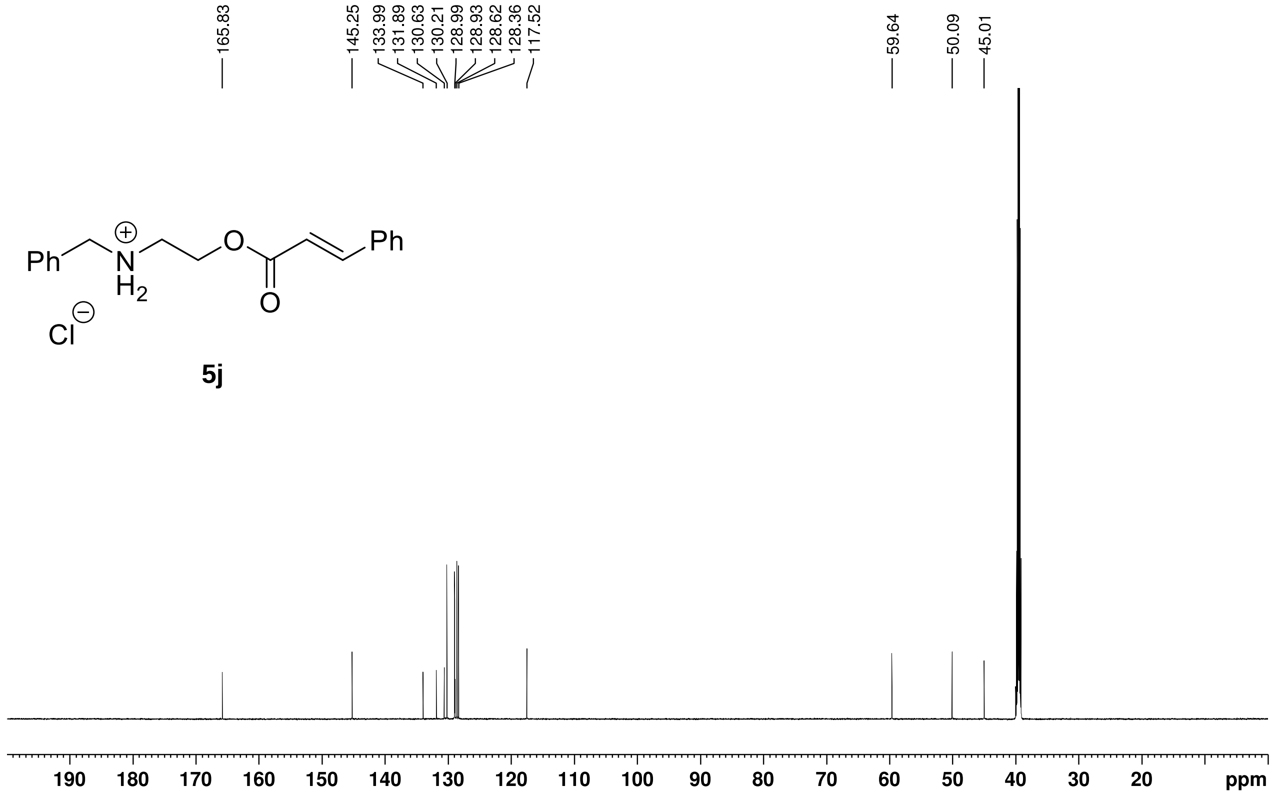

^{1}H-NMR (700 MHz, 298 K, $CDCl_3$)

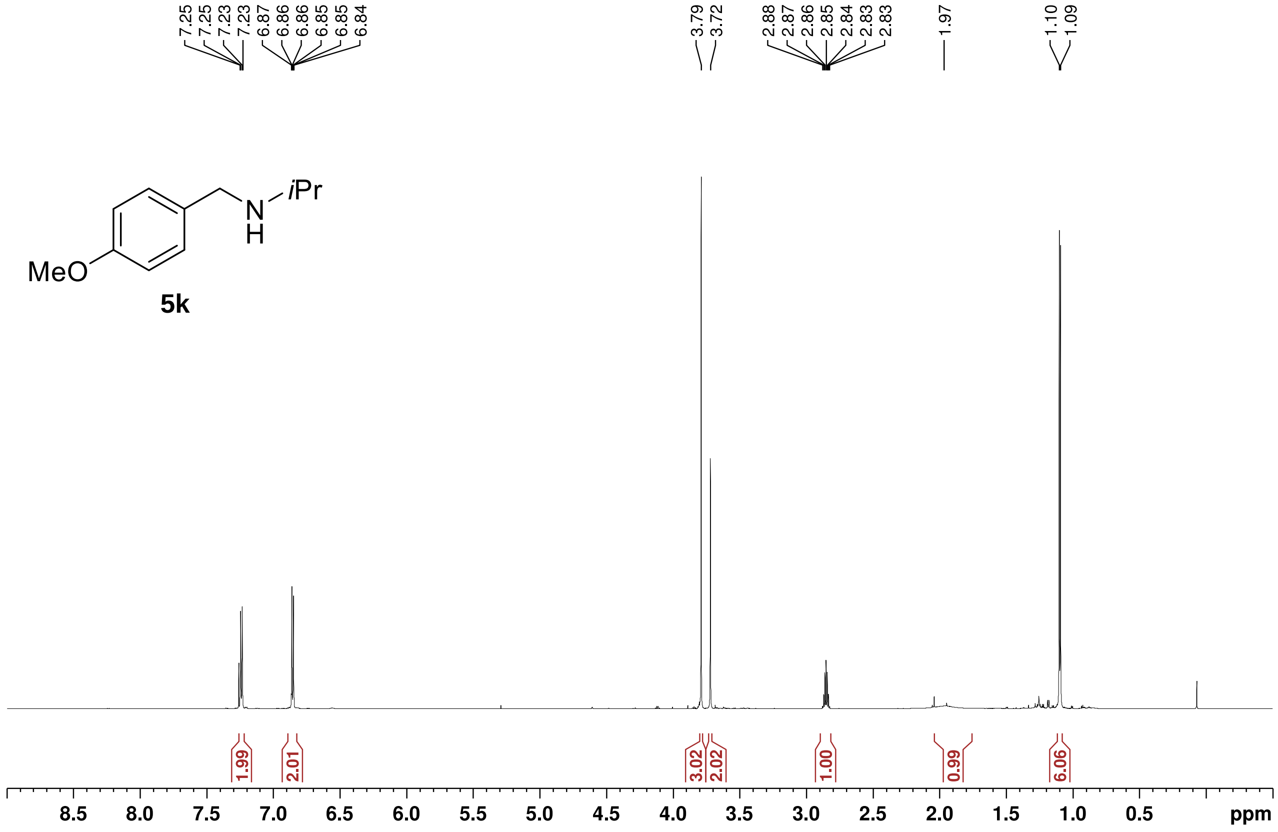

^{13}C-NMR (176 MHz, 298 K, $CDCl_3$)

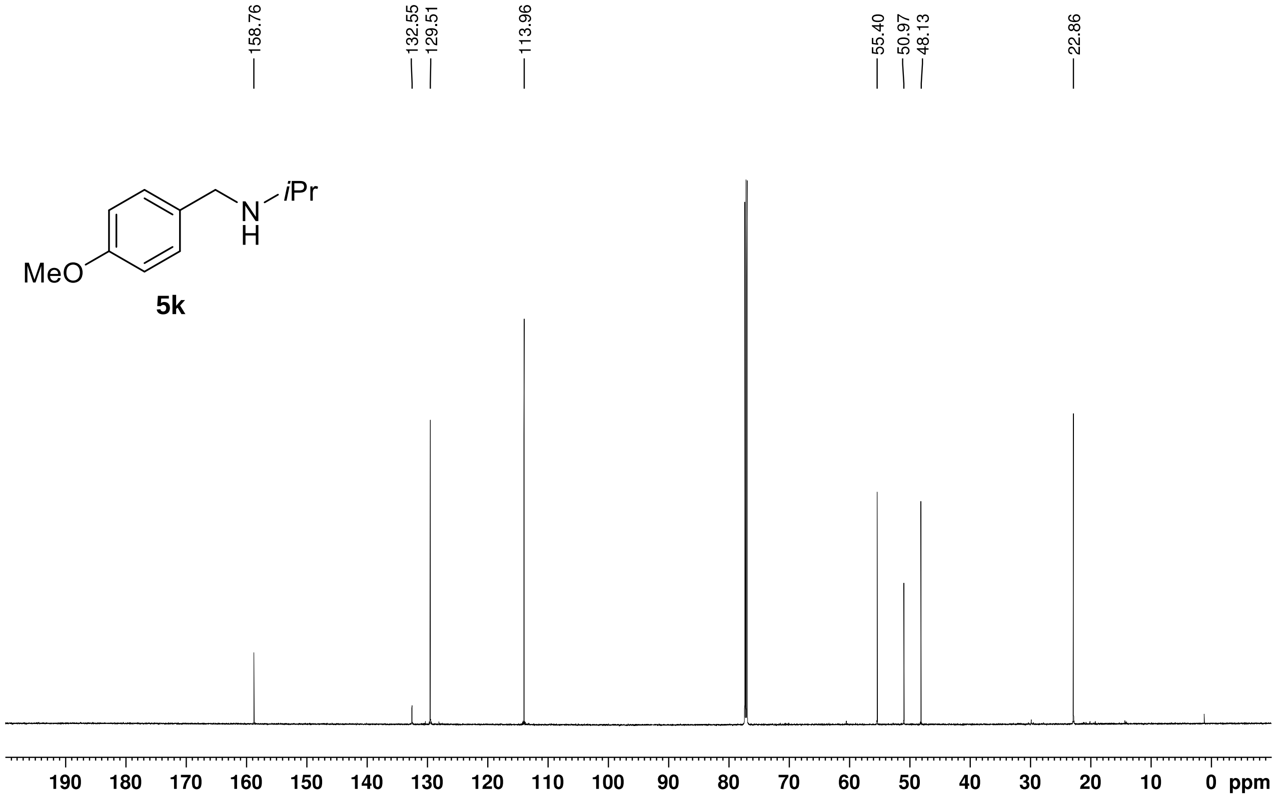

^{1}**H-NMR** (700 MHz, 298 K, $CDCl_3$)

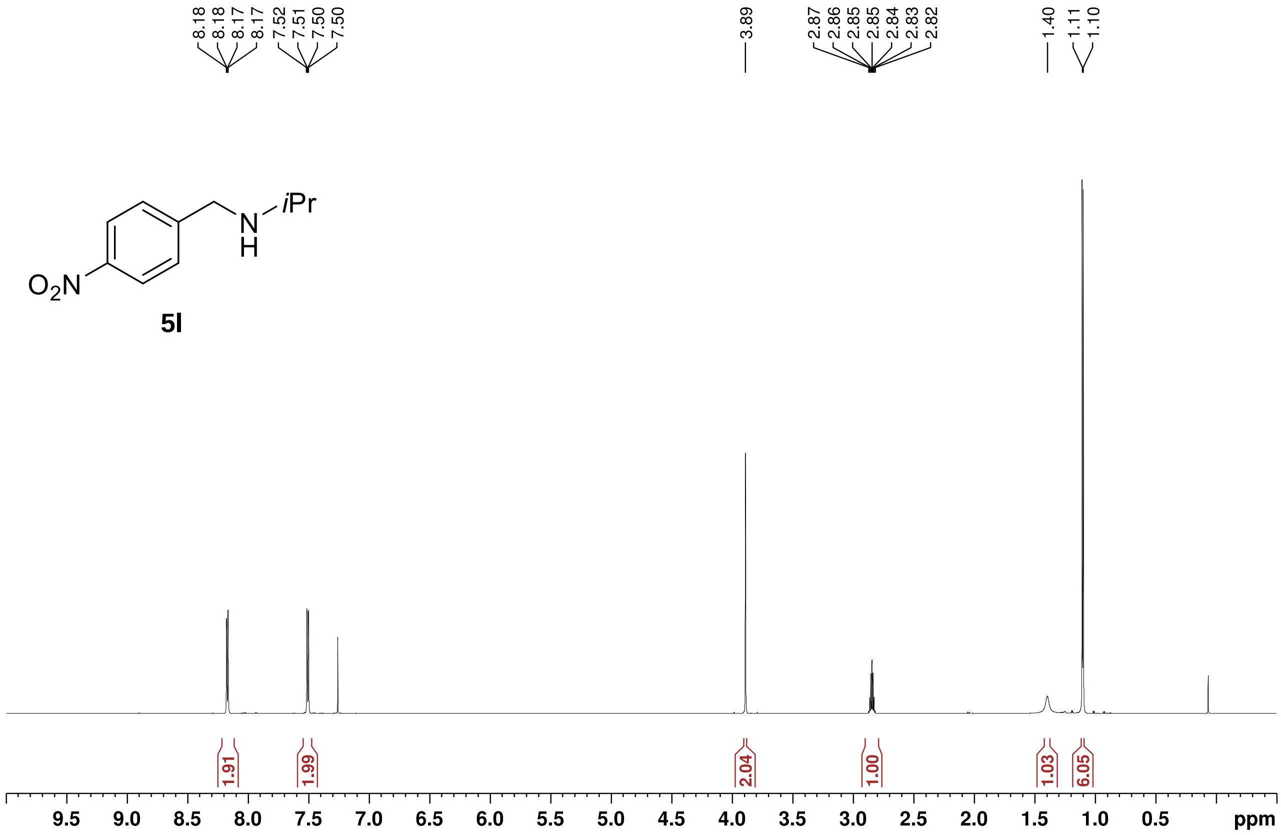

^{13}**C-NMR** (176 MHz, 298 K, $CDCl_3$)

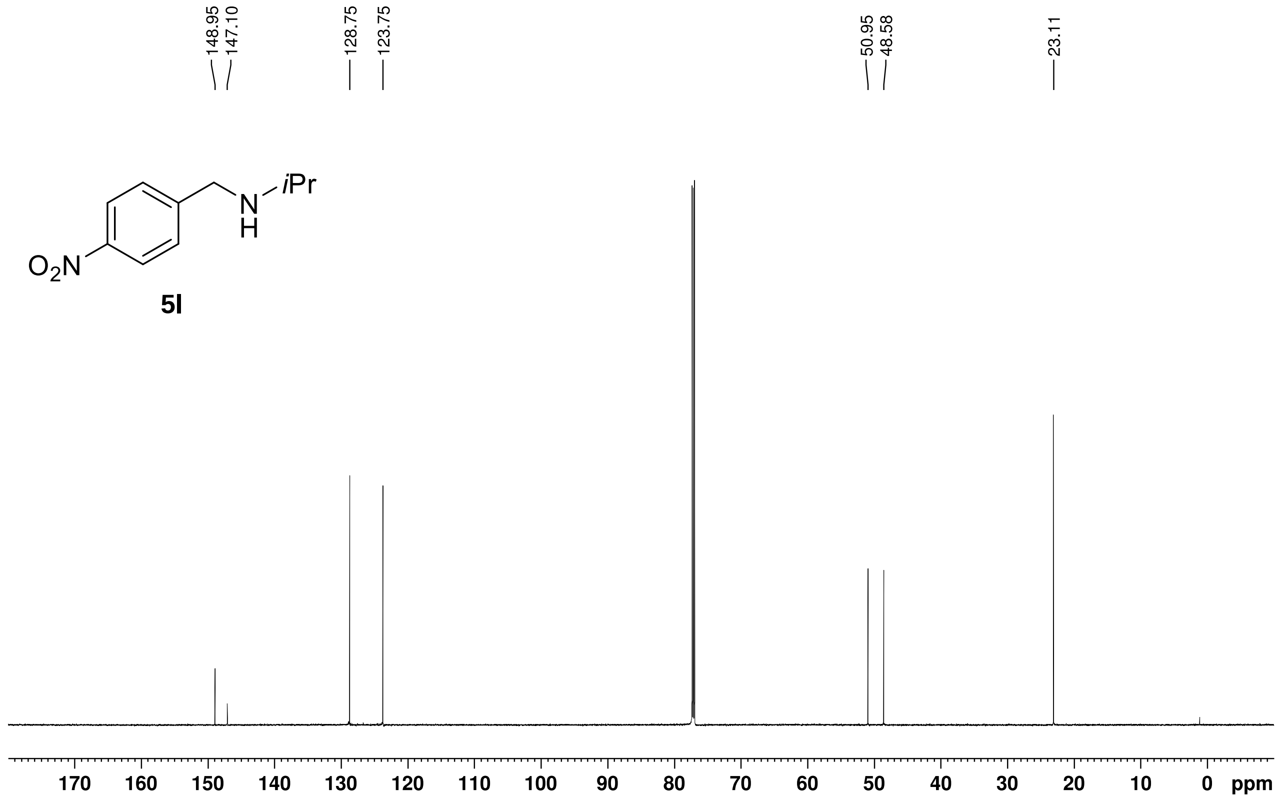

8 NMR Spectroscopic Investigation of Borane Adducts

8.1 $B(2,3,6\text{-}F_3\text{-}C_6H_2)_3$ (4e) and Amide 1a

In a glovebox, $B(2,3,6\text{-}F_3\text{-}C_6H_2)_3$ (**4e**) (4.0 mg, 10 µmol, 1.0 equiv.) and *N*-Isopropyl-4-bromo-benzamide (**1a**) (2.4 mg, 10 µmol, 1.0 equiv.) were dissolved in 0.6 mL $CDCl_3$ and transferred to a J. Young NMR tube. The sample was analyzed by ^{1}H-, ^{11}B- and ^{19}F-NMR spectroscopy and compared to a sample with $B(2,3,6\text{-}F_3C_6H_2)_3$ (4.0 mg, 10 µmol) in $CDCl_3$.

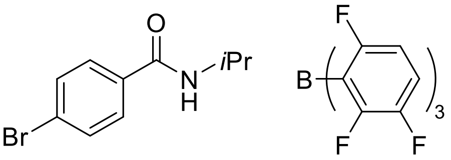

Adduct formation was observed.

^{11}B-NMR (160 MHz, 303 K, $CDCl_3$) δ = 4.8, -1.1; **^{19}F-NMR** (282 MHz, 298 K, $CDCl_3$) δ = -107.8 (br), -108.3 (br), -127.7 (br), -128.4 (br), -144.5 (br), -145.3 (br).

^{11}B-NMR:

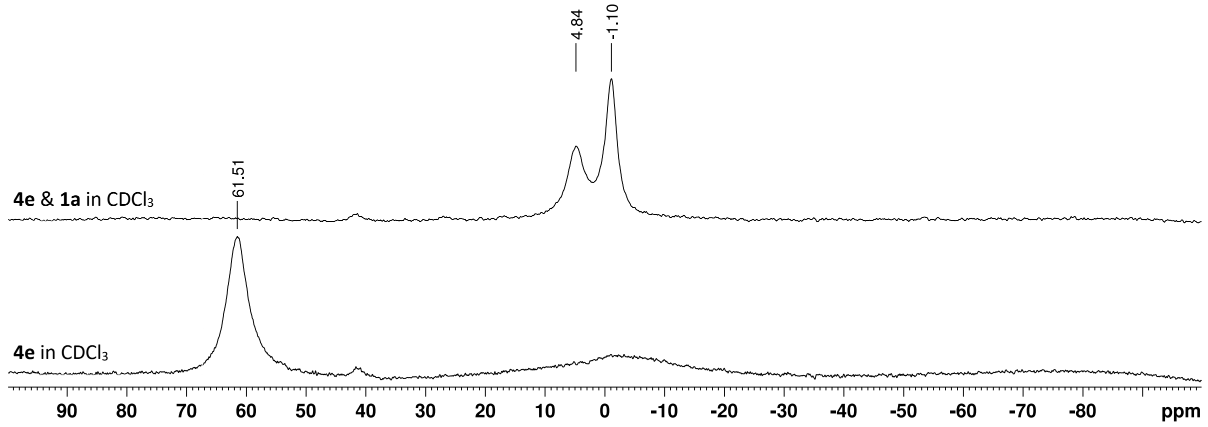

^{19}F-NMR:

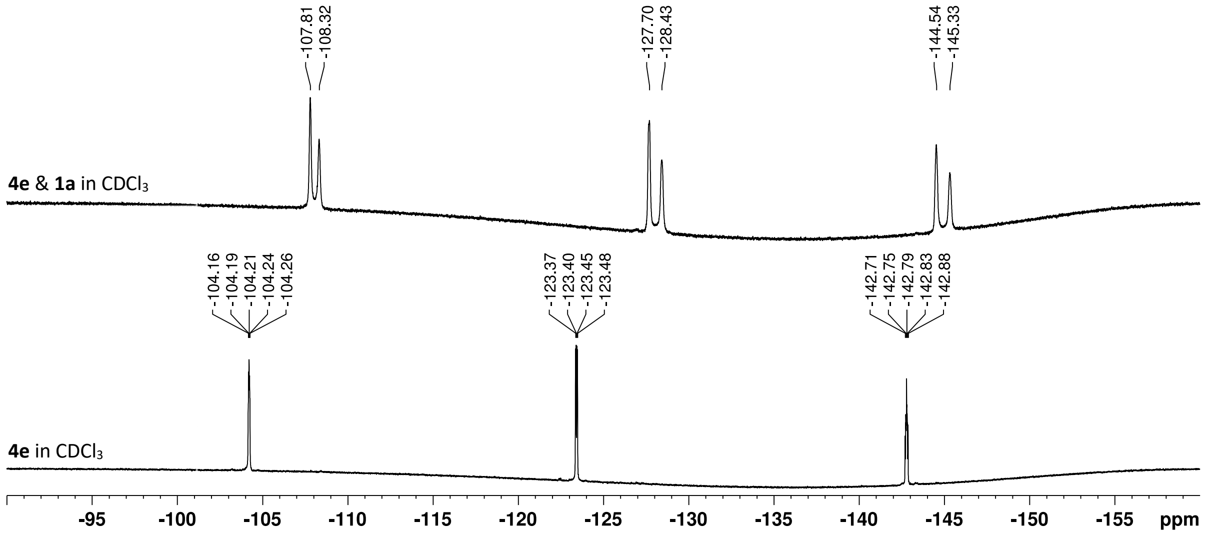

8.2 B(2,3,6-F_3-C_6H_2)$_3$ (4e) and Phosphine Oxides 3a-d

In a glovebox, B(2,3,6-F_3-C_6H_2)$_3$ (**4e**) (4.0 mg, 10 µmol, 1.0 equiv.) and the phosphine oxide **3a-d** (10 µmol, 1.0 equiv.) were dissolved in 0.6 mL $CDCl_3$ and transferred to a J. YOUNG NMR tube. The sample was analyzed by ^{1}H-, ^{11}B-, ^{31}P- and ^{19}F-NMR spectroscopy and compared to a sample with B(2,3,6-$F_3C_6H_2$)$_3$ (4.0 mg, 10 µmol) in $CDCl_3$.

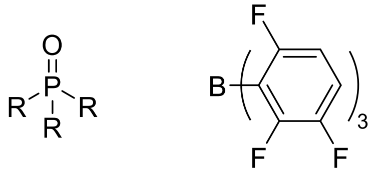

R = Ph (**3a**)
R = 2,6-F_2-C_6H_3 (**3b**)
R = *t*Bu (**3c**)
R = 1-Naphth (**3d**)

Adduct formation of phosphine oxides and B(2,3,6-$F_3C_6H_2$)$_3$ was observed. The broad signals in the ^{19}F- and ^{31}P-NMR spectrum indicate, that the phosphine oxide **3d** has the weakest interaction with the borane. These results are in accordance with 7.2, since **3a** and **3b** had a significant impact on the FLP-catalyzed reduction of an imidoyl chloride, whereas **3c** and **3d** did not.

<u>^{11}B-NMR:</u>

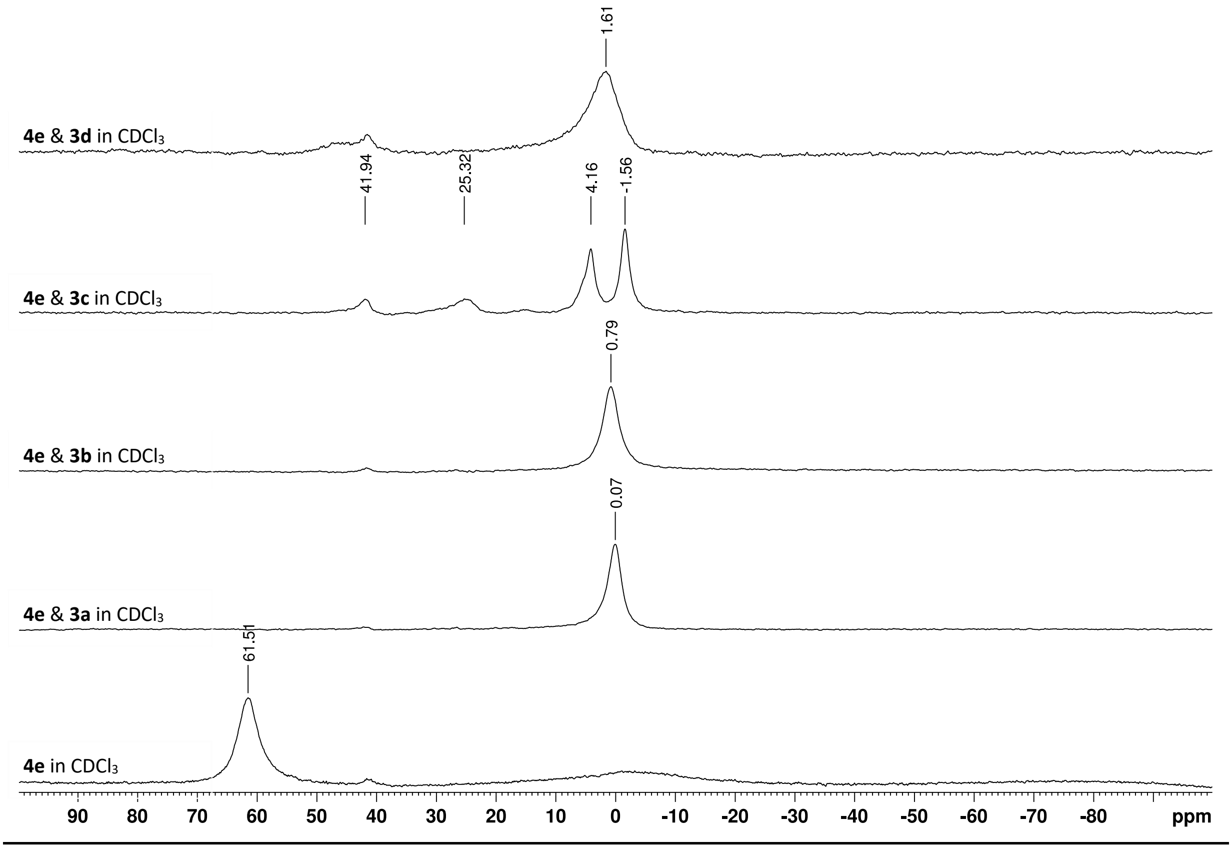

^{19}F-NMR:

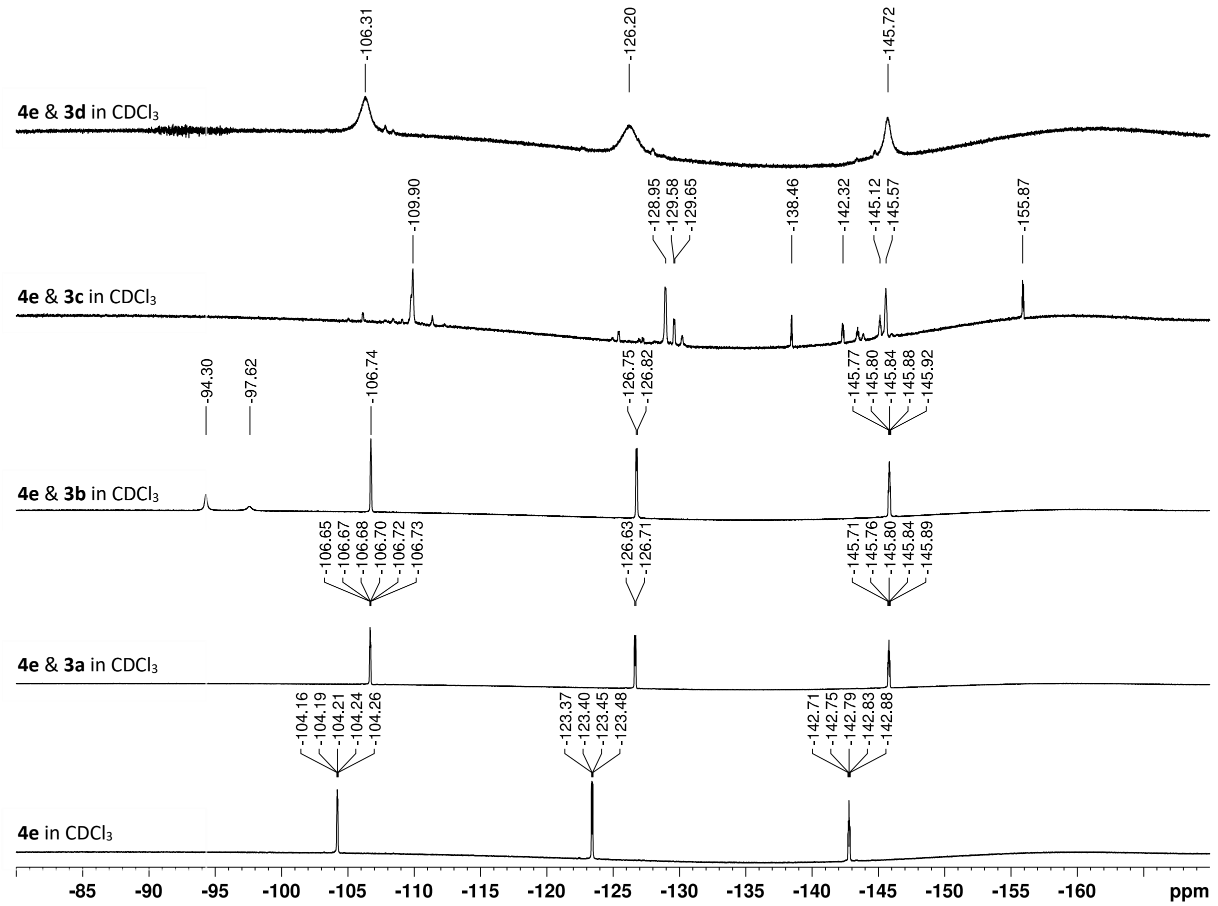

^{31}P-NMR:

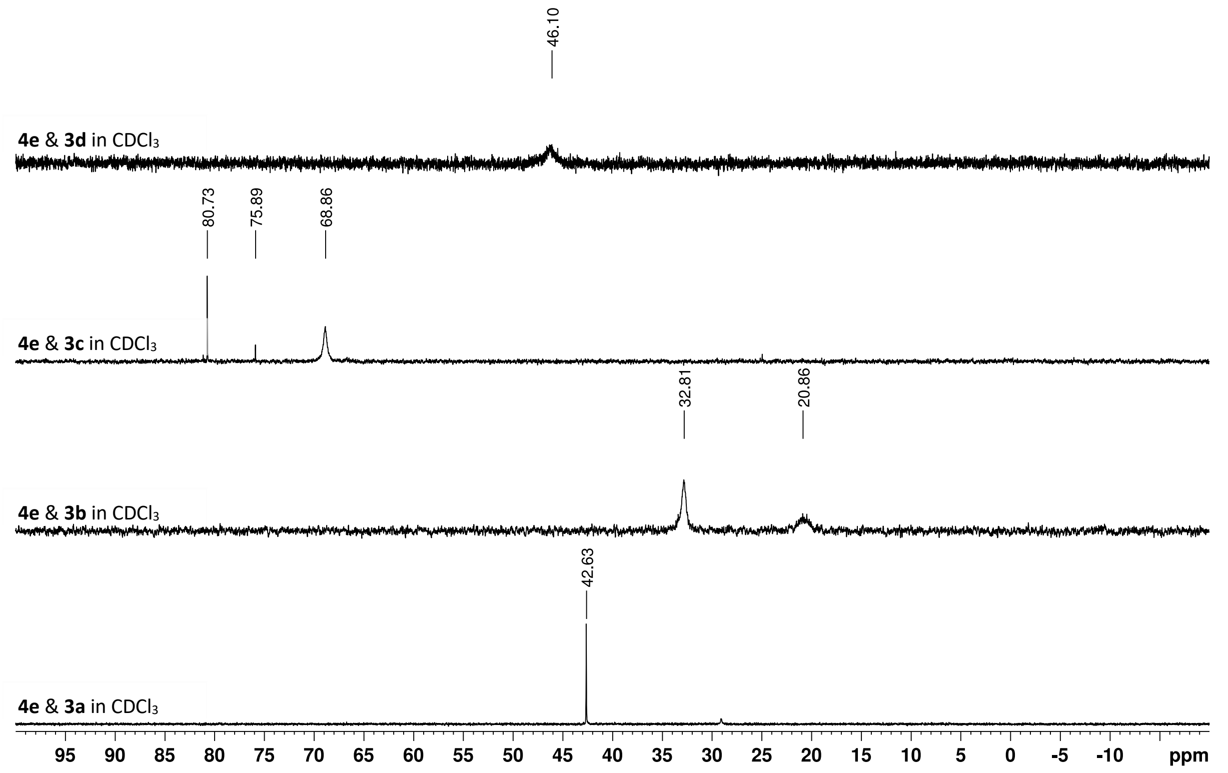

8.3 $B(2,3,6\text{-}F_3\text{-}C_6H_2)_3$ (4e) and Chloride

In a glovebox, $B(2,3,6\text{-}F_3\text{-}C_6H_2)_3$ (**4e**) (4.0 mg, 10 µmol, 1.0 equiv.) and 1-butyl-3-methyl imidazolium chloride (BMIM Cl) (1.7 mg, 10 µmol, 1.0 equiv.) as a chloride source were dissolved in 0.6 mL $CDCl_3$ and transferred to a J. YOUNG NMR tube. The sample was analyzed by ^{1}H-, ^{11}B- and ^{19}F-NMR spectroscopy and compared to a sample with $B(2,3,6\text{-}F_3\text{-}C_6H_2)_3$ (4.0 mg, 10 µmol) in $CDCl_3$.

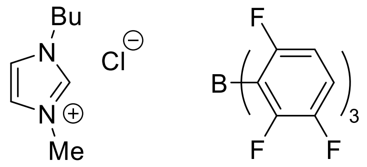

A broadening of the signals in the ^{19}F-NMR spectrum and a change of chemical shifts in the ^{11}B- and ^{19}F-NMR spectrum was observed. This indicates that the chloride is reversibly bound to $B(2,3,6\text{-}F_3\text{-}C_6H_2)_3$.

^{11}B-NMR (160 MHz, 303 K, $CDCl_3$) δ = -5.4; **^{19}F-NMR** (282 MHz, 298 K, $CDCl_3$) δ = -105.9 (br), -126.4 (br), -146.2 – -146.3 (m).

^{11}B-NMR:

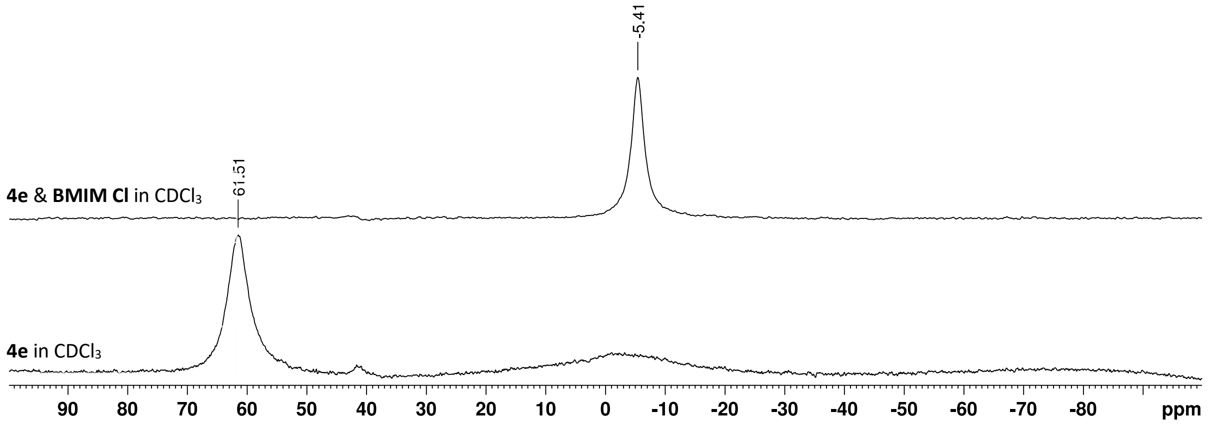

^{19}F-NMR:

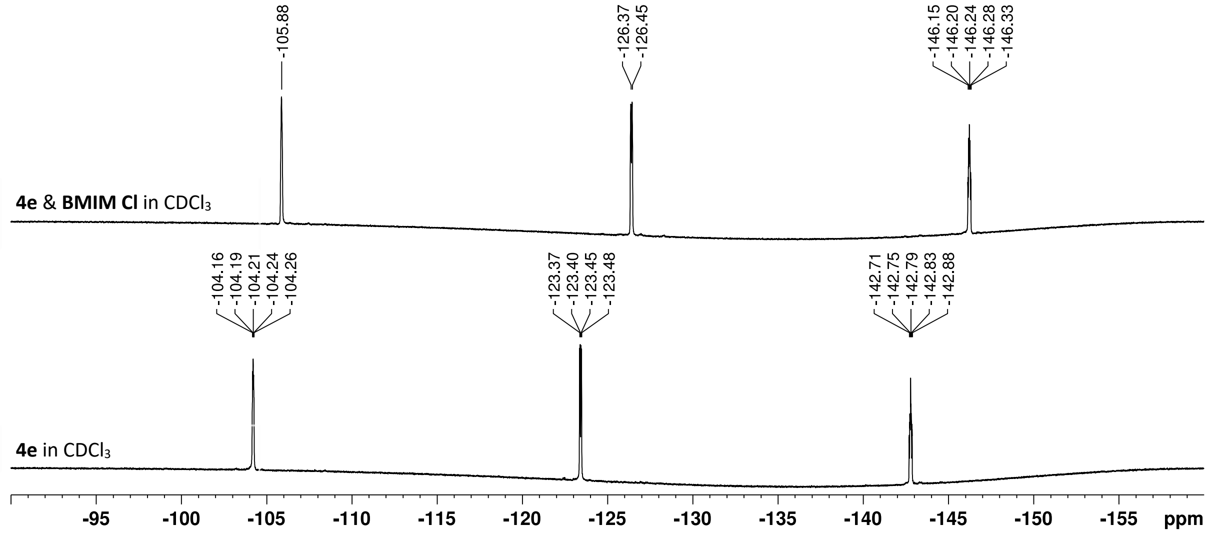

9 Kinetic Investigation

9.1 Hydrogenation of Imidoyl Chloride 2b With Added Ammonium Hydrochloride

In a glovebox, *N*-Isopropylbenzimidoyl chloride (**2b**) (18.2 mg, 100 µmol, 1.00 equiv.), *N*-Isopropylbenzylamine hydrochloride (**5b**) (3.8 mg, 20 µmol, 20 mol%), B(2,3,6-F_3-C_6H_2)$_3$ (**4e**) (4.0 mg, 10 µmol, 10 mol%) and hexamethylbenzene (**HMB**) (1.0 mg, 6.2 µmol, 6.2 mol%), as internal standard, were dissolved in 0.6 mL $CDCl_3$ and transferred to a J. Young NMR tube with Teflon tap. An equal sample without the hydrochloride was prepared as a reference. The samples were then frozen in liquid nitrogen, the headspace was evacuated, and the J. Young NMR tubes was charged with hydrogen at -196 °C. After sealing and thawing, the hydrogen pressure inside the J. Young NMR tubes reached approximately 4 bar. The samples were then heated on a shaking plate to ensure gas exchange. Every 30 minutes (later: every hour) the NMR tubes were cooled to room temperature and analyzed by ^{1}H-NMR spectroscopy. The product concentration was determined by integration against the internal standard.

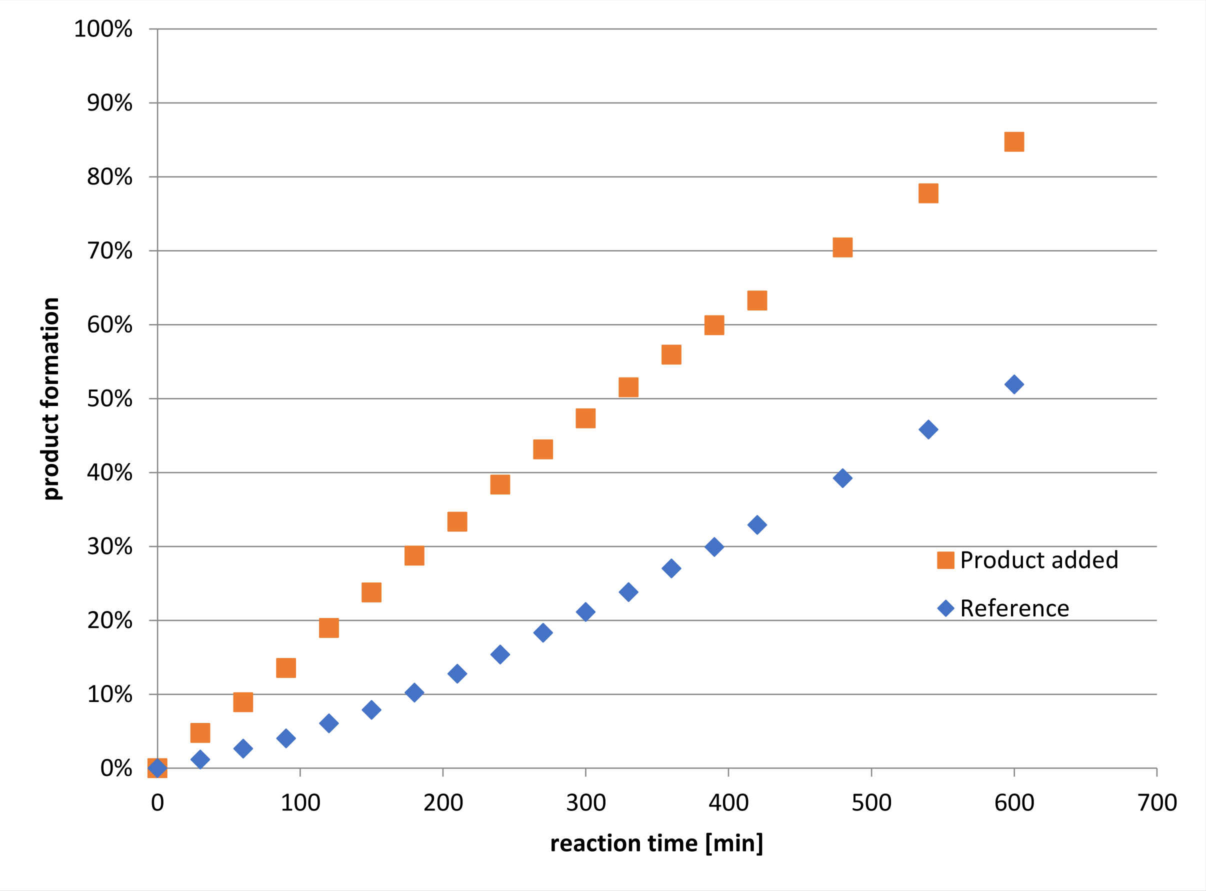

Figure S1: Product formation over time for 10 mol% B(2,3,6-F_3-C_6H_2)$_3$ (**4e**), 20 mol% ammonium chloride **5b** and 4 bar H_2 at 70 °C (red squares). Reference without ammonium chloride **5b** (blue diamonds).

9.2 Hydrogenation of Imidoyl Chloride 2b Without Added Ammonium Hydrochloride

In a glovebox, *N*-Isopropylbenzimidoyl chloride (**2b**) (18.2 mg, 100 µmol, 1.00 equiv.), B(2,3,6-F_3-C_6H_2)$_3$ (**4e**) (4.0 mg, 10 µmol, 10 mol%) and hexamethylbenzene (**HMB**) (1.0 mg, 6.2 µmol, 6.2 mol%), as internal standard, were dissolved in 0.6 mL $CDCl_3$ and transferred to a J. Young NMR tube with Teflon tap. The sample was then frozen in liquid nitrogen, the headspace was evacuated, and the J. Young NMR tube was charged with hydrogen at -196 °C. After sealing and thawing, the hydrogen pressure inside the J. Young NMR tube reached approximately 4 bar. The sample was then transferred to a pre-heated NMR spectrometer and placed inside the spectrometer at 60 °C. The spectrometer then proceeded to measure a ^{1}H-NMR spectrum every 1200 seconds for 16 h. The ^{1}H-NMR spectra were then analyzed, and the product concentration was determined by integration against the internal standard.

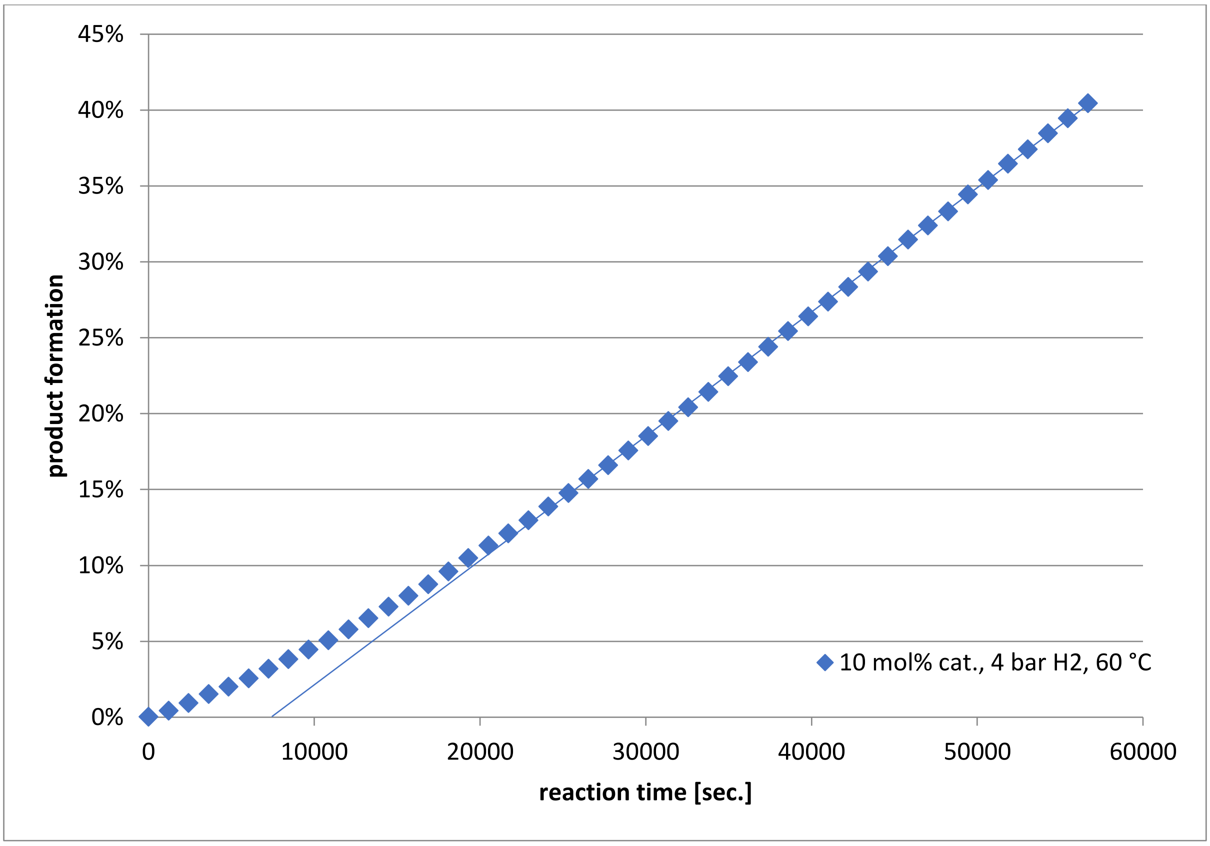

Figure S2: Product formation over time for 10 mol B(2,3,6-F_3-C_6H_2)$_3$ (**4e**) and 4 bar H_2 at 60 °C.

10 References

[SI1] a) L. Greb, C.-G. Daniliuc, K. Bergander, J. Paradies, *Angew. Chem. Int. Ed.* **2013**, *52*, 5876–5879; b) S. Tussing, *Frustrierte Lewispaare: von autoinduzierter Hydrierung von Iminen zur FLP-Reaktivitätsskala*. Dissertation, Shaker Verlag, Aachen, **2017**.

[SI2] a) M. Ullrich, A. J. Lough, D. W. Stephan, *J. Am. Chem. Soc.* **2009**, *131*, 52–53; b) M. Ullrich, A. J. Lough, D. W. Stephan, *Organometallics* **2010**, *29*, 3647–3654; c) J. A. Nicasio, S. Steinberg, B. Inés, M. Alcarazo, *Chem. Eur. J.* **2013**, *19*, 11016–11020.

[SI3] A. J. Stepen, M. Bursch, S. Grimme, D. W. Stephan, J. Paradies, *Angew. Chem. Int. Ed.* **2018**, *57*, 15253–15256.

[SI4] S. Zhou, K. Junge, D. Addis, S. Das, M. Beller, *Angew. Chem. Int. Ed.* **2009**, *48*, 9507–9510.

[SI5] A. Martínez, P. García-García, M. A. Fernández-Rodríguez, F. Rodríguez, R. Sanz, *Angew. Chem. Int. Ed.* **2010**, *49*, 4633–4637.

[SI6] J. Gawronski, K. Gawronska, P. Skowronek, A. Holmén, *J. Org. Chem.* **1999**, *64*, 234–241.

11 Computational Details

11.1 General remarks, geometry optimizations, energy calculations and vibrational frequency calculations

All visualizations of molecular structures were created with UCSF Chimera[S1] 1.10.2. Quantum mechanical calculations were performed with the TURBOMOLE 7.3.1[S2,S3] and xtb 6.2.2[S4] program packages. Geometries were pre-optimized with the GFN2-xTB[S5] extended tight binding method with applied GBSA[S6] implicit solvation model for $CHCl_3$. Final optimization was conducted applying the PBEh-3c[S7] composite method combined with the COSMO[S8] implicit continuum solvation contribution for $CHCl_3$ (ε = 4.71). The numerical quadrature grid m4 was employed for the integration of the exchange-correlation contributions and default convergence criteria for energies and gradients were applied as implemented in TURBOMOLE. Minimum structures were verified as minima on the potential energy hyper surface by the absence of imaginary frequencies ($i\omega$ > 35 cm^{-1}) in the numerical harmonic vibrational frequency calculation. If present imaginary frequencies below this threshold were inverted and included in the thermostatistical correction.

All geometry optimizations and single point calculations were performed applying either the resolution-of-identity (RI) approximation for Coulomb integrals[S9] (RIJ, optimizations) or for Coulomb and exchange integrals (RIJK, single point calculations) with matching default auxiliary basis sets.[S10]

The D3[S11] (incorporated in the PBEh-3c composite method) and D4[S12–14] London dispersion correction schemes applying Becke-Johnson (BJ) damping[S15,S16] and including Axilrod-Teller-Muto (ATM)[S17,S18] type three-body contributions to the total London dispersion energy were applied. For a review on this topic see Ref.[S19]

Ro-vibrational corrections to obtain free energies were obtained from a modified rigid rotor harmonic oscillator statistical treatment[S20] (T = 70.0 °C, 1 atm pressure) based on harmonic frequencies calculated at the geometry optimization level (PBEh-3c(COSMO($CHCl_3$))). To avoid errors in the harmonic approximation, frequencies with wave numbers below 100 cm^{-1} were treated partially as rigid rotors.[S20]

Gas phase single point energies were calculated at PW6B95-D4/def2-QZVP level applying the m5 numerical quadrature grid.

11.2 Solvation corrections and Gibbs free energies

Solvation effects were further considered by the COSMO-RS[S21,S22] model, used as implemented in COSMOtherm (Version C3.0, release 16.01)[S23] with the 2016 parameterization for $CHCl_3$ (parameter file: BP_TZVP_C30_1601.ctd; default G_{solv} option). Calculated solvation corrections were further corrected for the volume work of 1 bar to 1 M ideal gas. In this framework the default BP86[S24,S25]/def-TZVP[S26] level of theory was used for single point calculations on the optimized geometries.

Final Gibbs free energies were obtained by summing the gas phase single point energy E, the dispersion correction $E_{Disp.}$, the ro-vibrational correction G_{RRHO} and the solvation correction δG_{solv} (Eq. S1).

$$G_{tot.} = E + E_{Disp., D4} + G_{RRHO} + \delta G_{solv., corr.} \qquad \text{(Equation S1)}$$

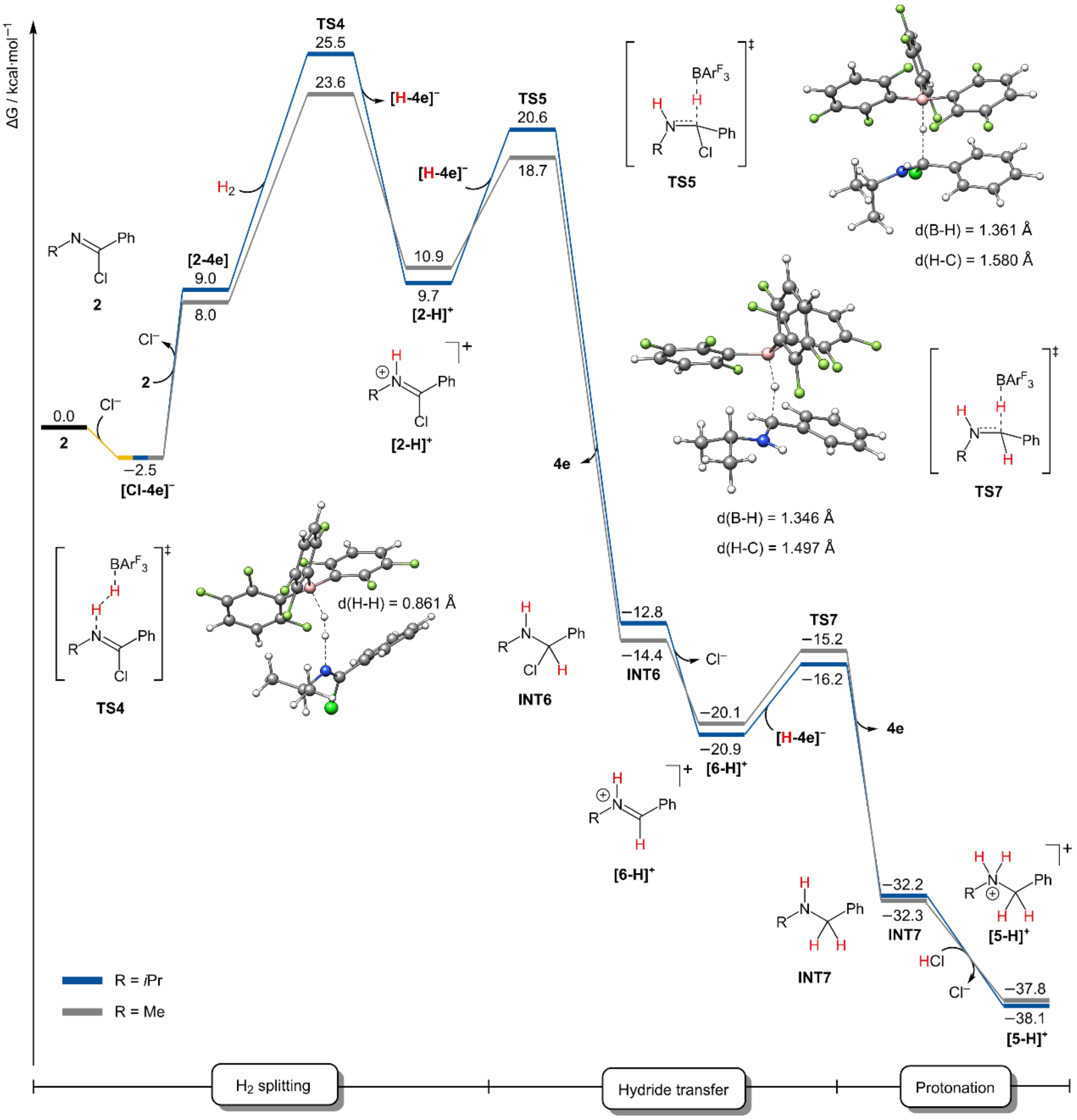

Figure S3: Free energy diagram of the H_2-activation by the FLP **2/4e** at the PW6B95-D4/def2-QZVP+COSMO-RS($CHCl_3$)//PBEh-3c(COSMO($CHCl_3$)) level of theory. All free energies in kcal/mol.

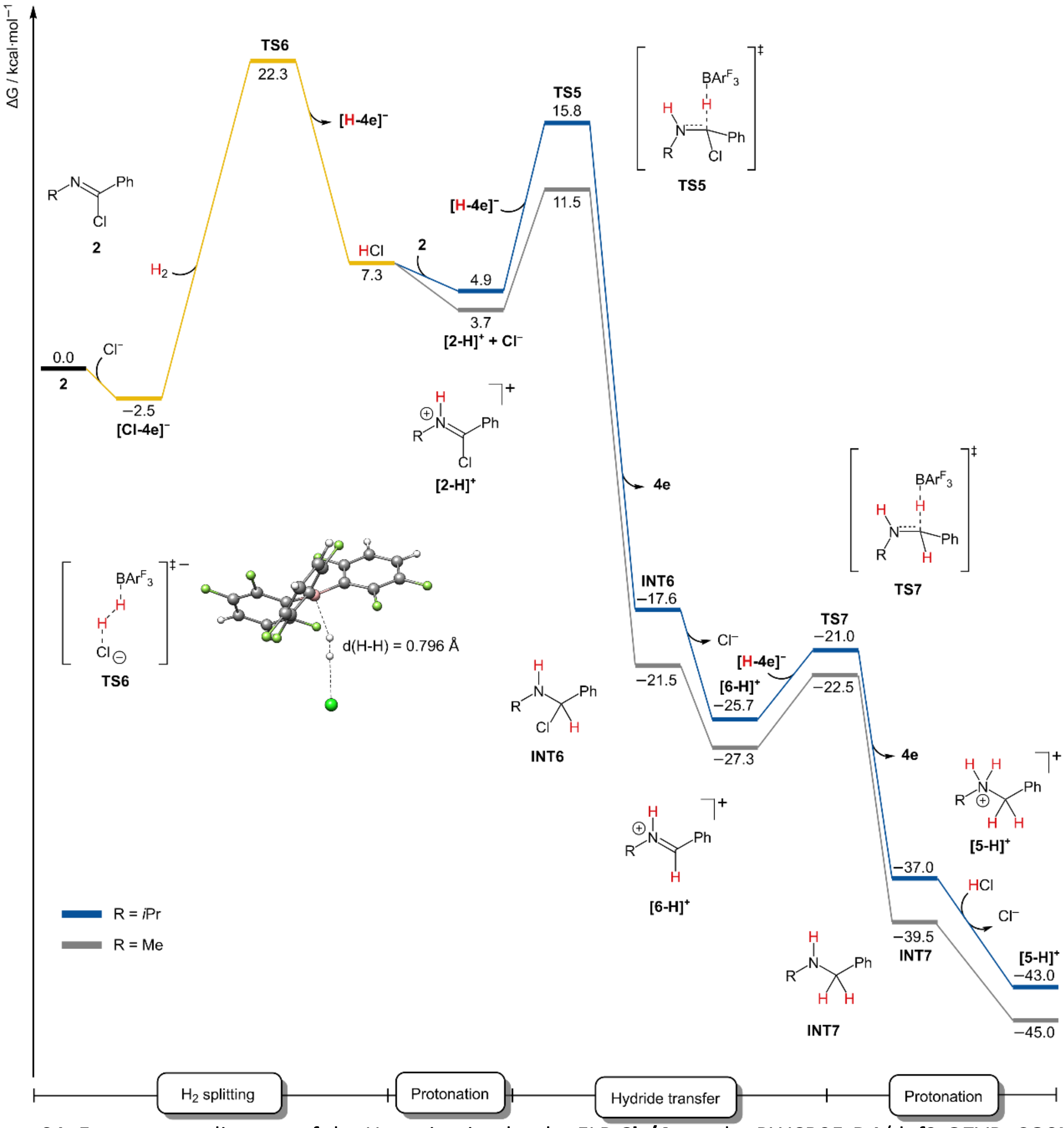

Figure S4: Free energy diagram of the H_2-activation by the FLP **Cl⁻/4e** at the PW6B95-D4/def2-QZVP+COSMO-RS($CHCl_3$)//PBEh-3c(COSMO($CHCl_3$)) level of theory. All free energies in kcal/mol.

11.3 Energy contributions

Table S4: Absolute contributions to $G_{tot.}$.

Structure	Sym.	Imaginary frequency	E(PBEh-3c(COSMO($CHCl_3$))) / a. u.	E(PW6B95/def2-QZVP) / a. u.	E(PW6B95/def2-QZVP) / kcal mol^{-1}	$E_{Disp., D4}$ / kcal mol^{-1}	G_{RRHO} (70.0 °C) / kcal mol^{-1}	$\delta G_{solv., corr.}$ ($CHCl_3$, 70.0 °C) / kcal mol^{-1}	$G_{tot.}$ / kcal mol^{-1}
**1b**	C_1	0.0	-517.77557	-519.77945	-326166.53108	-13.925	104.242	-8.962	-326085.176
**1e**	C_1	0.0	-439.31289	-441.01332	-276740.03627	-10.543	71.553	-8.301	-276687.327
**2b**	C_1	0.0	-901.77418	-904.38924	-567512.81605	-14.605	94.614	-5.775	-567438.582
**2e**	C_s	0.0	-823.31079	-825.62347	-518086.54782	-11.155	61.882	-4.839	-518040.660
**[2b-H]$^+$**	C_1	0.0	-902.19171	-904.76166	-567746.51590	-14.310	102.918	-42.853	-567700.761
**[2e-H]$^+$**	C_1	0.0	-823.72757	-825.99078	-518317.04325	-10.915	70.507	-44.235	-518301.686
**[2b-4e]**	C_1	0.0	-2511.46940	-2519.96671	-1581302.98450	-48.702	199.639	-16.694	-1581168.741
**[2e-4e]**	C_1	0.0	-2433.00655	-2441.20227	-1531877.55083	-45.304	166.913	-15.876	-1531771.818
**3a**	C_1	0.0	-1109.47523	-1113.21741	-698554.47387	-28.466	140.067	-16.169	-698459.042
**EPC**	C_1	0.0	-1493.91912	-1498.22673	-940151.46871	-28.920	138.226	-41.440	-940083.603
**4e**	C_1	0.0	-1609.68620	-1615.57575	-1013789.08970	-27.989	88.073	-10.136	-1013739.142
**[Cl-4e]$^-$**	C_1	0.0	-2069.56860	-2076.29258	-1302893.26347	-32.007	87.340	-47.235	-1302885.165
**[H-4e]$^-$**	C_1	0.0	-1610.41070	-1616.26750	-1014223.17113	-29.196	93.061	-44.795	-1014204.101
**[5b-H]$^+$**	C_1	0.0	-444.31422	-445.99369	-279865.26465	-13.872	125.665	-46.155	-279799.627
**[5e-H]$^+$**	C_s	0.0	-365.85070	-367.22234	-230435.49777	-9.979	93.216	-49.068	-230401.329
**[6b-H]$^+$**	C_1	0.0	-443.09541	-444.78601	-279107.43365	-12.592	110.532	-43.287	-279052.780
**[6e-H]$^+$**	C_s	0.0	-364.63142	-366.01525	-229678.03590	-9.214	77.975	-44.774	-229654.049
**Cl^-**	O_h	0.0	-459.82626	-460.64663	-289060.12473	0.000	-11.098	-72.325	-289143.547
**H_2**	D_{6h}	0.0	-1.16818	-1.17592	-737.90367	-0.037	-2.536	3.601	-736.876
**HCl**	C_{6v}	0.0	-460.30096	-461.18816	-289399.93830	-0.123	-9.154	1.055	-289408.160
**INT1b**	C_1	0.0	-2011.71067	-2018.02089	-1266327.22812	-50.799	259.257	-45.775	-1266164.545
**INT2b**	C_1	0.0	-2471.58417	-2478.78903	-1555463.59997	-55.733	259.247	-34.972	-1555295.058
**INT3b**	C_1	0.0	-2011.67569	-2017.97273	-1266297.00624	-50.846	259.410	-59.007	-1266147.450
**INT4b**	C_1	0.0	-1551.36176	-1556.79266	-976902.14095	-48.570	253.566	-43.523	-976740.668
**INT5b**	C_1	0.0	-441.85119	-443.54731	-278330.13851	-11.272	94.960	-41.701	-278288.152
**INT6b**	C_1	0.0	-902.98904	-905.60286	-568274.37583	-15.498	109.062	-7.437	-568188.248
**INT6e**	C_1	0.0	-824.52748	-826.83754	-518848.38742	-11.888	75.309	-6.972	-518791.939
**INT7b**	C_1	0.0	-443.87253	-445.61135	-279625.34548	-13.756	115.747	-5.679	-279529.034
**INT7e**	C_1	0.0	-365.41070	-366.84606	-230199.37712	-10.331	83.276	-4.811	-230131.244
**TS1b**	C_1	-63.0	-2471.55679	-2478.76421	-1555448.02561	-53.105	257.489	-41.648	-1555285.290
**TS2b**	C_1	-544.5	-2011.67181	-2017.97814	-1266300.40132	-50.243	256.227	-52.005	-1266146.423
**TS3b**	C_1	-201.5	-1551.33872	-1556.78317	-976896.18561	-46.637	251.678	-42.812	-976733.957
**TS4b**	C_1	-276.7	-2512.61848	-2521.12441	-1582029.45478	-51.596	209.196	-17.293	-1581889.148
**TS4e**	C_1	-192.5	-2434.15582	-2442.36322	-1532606.06211	-46.883	176.187	-16.305	-1532493.063
**TS5b**	C_1	-512.3	-2512.63196	-2521.13246	-1582034.50507	-54.210	213.057	-18.311	-1581893.969
**TS5e**	C_1	-471.0	-2434.17196	-2442.36924	-1532609.83848	-49.972	180.247	-18.386	-1532497.949
**TS6**	C_1	-96.2	-2070.67874	-2077.41576	-1303598.07203	-30.651	94.513	-63.018	-1303597.228
**TS7b**	C_1	-281.0	-2053.54238	-2061.16959	-1293403.44796	-50.744	220.095	-18.081	-1293252.178
**TS7e**	C_1	-263.5	-1975.07899	-1982.40238	-1243976.27429	-46.361	187.405	-18.094	-1243853.324

11.4 References for Computational Details

(S1) Pettersen, E. F.; Goddard, T. D.; Huang, C. C.; Couch, G. S.; Greenblatt, D. M.; Meng, E. C.; Ferrin, T. E. UCSF Chimera - A Visualization System for Exploratory Research and Analysis. *J. Comput. Chem.* **2004**, *25* (13), 1605–1612. https://doi.org/10.1002/jcc.20084.

(S2) Furche, F.; Ahlrichs, R.; Hättig, C.; Klopper, W.; Sierka, M.; Weigend, F. Turbomole. *WIREs Comput Mol Sci* **2014**, *4*, 91–100.

(S3) TURBOMOLE V7.3.1 2018, a Development of Univesity of Karlsruhe and Forschungszentrum Karlsruhe GmbH, 1989-2007, TURBOMOLE GmbH, since 2007; Available from Http://Www.Turbomole.Com.

(S4) Semiempirical Extended Tight-Binding Program Package Xtb, Version 6.2, Https://Github.Com/Grimme-Lab/Xtb. Accessed: 2020-05-03.

(S5) Bannwarth, C.; Ehlert, S.; Grimme, S. GFN2-XTB - An Accurate and Broadly Parametrized Self-Consistent Tight-Binding Quantum Chemical Method with Multipole Electrostatics and Density-Dependent Dispersion Contributions. *J. Chem. Theory Comput.* **2019**, *15* (3), 1652–1671. https://doi.org/10.1021/acs.jctc.8b01176.

(S6) Bannwarth, C.; Caldeweyher, E.; Ehlert, S.; Hansen, A.; Pracht, P.; Seibert, J.; Spicher, S.; Grimme, S. Extended Tight-Binding Quantum Chemistry Methods. *Wiley Interdisciplinary Reviews: Computational Molecular Science*. Blackwell Publishing Inc. 2020. https://doi.org/10.1002/wcms.1493.

(S7) Grimme, S.; Brandenburg, J. G.; Bannwarth, C.; Hansen, A. Consistent Structures and Interactions by Density Functional Theory with Small Atomic Orbital Basis Sets. *J. Chem. Phys.* **2015**, *143* (5), 054107. https://doi.org/10.1063/1.4927476.

(S8) Klamt, A.; Schüürmann, G. COSMO: A New Approach to Dielectric Screening in Solvents with Explicit Expressions for the Screening Energy and Its Gradient. *J. Chem. Soc., Perkin Trans. 2* **1993**, *0* (5), 799–805. https://doi.org/10.1039/P29930000799.

(S9) Eichkorn, K.; Treutler, O.; Öhm, H.; Häser, M.; Ahlrichs, R. Auxiliary Basis Sets to Approximate Coulomb Potentials. *Chem. Phys. Lett.* **1995**, *240* (4), 283–289. https://doi.org/10.1016/0009-2614(95)00621-A.

(S10) Weigend, F. Accurate Coulomb-Fitting Basis Sets for H to Rn. *Phys. Chem. Chem. Phys.* **2006**, *8* (9), 1057. https://doi.org/10.1039/b515623h.

(S11) Grimme, S.; Antony, J.; Ehrlich, S.; Krieg, H. A Consistent and Accurate Ab Initio Parametrization of Density Functional Dispersion Correction (DFT-D) for the 94 Elements H-Pu. *J. Chem. Phys.* **2010**, *132* (15), 154104. https://doi.org/10.1063/1.3382344.

(S12) Caldeweyher, E.; Ehlert, S.; Hansen, A.; Neugebauer, H.; Spicher, S.; Bannwarth, C.; Grimme, S. A Generally Applicable Atomic-Charge Dependent London Dispersion Correction. *J. Chem. Phys.* **2019**, *150* (15), 154122. https://doi.org/10.1063/1.5090222.

(S13) Caldeweyher, E.; Bannwarth, C.; Grimme, S. Extension of the D3 Dispersion Coefficient Model. *J. Chem. Phys.* **2017**, *147* (3), 034112. https://doi.org/10.1063/1.4993215.

(S14) Bursch, M.; Caldeweyher, E.; Hansen, A.; Neugebauer, H.; Ehlert, S.; Grimme, S. Understanding and Quantifying London Dispersion Effects in Organometallic Complexes. *Acc. Chem. Res.* **2019**, *52* (1), 258–266. https://doi.org/10.1021/acs.accounts.8b00505.

(S15) Grimme, S.; Ehrlich, S.; Goerigk, L. Effect of the Damping Function in Dispersion Corrected Density Functional Theory. *J. Comput. Chem.* **2011**, *32* (7), 1456–1465. https://doi.org/10.1002/jcc.21759.

(S16) Becke, A. D.; Johnson, E. R. A Density-Functional Model of the Dispersion Interaction. *J. Chem. Phys.* **2005**, *123* (15), 154101. https://doi.org/10.1063/1.2065267.

(S17) Axilrod, B. M.; Teller, E. Interaction of the van Der Waals Type between Three Atoms. *J. Chem. Phys.* **1943**, *11* (6), 299–300. https://doi.org/10.1063/1.1723844.

(S18) Muto, Y. Force between Nonpolar Molecules. *Proc. Phys. Math. Soc. Jpn.* **1943**, *17*, 629–631.

(S19) Grimme, S.; Hansen, A.; Brandenburg, J. G.; Bannwarth, C. Dispersion-Corrected Mean-Field Electronic Structure Methods. *Chem. Rev.* **2016**, *116* (9), 5105–5154. https://doi.org/10.1021/acs.chemrev.5b00533.

(S20) Grimme, S. Supramolecular Binding Thermodynamics by Dispersion-Corrected Density Functional Theory. *Chem. - A Eur. J.* **2012**, *18* (32), 9955–9964. https://doi.org/10.1002/chem.201200497.

(S21) Klamt, A. Conductor-like Screening Model for Real Solvents: A New Approach to the Quantitative Calculation of Solvation Phenomena. *J. Phys. Chem.* **1995**, *99* (7), 2224–2235. https://doi.org/10.1021/j100007a062.

(S22) Eckert, F.; Klamt, A. Fast Solvent Screening via Quantum Chemistry: COSMO-RS Approach. *AIChE J.* **2002**, *48* (2), 369–385. https://doi.org/10.1002/aic.690480220.

(S23) Klamt, A.; Eckert, F.; Pohler, L. COSMOtherm. COSMOlogic GmbH & Co. KG: Leverkusen, Germany 2013.

(S24) Becke, A. D. Density-Functional Exchange-Energy Approximation with Correct Asymptotic Behavior. *Phys. Rev. A* **1988**, *38* (6), 3098–3100. https://doi.org/10.1103/PhysRevA.38.3098.

(S25) Perdew, J. P. Density-Functional Approximation for the Correlation Energy of the Inhomogeneous Electron Gas. *Phys. Rev. B* **1986**, *33* (12), 8822–8824.

https://doi.org/10.1103/PhysRevB.33.8822.

(S26) Schäfer, A.; Huber, C.; Ahlrichs, R.; Schafer, A.; Huber, C.; Ahlrichs, R. Fully Optimized Contracted Gaussian Basis Sets of Triple Zeta Valence Quality for Atoms Li to Kr Fully Optimized Contracted Gaussian Basis Sets of Triple Zeta Valence Quality for Atoms Li to Kr. *J. Chem. Phys.* **1994**, *100* (8), 5829–5835. https://doi.org/10.1063/1.467146.
